# Growth hormone-mediated reprogramming of macrophage transcriptome and effector functions

**DOI:** 10.1038/s41598-019-56017-6

**Published:** 2019-12-18

**Authors:** Augusto Schneider, Hillary N. Wood, Sandra Geden, Catherine J. Greene, Robin M. Yates, Michal M. Masternak, Kyle H. Rohde

**Affiliations:** 1 Faculdade de Nutrição, Universidade Federal de Pelotas, Pelotas, RS Brazil; 2 College of Medicine, Burnett School of Biomedical Sciences, University of Central Florida, Orlando, FL 32827 USA; 3 Department of Biochemistry and Molecular Biology, Cumming School of Medicine, University of Calgary, Calgary, Alberta Canada; 4 Department of Comparative Biology and Experimental Medicine, Faculty of Veterinary Medicine, University of Calgary, Calgary, Alberta Canada; 5 Department of Head and Neck Surgery, The Greater Poland Cancer Centre, Poznan, Poland

**Keywords:** Gene regulation in immune cells, Monocytes and macrophages, Ageing

## Abstract

Macrophages are an important component of the innate immune response. Priming and activation of macrophages is stimulated by cytokines (i.e IFNγ). However, growth hormone (GH) can also stimulate macrophage activation. Based on these observations, the goal of this work was to 1) to compare the transcriptome profile of macrophages activated *in vitro* with GH and IFNγ, and 2) to assess the impact of GH on key macrophage functional properties like reactive oxygen species (ROS) production and phagosomal proteolysis. To assess the global transcriptional and functional impact of GH on macrophage programming, bone marrow derived macrophages were treated with GH or IFNγ. Our data strongly support a potential link between GH, which wanes with age, and impaired macrophage function. The notable overlap of GH with IFNγ-induced pathways involved in innate immune sensing of pathogens and antimicrobial responses argue for an important role for GH in macrophage priming and maturation. By using functional assays that report on biochemical activities within the lumen of phagosomes, we have also shown that GH alters physiologically relevant processes such as ROS production and proteolysis. These changes could have far reaching impacts on antimicrobial capacity, signaling, and antigen presentation.

## Introduction

Growth hormone (GH) is well recognized for promoting somatic body growth^1^, and its main actions are mediated by liver produced insulin-like growth factor (IGF-I), through binding to the GH receptor (GHR)^2^. GHR is a transmembrane protein member of the cytokine receptor family^3^, which includes receptors for interleukin 2, 3, 4, 5, 6 and 7^3,4^. Besides the liver, several cell types in the body including macrophages express GHR on their surface and are directly responsive to GH^3^. Pituitary GH secretion declines with age during adult life^5^, and has been implicated as a key factor in the development of age related diseases. This decline in GH secretion with age is paralleled by a generalized decline of immune function, a process known as immunosenescence^6^.

Growth hormone deficient Ames dwarf mice (df/df) mice have a genetic mutation that leads to impaired anterior pituitary gland development^7^. As a consequence, these mice have very low circulating GH and IGF-I levels, reduced adult body size^8^, and can live up to 65% longer than wild-type (WT) littermates^9^. The df/df mice have been extensively characterized as having a reduced pro-inflammatory profile as they age, which may represent the major mechanism promoting extended lifespan^10^. On the contrary, this phenotype might reflect impairment of the innate immune system, which could increase susceptibility to infectious disease. Mice with disruption of the GH releasing hormone gene have lower proportion of lung macrophages than WT mice, however in the spleen more macrophages were observed^11^. These mice also exhibited increased susceptibility to *S. pneumoniae* infection with a time-dependent increase in lung bacterial load^11^. GH-deficient Snell dwarf mice, despite having absent GH pituitary secretion, do have expression of GH in bone marrow cells^12^. Therefore, it is of interest to define the pathways and genes regulated by GH in key cells of the innate immune system such as macrophages. If a strong link is established between GH and optimal function of innate immune cells, these mice would serve as a valuable model to explore the role of GH in resistance to infectious diseases.

Macrophages are an important component of the innate immune response, and are central to pathogen recognition and response^13^. Priming and activation of macrophages by cytokines (i.e IFNγ) and encounters with pathogens exert profound effects on their antimicrobial and immune modulatory capacity. This can reprogram gene expression and cytokine production, trigger oxidative and nitrosative bursts, change the makeup of the phagosome lumen (pH, ions, hydrolytic enzymes), and alter trafficking of pathogens and antigen processing^14–21^. Interestingly, it was demonstrated that macrophages can be primed by GH similarly to IFNγ, a well-characterized macrophage activator^22–24^. Treatment of monocytes *in vitro* with GH increased secretion of pro-inflammatory cytokines, interleukin (IL)-1α, IL-6 and TNFα^25^ and enhanced macrophage function *in vivo* by increasing superoxide and TNFα release^23,24^. This suggests that GH has functions that at least partially overlap with IFNγ and may impact the ability of the host to respond to an infection. Although it is known that GH can prime macrophages similarly to IFNγ^22^ the specific pathways activated by GH and the extent of this response are not well understood.

Based on the presented evidence, the goal of this work was to 1) to compare the transcriptome profile of macrophages derived from WT and GH-deficient df/df mice, 2) to compare the transcriptome profile of macrophages activated *in vitro* with GH or IFNγ, and 3) to assess the impact of GH on biochemical processes within macrophage phagosomes such as reactive oxygen species production and phagosomal proteolysis that are critical for their role in innate immunity. Given that earlier studies only determined the GH-dependent regulation of a few select genes^25^, we chose to apply next generation sequencing to generate global RNASeq transcriptome profiles of GH versus IFNγ activated macrophages from WT and df/df mice. Although there were minimal differences in the transcriptome of untreated macrophages derived from WT versus df/df mice, GH treatment induced a significant transcriptional reprogramming that included pathways that overlapped with IFNγ as well as unique GH specific pathways. Our data showing the ability of GH to impact phagosomal processes involved in antimicrobial defense and antigen presentation corroborate the RNAseq results and suggest an important role for GH in macrophage priming and activation.

## Results

### Real time RT-PCR for Tnf mRNA and sample selection

We assessed the levels of *Tnf* mRNA by real-time RT-PCR to determine the optimal GH dose and treatment duration before processing samples for a more in depth RNASeq analysis. GH treatment at 500 ng/mL and IFNγ at 10 ng/mL at the 6 h time point produced significant changes in *Tnf* mRNA expression in comparison to Control samples independent of genotype (P < 0.05; Suppl. Fig. 1). Therefore, WT and df/df samples from Control, GH at 500 ng/mL and IFN at 10 ng/mL, 6 h after treatment, were selected for further analysis by RNASeq.

### RNA sequencing results

**Figure 1:**
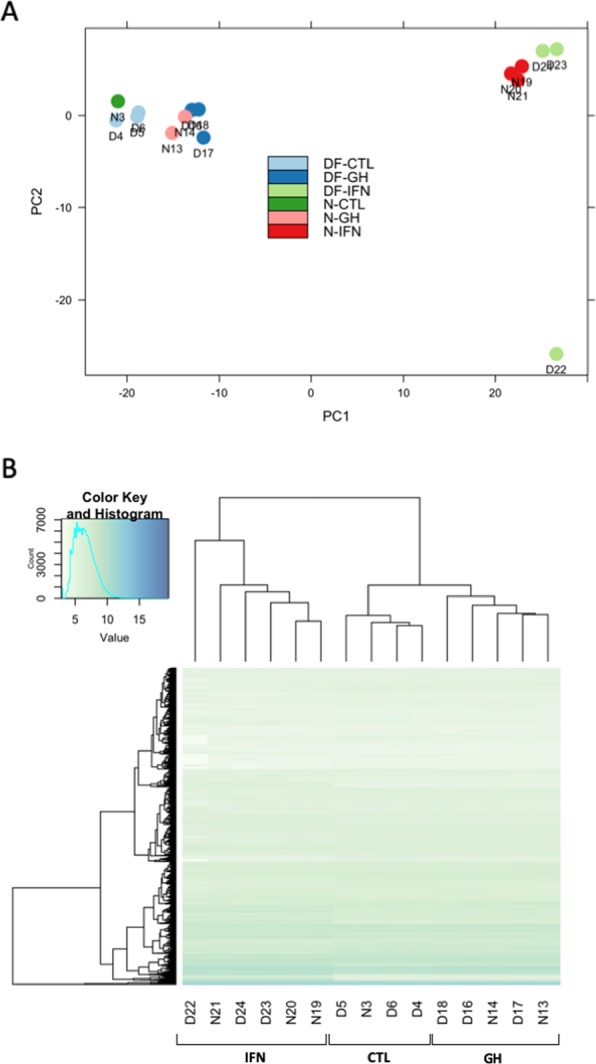
Principal components analysis of the 500 most variable expressed mRNAs in macrophages of Ames dwarf (D) and wild-type mice (WT) in the control, GH and IFN treatments (Panel A). Unsupervised hierarchical clustering of expression levels for the top 200 most expressed genes in macrophages of Ames dwarf (D) and Wild-type mice (WT) in the control, GH and IFN treatments (Panel B).

As a result of the RNASeq technique on average 20,850,687 reads per library were obtained. Two samples were removed due to very low transcript numbers after sequencing and pre-processing (<3,000,000). Using PCA (Fig. 1A) and unsupervised hierarchical clustering for all genes detected (Fig. 1B), we observed that samples grouped in similar pattern by treatment and showed a very low level of sample variability between genotypes. Only one sample was removed from the study due to an anomalous profile of gene expression (from GH treated cells). The grouping pattern indicate that samples are more affected by the *in vitro* treatment than the genotype of the mice.

**Figure 2:**
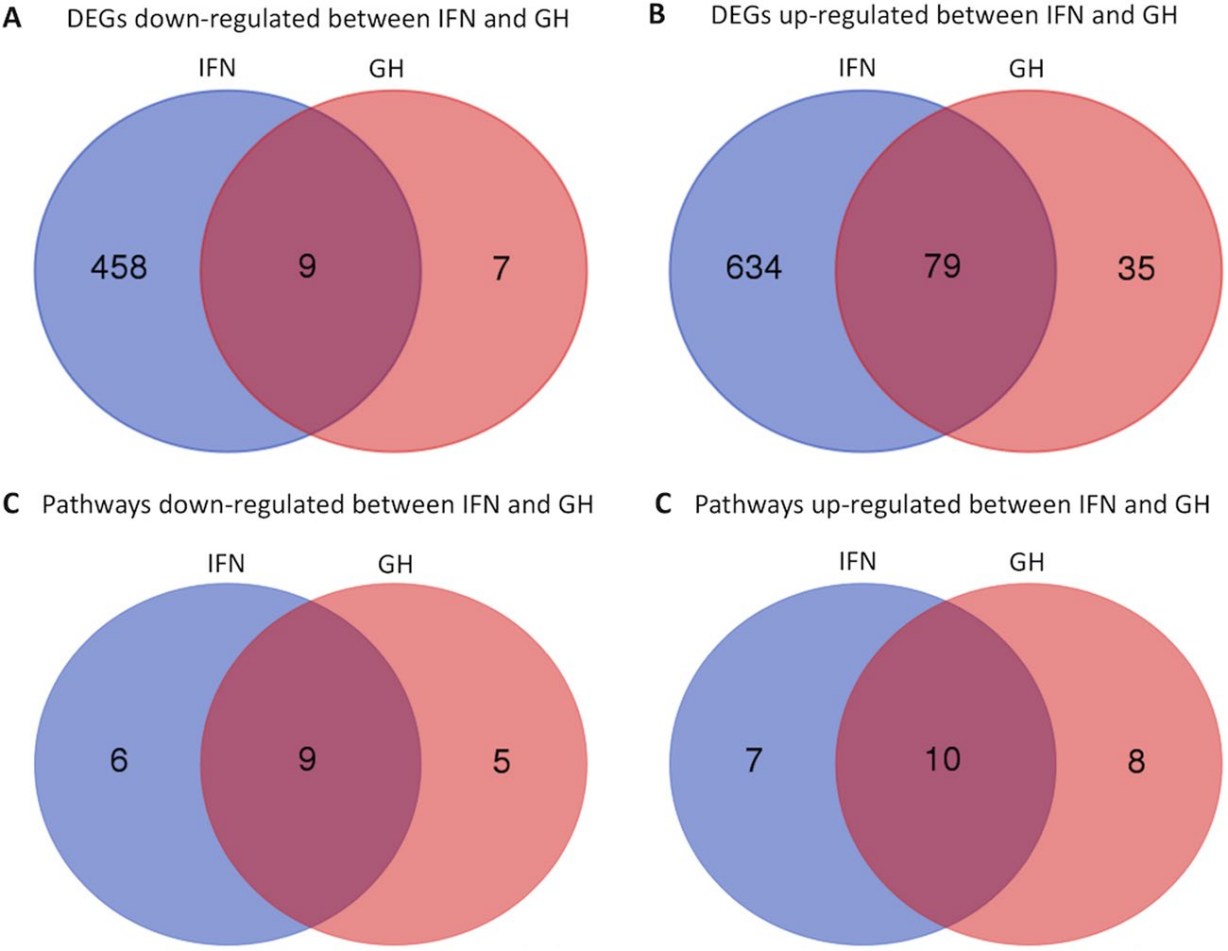
Number of differentially expressed genes (DEGs) down-regulated (**A**) or up-regulated (**B**) between GH and IFN treatments. Number of regulated Kegg pathways down-regulated (**A**) or up-regulated (**B**) between GH and IFN treatments.

**Figure 3:**
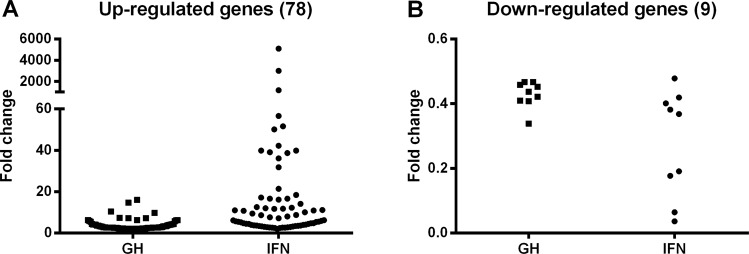
Fold change of expression for common down-regulated (**A**) or up-regulated (**B**) genes between GH and IFN treatments.

For analysis of differential gene expression (DEG) we considered initially the genes that were regulated with genotype (WT vs df/df) for control, GH or IFNγ treated cells. Since there was no gene regulation between genotypes at FDR < 0.02 and FC > 2.0 or FC < 0.5 for each treatment, we only considered genes that changed between treatments (Control *vs*. GH and Control *vs*. IFN) independently of genotype. We observed nine genes commonly down-regulated between GH and IFNγ treated cells, 458 genes exclusively down-regulated by IFNγ and 7 genes exclusively down-regulated in GH treated cells (Fig. 2A). Additionally, we observed 79 genes up-regulated by both treatments, 634 exclusively by IFNγ and 35 exclusively by GH (Fig. 2B). The FC distribution of genes commonly regulated in GH and IFNγ treated cells indicate stronger regulation (P < 0.05) for IFNγ (median of 8.7 and 0.44 for up and down-regulated genes, respectively) than GH (median of 2.7 and 0.36 for up and down-regulated genes, respectively) treated cells (Fig. 3). A detailed list with all the DEGs is provided in Suppl. Table 1. It is important to mention that there were no detectable levels of expression for the *Ifn* gene in any of the treatments. The top ten common up-regulated genes were *Nos2*, *Iigp1*, *Gbp5*, *Gbp2*, *Cxcl10*, *Gbp6*, *Gbp3*, *Gbp7*, *Cd40* and *Gm12250*. The enrichment for genes linked to macrophage activation induced by both IFNγ and GH, including inducible nitric oxide synthase and multiple guanylate-binding proteins, is notable. The top ten common down-regulated genes were *Eif4a2*, *Dab2*, *Rasgrp3*, *1810011H11Rik*, *Dhrs3*, *Rnf150*, *St6gal1*, *Snhg5*, *Slco4a1* and *Cxcr4*.

Considering the genes disturbed by the treatment using GAGE pathway analysis we identified nine commonly down-regulated Kegg pathways, six exclusively in IFNγ and five exclusively in GH treated cells (Fig. 2C). There were 10 Kegg pathways commonly up-regulated between GH and IFNγ treated cells, seven exclusively in IFNγ and eight exclusively in GH treated cells (Fig. 2D). The regulated Kegg pathways for each treatment are presented with details in Table 1. Additionally, we provided detailed figures of regulated genes in each selected pathway in Suppl. Figs. 2–4 for the Jak-Stat signaling, TLR signaling and cell cycle pathways for GH and IFNγ treated cells.

**Table 1:** Regulated pathways between GH and IFN treated macrophages.

Kegg Pathways	GH		IFN		Set size
	Stat Mean	P-Value	Stat Mean	P-Value	
**Common up-regulated pathways**					
mmu04621 NOD-like receptor signaling pathway	3.54	0.0003	1.92	0.0294	47
mmu04622 RIG-I-like receptor signaling pathway	3.23	0.0009	2.91	0.0024	45
mmu04620 Toll-like receptor signaling pathway	3.17	0.0010	2.40	0.0091	73
mmu04630 Jak-STAT signaling pathway	2.69	0.0040	4.03	0.0000	75
mmu04380 Osteoclast differentiation	2.23	0.0136	2.64	0.0045	92
mmu04623 Cytosolic DNA-sensing pathway	2.22	0.0153	2.59	0.0062	36
mmu04210 Apoptosis	2.13	0.0176	2.48	0.0071	71
mmu04510 Focal adhesion	2.03	0.0220	2.43	0.0079	113
mmu04666 Fc gamma R-mediated phagocytosis	1.86	0.0327	2.19	0.0151	72
mmu04514 Cell adhesion molecules (CAMs)	1.77	0.0404	2.64	0.0051	52
**IFN up-regulated pathways**					
mmu04145 Phagosome	—	—	2.89	0.0021	104
mmu04612 Antigen processing and presentation	—	—	2.93	0.0022	42
mmu04650 Natural killer cell mediated cytotoxicity	—	—	1.98	0.0249	71
mmu00760 Nicotinate and nicotinamide metabolism	—	—	2.01	0.0273	17
mmu04512 ECM-receptor interaction	—	—	1.82	0.0373	28
mmu04912 GnRH signaling pathway	—	—	1.70	0.0457	58
mmu04973 Carbohydrate digestion and absorption	—	—	1.70	0.0485	21
**GH up-regulated pathways**					
mmu04670 Leukocyte transendothelial migration	2.65	0.0045	—	—	62
mmu04920 Adipocytokine signaling pathway	2.21	0.0152	—	—	46
mmu04722 Neurotrophin signaling pathway	2.12	0.0177	—	—	104
mmu04660 T cell receptor signaling pathway	2.04	0.0217	—	—	73
mmu04062 Chemokine signaling pathway	1.88	0.0310	—	—	114
mmu04662 B cell receptor signaling pathway	1.76	0.0405	—	—	64
mmu04664 Fc epsilon RI signaling pathway	1.72	0.0440	—	—	52
mmu04520 Adherens junction	1.68	0.0486	—	—	49
**Common down-regulated pathways**					
mmu00190 Oxidative phosphorylation	−5.80	0.0000	−2.32	0.0108	98
mmu03030 DNA replication	−4.64	0.0000	−2.07	0.0219	35
mmu03010 Ribosome	−5.89	0.0000	−1.68	0.0483	80
mmu03430 Mismatch repair	−4.06	0.0001	−2.14	0.0200	22
mmu04110 Cell cycle	−3.18	0.0008	−3.31	0.0005	113
mmu03420 Nucleotide excision repair	−2.61	0.0054	−1.78	0.0398	42
mmu03040 Spliceosome	−2.53	0.0061	−1.72	0.0439	115
mmu03440 Homologous recombination	−2.42	0.0097	−1.82	0.0384	27
mmu00982 Drug metabolism - cytochrome P450	−1.90	0.0338	−1.85	0.0391	16
**IFN down-regulated pathways**					
mmu00280 Valine, leucine and isoleucine degradation	—	—	−1.98	0.0261	38
mmu00100 Steroid biosynthesis	—	—	−2.00	0.0281	14
mmu04710 Circadian rhythm - mammal	—	—	−1.93	0.0313	19
mmu03008 Ribosome biogenesis in eukaryotes	—	—	−1.87	0.0318	68
mmu04114 Oocyte meiosis	—	—	−1.82	0.0353	84
mmu00650 Butanoate metabolism	—	—	−1.80	0.0431	14
**GH down-regulated pathways**					
mmu03410 Base excision repair	−3.49	0.0005	—	—	30
mmu04260 Cardiac muscle contraction	−2.69	0.0046	—	—	32
mmu00240 Pyrimidine metabolism	−2.30	0.0114	—	—	81
mmu00230 Purine metabolism	−1.90	0.0291	—	—	116
mmu00980 Metabolism of xenobiotics by cytochrome P450	−1.95	0.0308	—	—	16

The number of enriched GO Terms for biological processes commonly up-regulated between GH and IFNγ treated cell was 261 (top ten: defense response, response to other organism, response to biotic stimulus, innate immune response, immune response, multi-organism process, immune effector process, response to bacterium, cellular response to interferon-beta, defense response to virus), with 277 exclusively up-regulated in IFNγ and 96 exclusively up-regulated in GH treated cells (top ten: macromolecule catabolic process, actin filament-based process, negative regulation of protein transport, modification-dependent macromolecule catabolic process, myeloid cell activation involved in immune response, negative regulation of cysteine-type endopeptidase activity, response to unfolded protein, membrane protein ectodomain proteolysis, response to hormone stimulus, vasoconstriction). There were 50 GO Terms for biological processes commonly down-regulated in IFNγ and GH treated cells (top 10: M phase, cell cycle phase, DNA repair, nuclear division, mitosis, M phase of mitotic cell cycle, DNA replication, organelle fission, cell division, mitotic cell cycle), 54 exclusively in IFNγ and 32 exclusively in GH treated cells (top 10: base-excision repair, proton transport, DNA integrity checkpoint, hydrogen transport, purine ribonucleoside biosynthetic process, purine nucleoside biosynthetic process, DNA damage checkpoint, ribosomal small subunit biogenesis, electron transport chain, G2/M transition of mitotic cell cycle). The enriched GO Terms for biological processes are presented with details in Suppl. Tables 3 and 4 for GH and IFNγ treated cells, respectively.

### Confirmation of sequencing results by real time PCR

The validation of RNASeq of ten of the top up-regulated genes (*Nos2, Gbp2, Gbp3, Gbp5, Gbp7, Lipg1, Igr1, Cxcl10, CD40, and Tnf)* by real time qRT-PCR confirmed our RNA-Seq findings and is presented in Suppl. Fig. 5.

### Reactive oxygen species production

**Figure 4:**
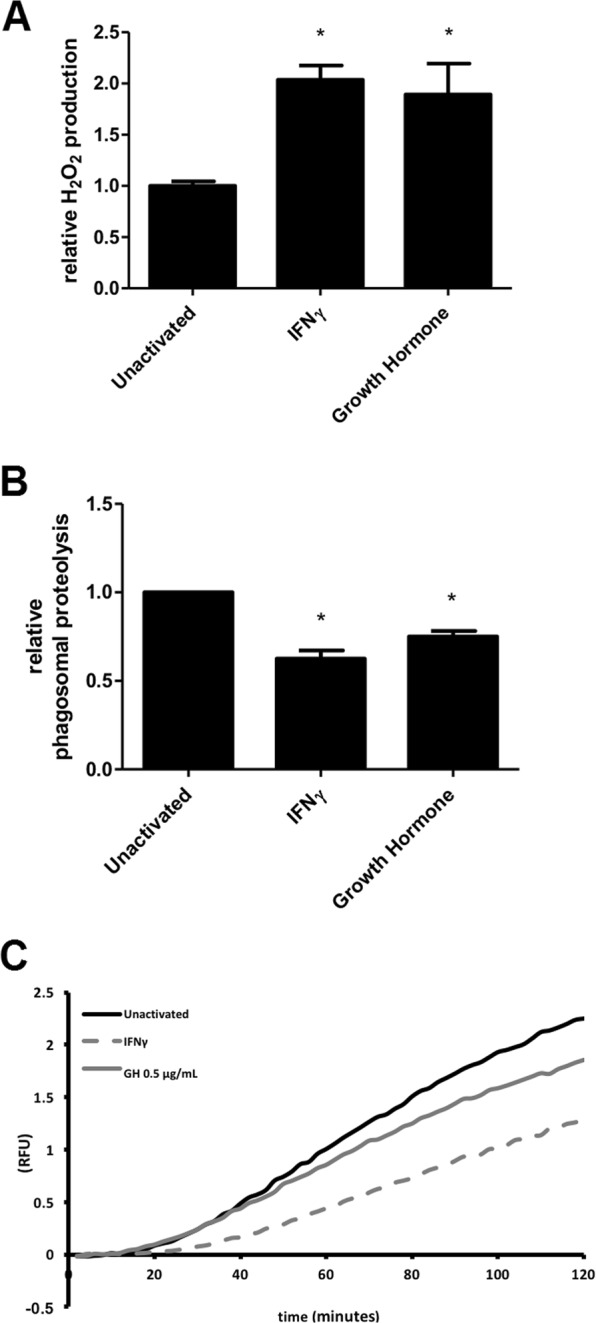
Effect of GH on functional properties of macrophages. For data shown in all panels, BMMØs from C57BL/6 mice were incubated for 18 hours in the presence of either 100 U/mL of IFNγ or 500 ng/mL porcine growth hormone. (**A**) Cells were then stimulated for 1.5 hours with serum-opsonized zymosan particles. Relative amounts of H202 within supernatants were determined by measuring the reaction of Amplex Red in the presence of horseradish peroxidase. Hydrogen peroxide production is presented as relative to unactivated BMMØs from 3 independent experiments. Error bars denote SEM. *p < 0.05. (**B**) Phagosomal proteolysis was measured as fluorescence liberation of DQ-Green BSA from IgG opsonized experimental particles that have been phagocytosed by unactivated/IFNγ/growth hormone activated BMMØs. Averaged rate of phagosomal proteolysis relative to unactivated control from 5 independent experiments. Rate was calculated between 80–100 minutes post phagocytosis. Error bars denote SEM. *p < 0.05. (**C**) Real time representative traces of phagosomal proteolysis.

Next, we set out to examine whether GH induced transcriptional changes measurably impacted macrophage functions such as antimicrobial effector mechanisms. The GH-mediated induction of multiple guanylate-binding proteins (Gbp), in particular Gpb7 which is known to enhance the oxidative burst through recruitment of NADPH oxidase to the phagosome^26^ prompted us to assess whether GH triggered production of reactive oxygen species. Consistent with this hypothesis, after exposure of macrophages to GH for 18 hours, we noted significantly increased H_2_O_2_ production to levels comparable with IFNγ treatment (P < 0.05) (Fig. 4A).

### Phagosomal proteolysis

Based on our previous report that NADPH oxidase (NOX2) negatively regulates phagosomal proteolysis^27^ we measured the the proteolysis of a quenched fluorescent protein substrate within phagosomes of unstimulated macrophages or macrophages stimulated with either GH or IFNγ. Similar to macrophages treated with IFNγ, those treated with GH displayed lower levels of phagosomal proteolysis compared to unactivated samples (P < 0.05) (Fig. 4B,C).

## Discussion

In this study, global transcriptomic analyses corroborated by macrophage functional assays provided detailed insight into GH mediated priming of macrophages. We noted a significant overlap between pathways activated by GH and IFNγ, although the total number of genes activated by IFNγ treatment was much higher. If we consider the 50 highest induced genes in each treatment, we can observe 21 common genes among them. The higher fold-change induction of these shared genes in IFNγ versus GH treated cells suggests a stronger effect of IFNγ compared to GH. This is in agreement with previous suggestions that the dose of GH required to prime macrophages is higher than that of IFNγ^22^. This does not necessarily minimize the physiological relevance of GH as IFNγ levels in healthy mice are very low (0 to 15 pg/mL)^28^ whereas GH is present at higher levels (4 to 25 ng/mL)^29^. It is important to mention that previous studies showed that blocking of the IFNγ receptor did not block the effects of GH^22^, suggesting that GH effects are independent of IFNγ produced by possible bystander lymphoid cells. We did not detect expression of the *Ifn* gene in macrophages from any treatment, in agreement with previously observations at the protein level for unstimulated macrophages^30^. Therefore, we can exclude that the observations could be due to GH-induced IFNγ production. On the other hand, it should be considered that cells were cultured in the presence of 10% fetal calf serum, which may contain both GH and cytokines. However, as cells from both groups were cultivated in the presence of serum, we understand it has no impact on the identification of differentially regulated genes among groups.

The top regulated biological terms for both GH and IFNγ treated cells include response to bacterium, response to virus, response to other organism, defense response and phagocytosis, consistent with a role for GH in immune responses to infections. Additionally, terms like biological adhesion and cell adhesion were also up-regulated in GH and IFNγ treated cells, supporting a role for GH in regulating the ability of monocytes to adhere to cells in the presence of IFNγ^31^. Consistent with our observation that cell migration was also one of the up-regulated GO Terms, severe reduction of macrophage infiltration into specific tissues, in df/df mice has been reported^32^. Furthermore, a defective expression of genes in the myeloid cell differentiation pathway was demonstrated in peripheral leukocytes of df/df mice compared to N mice^33^, further suggesting this inability of monocytes to migrate to tissues in the absence of GH. Conversely, Kumar and Menon^34^ reported that *in vivo* GH treatment also enhances monocyte migration to tissue, which can improve the ability to fight infection in local tissues. This evidence, combined with our current findings, indicated that GH may exert a significant impact on the maturation, activation, and functionality of effector macrophages.

Global RNAseq profiling of GH- and IFNγ- induced genes also highlighted several biological terms for pattern recognition and activation of the immune response among the most enriched terms in both treatments. Toll-like receptors (TLRs) receive signals from the extracellular space, while members of the RIG-I-like receptors (RLRs) and NOD-like receptor (NLR) families are sensors of infection and stress in the intracellular space^35^. These pathways are involved in the response to endogenous molecules released by host cells resulting from necrosis or damage^36^. These three pathways were highly up-regulated in both GH and IFNγ treated cells. The activation of signaling pathways via TLRs, RLRs and NLRs culminate in the activation of nuclear factor κB (NF-κB)^36^, which is essential for propagation of the inflammatory response, release of pro-inflammatory cytokines and expression of adhesion molecules^37^. We detected several biological terms referring to increase release of inflammatory cytokines up-regulated for both GH and IFNγ treated cells, including cytokine production, regulation of cytokine production, positive regulation of cytokine production, cytokine secretion, positive regulation of cytokine secretion, regulation of cytokine production involved in immune response and cytokine biosynthetic process. This suggests that GH, like IFNγ, enhances pattern recognition and pro-inflammatory processes that are critical to the innate immunity role of macrophages. More studies assessing the effect of GH on the response of cells to LPS or stimuli are necessary to further elucidate this response.

No differences in the gene expression profile were observed between macrophages derived from bone marrows of WT or GH-deficient df/df mice. This finding is very interesting and suggests that, after extraction of bone marrow precursors and differentiation into macrophages *in vitro*, the GH-deficient bone marrow environment in which they developed does not affect their profile at all. As mentioned before, GH-deficient Snell dwarf mice, despite having absent GH pituitary secretion, do have expression of GH in bone marrow cells^12^. Although there is still controversy about the functionality of the immune system of df/df mice, it is well established that df/df mice have an anti-inflammatory profile when compared to WT mice, with reduced secretion of pro-inflammatory cytokines^33^ and reduced macrophage infiltration in tissues^10^. Nevertheless, it has been shown that df/df mice have increased myelopoeisis in the bone marrow^38^. Additionally, some reports indicate that df/df mice have a deficient immune response, which can be restored by GH treatment, but not by treatment with bone marrow cells from a normal mice^39^. This suggests that the presence of GH is essential for the maturation of these cells and bone marrow cells from normal mice do not retain the ability to mature in the absence of the pituitary hormones. Future studies to assess the functionality of tissue macrophages from df/df mice will be important to fully elucidate the impact of GH on these key cells of the innate immune system.

The overlap between GH- and IFNγ-dependent genes can be attributed, at least in part, to intersecting downstream signaling pathways. GH binds to the growth hormone receptor (GHR) in the cell surface, which is a transmembrane protein of the cytokine receptor family, the same receptor family as many interleukin receptors^3,4^. Activation of GHR leads to intracellular activation of the Jak-STAT and transduction of GH signals^40^. IFNγ also binds to its receptor in the cell surface activating mainly the Jak-STAT signaling pathway^41^. The Jak-STAT pathway in the macrophage is critical to allow its activation and adequate immune response^42^. In our study, the Jak-STAT pathway was among the highest up-regulated pathway by both GH and IFNγ. One important target of STATs is the *Nos2* gene^43^, which in our study was the top-regulated gene for both treatments (64 and 467-fold up-regulated by GH and IFNγ, respectively). *Nos2* encodes the inducible nitric oxide synthase (iNOS) which is directly responsible for production of reactive nitrogen species (RNS) that exert potent antimicrobial effects on many pathogens, including *Mycobacterium tuberculosis*^44–47^. Corroborating with this, among the common up-regulated GO terms between IFNγ and GH were regulation of nitric oxide biosynthetic pathways, nitric oxide metabolic process and positive regulation of nitric oxide biosynthetic pathways.

In addition to polarizing macrophages toward a pro-inflammatory state, IFNγ also alters the lumenal contents and functional capacity of their phagosomes which serves to optimize their response to pathogens^27,48–51^. One of the major changes to the phagosomal environment following IFNγ stimulation is the increase in ROS through increased expression and activation of the phagocyte NADPH oxidase 2^52^. Increased ROS production by the macrophage constitutes an important effector mechanism for bacterial killing^53,54^ and is an important marker of macrophage activation^55^. To complement our global transcriptomics view of macrophage priming by GH, we also demonstrated that GH triggered increased ROS production comparable to IFNγ treatment, despite the subtler GH-induced changes in gene expression. This corroborates earlier observations in a rat model showing enhanced macrophage ROS production and antimicrobial activity against *Pasteurella* and *Salmonella* both *in vitro* and *in vivo*^22,23^. Additionally, among the GO Terms up-regulated for both GH and IFNγ treated macrophages was regulation of oxidoreductase activity, which is involved in the release of ROS and RNS. In addition to directly killing phagocytosed pathogens, the long half-life and membrane permeability of H_2_O_2_ enables it to also act as a secondary messenger. Thus, the induction of ROS production by GH may impact numerous other processes including phagocytosis, cell cycle and cell death, monocyte recruitment, and monocyte-to-macrophage differentiation^56^.

In addition to the production of ROS, activation of macrophages with IFNγ has also been reported to modify the degradative capacity of the phagosome, particularly the degradation of proteins^51^. Our findings revealed that GH stimulation of macrophages reduced phagosomal proteolysis to a similar extent as IFNγ, highlighting a novel facet of GH-mediated macrophage priming and activation. It is known that IFNγ limits the efficiency of phagosomal proteolysis in part through oxidative inhibition of cysteine proteases, and can result in modified processing of MHC-II-restricted antigens^27,57^. The mechanism by which GH dampens the proteolytic capacity of macrophage phagosomes and the effect on antigen presentation remain to be determined.

Although many lines of evidence point to an important role for GH in macrophage priming and innate immunity, the relevance of GH and changes in GH levels (i.e. during aging) to immune senescence and specific susceptibilities remains unclear. On the one hand, studies in humans with congenital GH deficiency indicate no difference in the incidence of several viral and bacterial infections as well as response to vaccination^58^. On the other hand, reduced macrophage function is evident in older individuals and is associated with increased susceptibility to infections^59^. Absence of GHR signaling can prevent activation of the NLPR3 inflammasome in macrophages, reducing the inflammatory response to endogenous ligands from cellular necrosis^60^. Some reports also suggest that GH drives macrophages through a M2 anti-inflammatory phenotype characterized by low Nos2 and high arginase-1 expression^61^. However, our data indicate high levels of Nos2 and no changes in arginase-1 expression for both GH and IFN treatments, suggesting an M1 pro-inflammatory phenotype. Our data strongly support a potential link between GH, which wanes with age, and impaired macrophage function. This study has provided the first global view of the scope of GH-mediated changes in macrophage gene expression. The notable overlap with IFNγ-induced pathways involved in innate immune sensing of pathogens and antimicrobial responses argue for an important role for GH in macrophage priming and maturation. Additional studies will be required to confirm that these effects observed with bone marrow-derived macrophages are generalizable to specific differentiated macrophage subtypes. By using functional assays that report on biochemical activities within the lumen of phagosomes, we have shown that GH alters physiologically relevant processes such as ROS production and proteolysis. These changes could have far reaching impacts on antimicrobial capacity, signaling, and antigen presentation. Additional work beyond the scope of this study is required to fully characterize the role of GH on innate immunity and host reponses to infection.

## Methods

### Tissue collection and macrophage culture

All animal procedures employed in our present work were approved by and performed in accordance with the guidelines from the Laboratory Animal Care and Use Committee at the University of Central Florida or Animal Care and Use Committee at the University of Calgary. A total of six mice were used for this study. Wild-type (WT; n = 3) and Ames dwarf mice (df/df; n = 3) were bred and maintained under temperature- and light-controlled conditions (22 ± 2 °C, 12 hour light/12 hour dark cycle)^62^. The animals were anesthetized and euthanized for collection of femur samples for bone marrow extraction.

Bone-marrow derived macrophages (BMMØs), were derived from bone marrow isolated from the femurs, tibias and ilia of mice and cultured in DMEM supplemented with 10% fetal calf serum, 1% L-glutamine, 1% sodium pyruvate, 1% penicillin/streptomycin and 20% L-cell conditioned media from M-CSF producing L929 cells. BMMØs were maintained at 37 °C and 7% CO_2_, and were considered fully differentiated ten days after bone marrow isolation. This protocol yields >95% CD11b + F4/80+ macrophages^63,64^.

Fully-differentiated macrophages (from 3 WT and 3 df/df mice) were cultured in macrophage media as above, incubated at 37 °C and 5% CO_2_ divided into four treatments (samples of each mice were represented in each treatment): 1) Control (no GH and no IFN), 2) 50 ng/mL of recombinant porcine GH (Reporcin, Alpharma, Inc., Victoria, Australia), 3) 500 ng/mL of GH (Reporcin), and 4) 50 U/mL of IFNγ (Peprotech, Canada). Samples from each mouse/treatment (n = 48 samples) were collected for RNA extraction at 6 h and 24 h after the beginning of treatments. The doses of GH tested were based on previous studies^25,65,66^ and pilot experiments.

### RNA extraction and real time PCR

The macrophages were removed from culture and homogenized with 3 mL of Qiazol (Qiagen, Valencia, CA, USA) and gDNA Eliminator (RNeasy Mini Kit, Qiagen). Next, chloroform was added, and the samples were homogenized and incubated for 3 min at room temperature. Samples were then centrifuged at 4 °C at 12,000 xg for 15 min and the clear upper phase transferred to a new tube with cold ethanol. The solution was transferred to columns (RNeasy Mini Kit, Qiagen) following manufacturer’s instructions. The quantity of RNA was determined using a spectrophotometer (Epoch Microplate Spectrophotometer, Biotek, Winooski, VT, USA). Reverse transcription reactions were performed with 1 μg of RNA (5 μL) using iScript Synthesis Kit (Biorad, Hercules, CA, USA) in a 20 μL volume incubated for 5 min at 25 °C, 20 min at 46 °C and 1 min at 95 °C (MJ Mini Personal Thermal Cycler, Biorad). The final cDNA solution was diluted to 10 ng/μL before use.

Real-time PCR using SYBR Green dye was used to evaluate gene expression. β2 microglobulin (*B2m*) expression was used as an internal control and *Tnf* as an initial target gene for assay validation and sample selection, based on previous reports of GH-mediated *Tnf* induction^24^. The primer sequences are listed in Table 2 and included genes differentially regulated and used to validate the results of the RNASeq analysis. Validation of RNASeq analysis was performed with a group samples from nine N mice processed as previously described. The PCR reactions were performed in duplicate in a 20 μL volume using 5 μL of Fast SYBR Green Mastermix (Applied Biosystems, Foster City, CA, USA), 0.4 μL of each primer (10 μM stock) and 20 ng of cDNA. Fluorescence was quantified with the ABI Prism 7500 Fast Real Time PCR System (Applied Biosystems). For each assay, 45 PCR cycles were run (95 °C for 3 sec and 60 °C for 30 sec) and a dissociation curve was included at the end of the reaction to verify the amplification of a single PCR product. Analyses of amplification plots were performed with the 7500 Software (Applied Biosystems). Each assay plate included a negative control. Relative expression was calculated from the 2^−ΔΔCt^ equation^67^.

**Table 2:** Primer sequences used in the study.

Gene	Accession		Sequence 5′-3′	Product size (bp)
*Nos2*	NM_010927.4	Forward	GTGAAAAGTCCAGCCGCACC	206
		Reverse	CCAGTAGCTGCCGCTCTCAT	
*Tnf*	NM_013693.3	Forward	CCACGCTCTTCTGTCTACTG	145
		Reverse	GCTACAGGCTTGTCACTCG	
*Iipg1*	NM_001146275.1	Forward	GACACAGGAGTTTCTGTGCCTTT	102
		Reverse	ACCAGTAAAGCTGGAGGGCA	
*Cxcl10*	NM_021274.2	Forward	CCACGTGTTGAGATCATTGCCA	144
		Reverse	TGCGTGGCTTCACTCCAGTT	
*Igr1*	NM_008392.1	Forward	ACCAAAGAGATTCCACCCTCCC	153
		Reverse	TGAGTGGCAGCGTTCGCTAT	
*Cd40*	NM_011611.2	Forward	CCCTGGACAAGCTGTGAGGA	147
		Reverse	CACCCCGAAAATGGTGATGAGG	
*Gbp2*	NM_010260.1	Forward	CCAGCTGCACTATGTGACG	160
		Reverse	GGGTTTTCCGTTAACCTCCAG	
*Gbp3*	NM_001289492.1	Forward	TAGTGTTCCCTGACGCTGCC	113
		Reverse	GCCACAAGACCCTGTGAGGT	
*Gbp5*	NM_153564.2	Forward	TTTTGACGCTCCTGCGCTTG	177
		Reverse	AGGCTTTCTAGACGAGGTCCG	
*Gbp7*	NM_013506.3	Forward	TTGTGGCTTCCGAAAGGGGA	169
		Reverse	GCCGCCATGTTCTCTGTTGTAA	
*B2m*	NM_009735.3	Forward	AAGTATACTCACGCCACCCA	217
		Reverse	CAGCGCTATGTATCAGTCTC	

### RNA sequencing analyses

Transcriptomic profile of individual samples was performed using commercial RNA-sequencing kits (NEBNext mRNA Library Prep Master Mix and NEBNext Multiplex Oligos for Illumina, New England Biolabs, Ipswich, MA, USA) and adapted according to previous descriptions^68,69^ using 5 µg of total RNA per sample as initial input. Only samples from the Control (n = 3 WT and n = 3 df/df), 500 ng/mL of GH (n = 3 WT and n = 3 df/df) and 10 ng/mL of IFN (n = 3 WT and n = 3 df/df) at 6 h time point were used for RNA-Seq. Briefly, Poly A RNA enrichment was performed using the Magnetic mRNA Isolation kit (New England Biolabs) according to manufacturer instructions. Subsequently, samples were processed using the NEBNext mRNA Library Prep Master Mix (New England Biolabs) with slight modifications from the manufacturer’s instructions. After the RNA fragmentation step, RNA samples were cleaned and concentrated using RNA Clean & Concentrator™-5 (Zymo Research, Irvine, CA, USA). After this initial step, first and second strand cDNA were generated, samples were subjected to end-repair, dA tailing and finally adaptor ligation. Between each step samples were cleaned up using the Qiagen MinElute kit (Qiagen). Fragments between 150–300 bp were recovered after 2% agarose gel electrophoresis (QIAquick Gel Extraction Kit, Qiagen) in order to remove adapter dimers by size selection. Nine different indexes (NEBNext Multiplex Oligos for Illumina, New England Biolabs) were added to individual libraries during 12 cycles of PCR amplification. After that, samples were combined in two mixtures (nine in each mix), and submitted to sequencing on two flowcell lanes on a HiSeq. 2500 instrument (Illumina Inc., San Diego, CA, USA). All RNA-Seq data are available at the Sequence Read Archive (SRA) at NCBI under accession number SRP142633.

The mapping of sequencing reads to the mouse transcriptome (Illumina iGenomes annotation for UCSC mm10, http://support.illumina.com/sequencing/sequencing_software/ igenome.html) was performed using Tophat 2.1.0.0 and Bowtie 2.2.6^70^. mRNA abundance was calculated using Cufflinks 2.1.1^71^ and is presented as Fragments Per Kb of exon per Million reads mapped to mRNAs (FPKM). The number of reads aligned to its corresponding gene was calculated by HTSeq. 0.6.1^72^. Genes with an average FPKM lower than 1/100,000th of the total aligned reads in more than 50% of the samples were eliminated from further analyses (10,117 transcripts selected with these criteria from 23,457 transcripts available in the mm10 database). mRNAs were further processed for pathway analysis using the Generally Applicable Gene-set Enrichment (GAGE), which uses log- based fold changes as per gene statistics, and Pathview packages in R^73,74^, using KEGG molecular pathways database^75,76^. Enrichment of gene ontology (GO) terms (biological processes, molecular function and cellular component) was also performed using the GAGE package using same criteria described for pathways analysis. P values lower than 0.05 were considered as significant for pathways and GO Terms analysis.

### Reactive oxygen species production

Macrophages derived from C57BL/6 mice as described above, were incubated for 18 hours in the presence of either 10 ng/mL of IFNγ or 500 ng/mL of porcine GH. Immediately prior to the addition of experimental particles, growth medium was removed from cells and replaced with assay medium with low autofluorescence (DPBS containing 1 mM CaCl_2_, 2.7 mM KCl, 0.5 mM MgCl_2_, 5 mM glucose, 0.1% calf skin gelatin, and 10 mM HEPES). Cells were stimulated for 1.5 hours with 50 μg/mL of serum-opsonized zymosan particles (Sigma Aldrich). Relative amounts of H_2_O_2_ within supernatants were determined by measuring the reaction of 20 μM Amplex Red (Thermo Fisher Scientific) in the presence of horse radish peroxidase using a FLUOstar Optima fluorescence plate reader (BMG Labtech) as previously described^48,77^. Hydrogen peroxide production is presented as relative to unactivated macrophages from 3 independent experiments.

### Phagosomal proteolysis

For *in vitro* phagosomal assessment, BMMØs from C57BL/6 mice were plated in 96 µClear® black microplates (Greiner Bio-One) 16 h prior to fluorometric analyses. The hydrolytic capacity of the phagosome was assessed using 3.0 µM silica experimental particles bearing the proteolytic substrate, DQ-Green BSA (Thermo Fisher) and a calibration fluor (Alexa Fluor 594 SE (succinimidyl ester)) in live macrophages as previously described^14,50,78^. Briefly, phagosomal proteolysis was determined by measuring the fluorescence liberated by the fluorogenic substrate DQ-Green BSA from IgG-opsonized experimental particles that had been phagocytosed by macrophages cultured in the presence of either 10 ng/mL of IFNγ or 500 ng/mL porcine GH for 18 hours prior to assessment. The average rates of phagosomal proteolysis were calculated as the average rate of increase in substrate fluorescence relative to the calibration fluorescence between 80–100 minutes post phagocytosis. Data is presented as relative to unactivated macrophage samples from 5 independent experiments.

### Statistical analyses

The results are presented as mean ± standard error of the mean (SEM) for real time PCR results and *in vitro* assessment of phagosomal characteristic. All statistical analyses for these parameters were performed using GraphPad Prism 5.0 (GraphPad Software Inc., La Jolla, CA, USA) using a one-way ANOVA with a Tukey post-test. P values lower than 0.05 were considered as significant.

Statistical analyses for differentially expressed mRNAs was performed using the software R (3.2.2) and the Bioconductor package DESeq (1.2.0)^79^ using the HTSeq output count. Read counts were normalized for library depth, and pairwise comparisons between genotypes and treatment, measuring fold change, uncorrected P-values from the negative binomial distribution, and adjusted P values (false discovery rate; FDR) were obtained. Principal components analysis (PCA) including all genes was also performed using R to observe sample distribution in a two-dimensional plot and eliminate outliers. Unsupervised hierarchical clustering, included all genes, was performed also using the DESeq package to observe sample clustering. Genes with a FDR <0.02 and fold change (FC) >2.0 were considered as up-regulated; and with FDR <0.02 and FC <0.5 were considered as down-regulated.

## Supplementary information

Suppl Figures and Tables

## Data Availability

All RNA-Seq data are available at the Sequence Read Archive (SRA) at NCBI under accession number SRP142633. Other datasets generated during and/or analyzed during the current study are available from the corresponding author on reasonable request.
