# Supplementary material for: Growth hormone-mediated reprogramming of macrophage transcriptome and effector functions
Source: Sci Rep. 2019 Dec 18;9:19348. doi: 10.1038/s41598-019-56017-6 (PMC6920138; doi:10.1038/s41598-019-56017-6)
Supplement: Supplementary file 1 — Suppl Figures and Tables [file 41598_2019_56017_MOESM1_ESM.pdf]

Growth hormone-mediated reprogramming of macrophage transcriptome and effector functions

Augusto Schneider[1,2], Hillary N. Wood[2], Sandra Geden[2], Catherine J. Greene[3], Robin M. Yates[3,4], Michal M. Masternak[2,5*], Kyle H. Rohde[2*]

[1]Faculdade de Nutrição, Universidade Federal de Pelotas, Pelotas, RS, Brazil
[2]College of Medicine, Burnett School of Biomedical Sciences, University of Central Florida, Orlando, FL 32827
[3]Department of Biochemistry and Molecular Biology, Cumming School of Medicine, University of Calgary, Calgary, Alberta, Canada
[4]Department of Comparative Biology and Experimental Medicine, Faculty of Veterinary Medicine, University of Calgary, Calgary, Alberta, Canada
[5]Department of Head and Neck Surgery, The Greater Poland Cancer Centre, Poznan, Poland

***Correspondence:**
Kyle H. Rohde (lead author) and Michal M. Masternak
E-mails: kyle.rohde@ucf.edu and michal.masternak@ucf.edu

Supplemental Figure

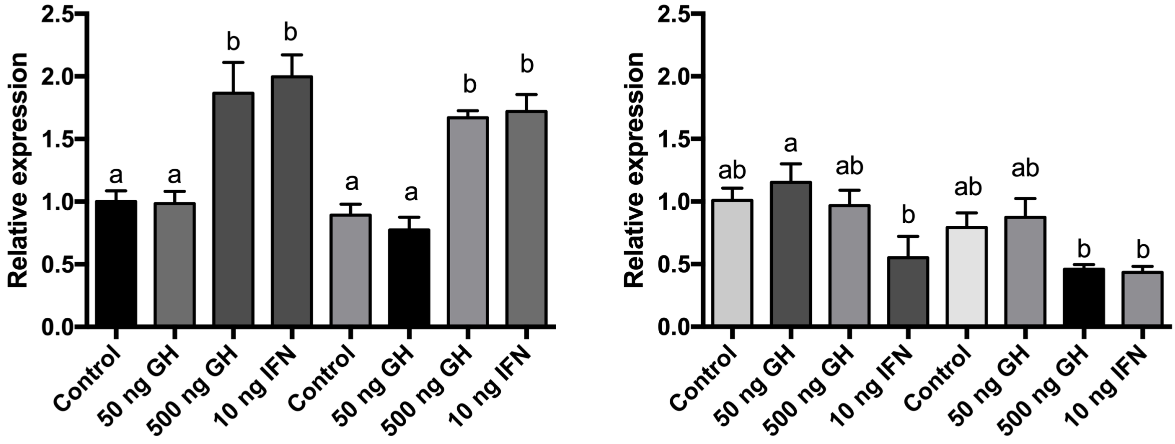

Suppl. Figure 1 – Tumor necrosis factor (*Tnf*) relative gene expression in control, GH and IFN treated macrophages from Ames dwarf and wild-type mice 6 or 24 hours after initial treatment. Different letters indicate statistical difference at P<0.05.

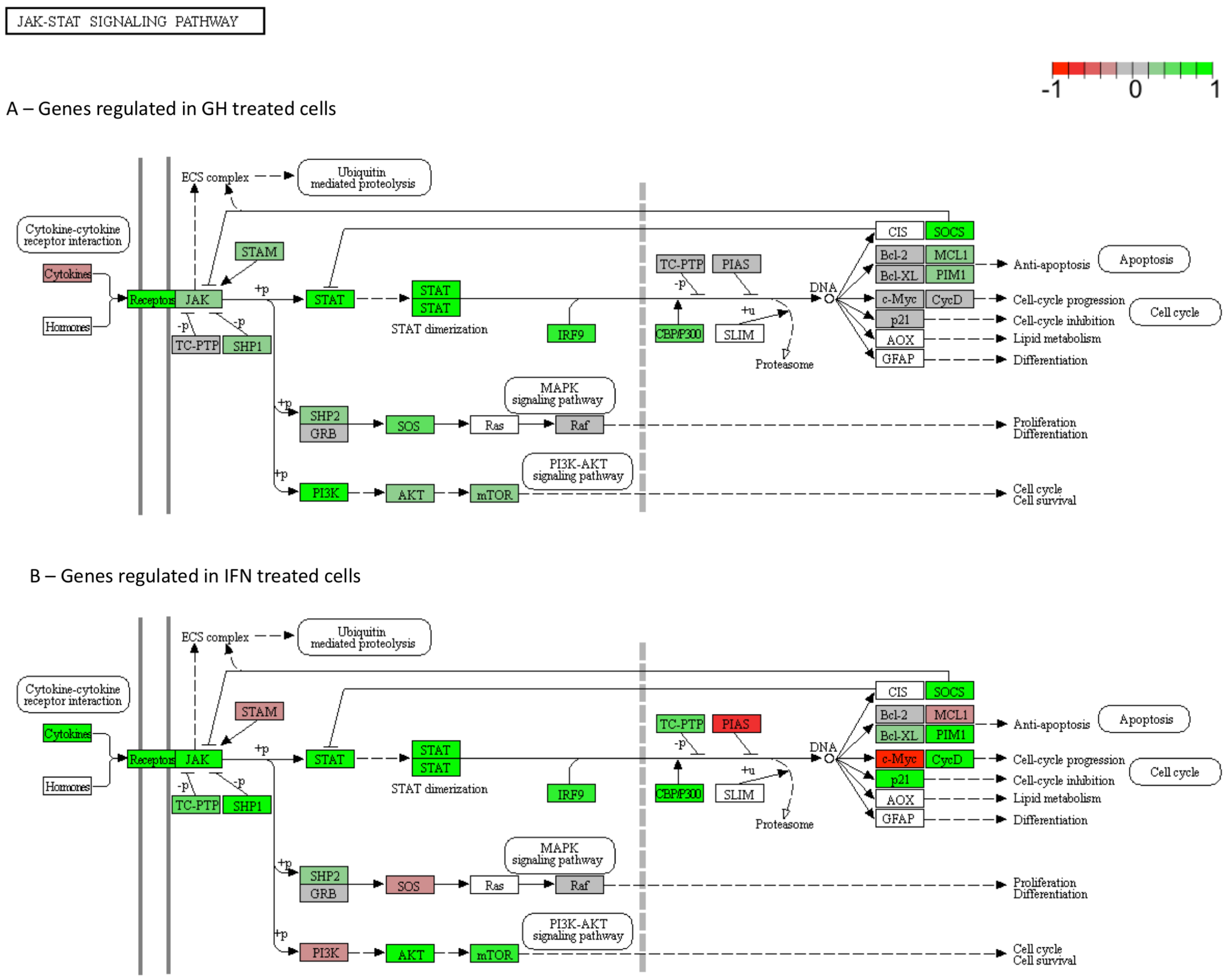

Suppl. Figure 2 – Schematic representation of the Jak-Stat signaling pathway and its regulated set of genes. The Jak-Stat signaling pathway was up-regulated in GH (Panel A) and IFN (Panel B) treated cells. Grey box – target gene not differentially regulated; Green box – target gene up-regulated; and Red box – target gene down-regulated. KEGG pathway map 04630 is adapted here from http://www.kegg.jp as described previously[75,76].

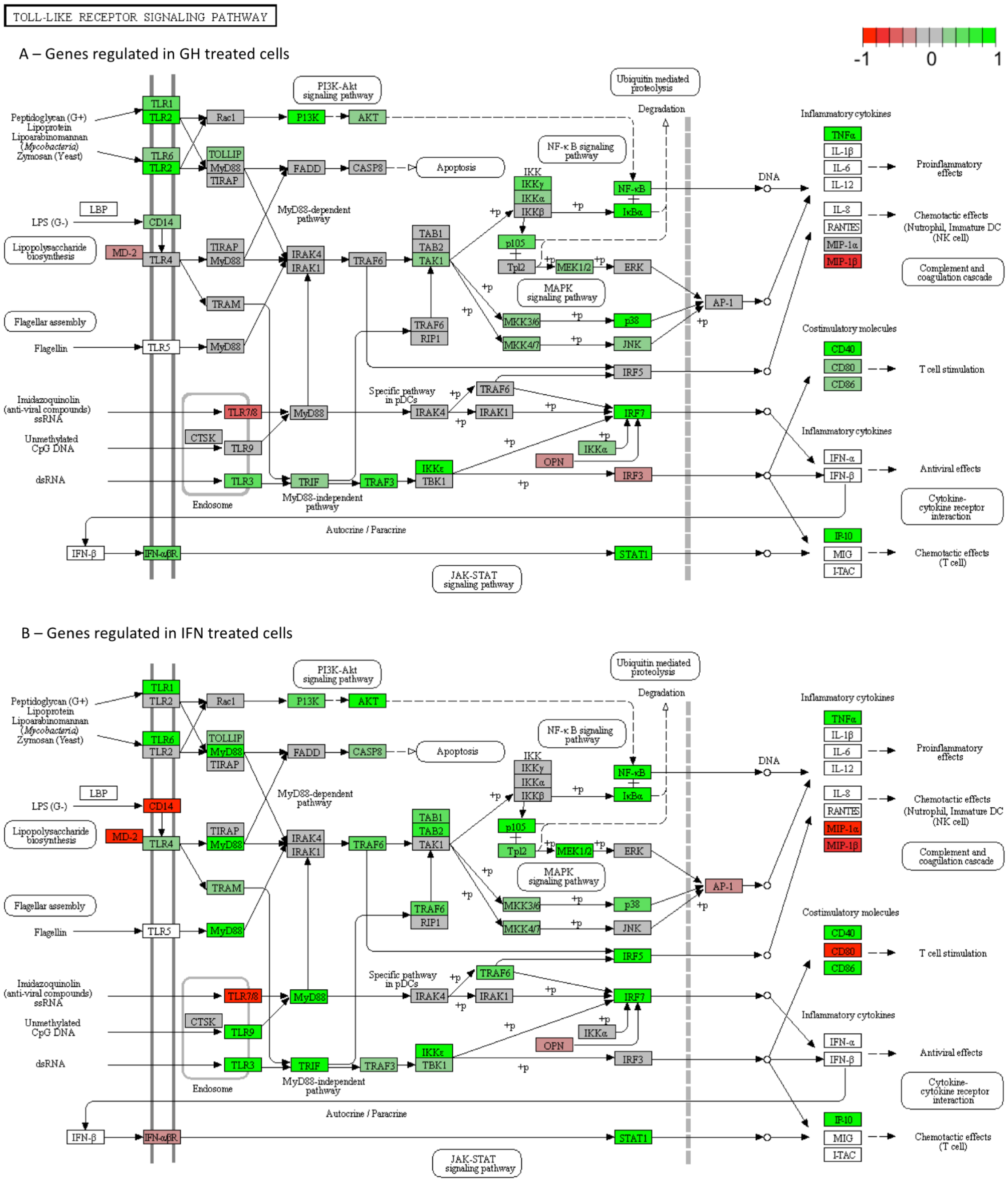

Suppl. Figure 3 – Schematic representation of the Toll-like receptor signaling pathway and its regulated set of genes. The Toll-like receptor signaling pathway was up-regulated in GH (Panel A) and IFN (Panel B) treated cells. Grey box – target gene not differentially regulated; Green box – target gene up-regulated; and Red box – target gene down-regulated. KEGG pathway map 04620 is adapted here from http://www.kegg.jp as described previously[75,76].

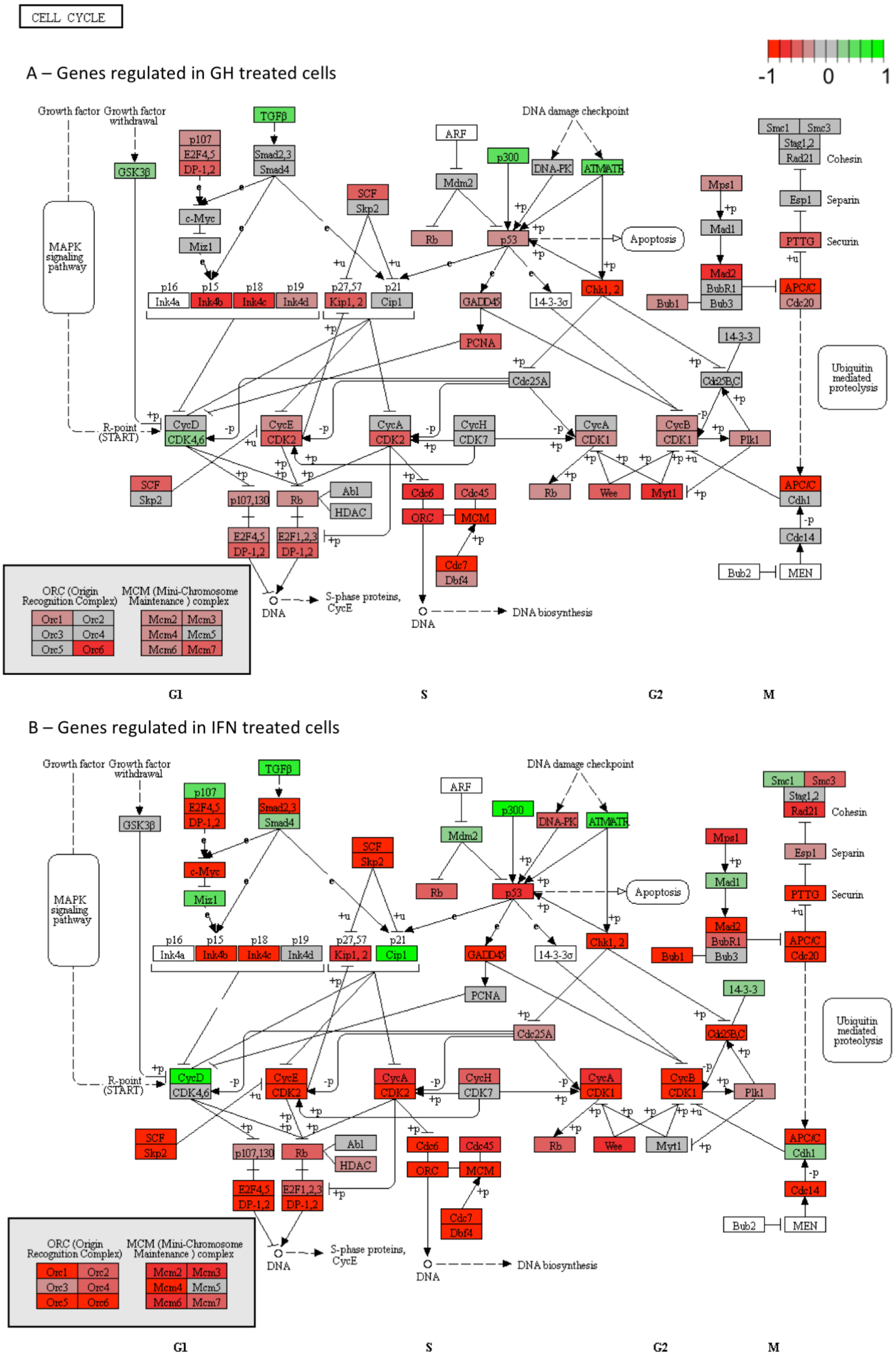

Suppl. Figure 4 – Schematic representation of the cell cycle pathway and its regulated set of genes. The cell cycle pathway was down-regulated in GH (Panel A) and IFN (Panel B) treated cells. Grey box – target gene not differentially regulated; Green box – target gene up-

regulated; and Red box – target gene down-regulated. KEGG pathway map 04110 is adapted here from http://www.kegg.jp as described previously[75,76]..

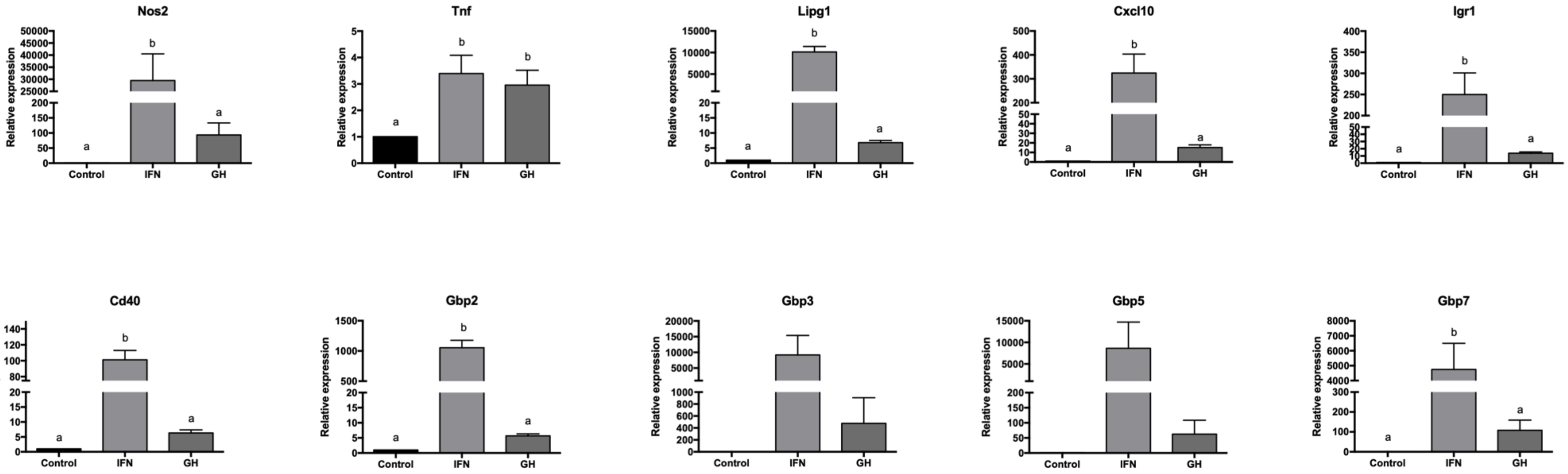

Suppl. Figure 5 – Relative gene expression in control (n=9), GH (n=9) and IFN(n=9) treated macrophages from Ames dwarf and wild-type mice 6 hours after initial treatment. Different letters indicate statistical difference at P<0.05.

Supplemental Table 1 – Genes regulated in GH vs Control

Gene	Control	GH	FC	P-Value	FDR
**Up-regulated**					
Nos2	0.09	5.91	64.26	4.07E-07	2.86E-05
Irg1	72.85	1164.10	15.98	8.08E-91	8.17E-87
Gbp5	1.57	23.06	14.68	7.97E-28	1.01E-24
Cxcl10	6.59	68.28	10.36	2.50E-18	1.41E-15
Mmp14	39.41	385.33	9.78	1.84E-71	9.33E-68
Ptges	8.58	62.89	7.33	4.48E-16	1.89E-13
Cd40	2.10	15.23	7.26	3.18E-09	3.78E-07
Gpr84	40.92	292.85	7.16	2.00E-33	4.05E-30
Rsad2	31.16	196.33	6.30	5.71E-30	9.63E-27
Iigp1	0.84	5.24	6.26	1.46E-07	1.15E-05
Gbp6	1.40	8.78	6.26	8.13E-07	5.31E-05
Gbp3	3.66	22.42	6.12	2.15E-15	8.05E-13
Socs3	4.10	24.14	5.89	1.24E-04	3.87E-03
Ifit1	20.42	116.79	5.72	1.73E-35	4.37E-32
Tnfsf15	1.56	8.50	5.44	1.12E-05	5.22E-04
H2-M2	6.95	37.04	5.33	1.64E-07	1.27E-05
Fpr2	12.33	58.34	4.73	1.09E-06	6.82E-05
Fpr1	5.20	24.34	4.68	1.47E-06	8.81E-05
Olr1	2.66	12.10	4.55	1.36E-05	6.12E-04
Prdm1	9.79	42.97	4.39	1.76E-08	1.68E-06
Clec4e	351.58	1537.18	4.37	3.72E-21	3.13E-18
Saa3	16.10	70.19	4.36	2.48E-14	7.17E-12
I830012O16Rik	1.91	8.24	4.31	4.91E-05	1.78E-03
Sod2	156.84	674.23	4.30	1.07E-52	3.61E-49
Gbp2	8.94	37.53	4.20	4.64E-10	6.42E-08
Oasl1	4.67	19.26	4.12	8.74E-05	2.88E-03
Ifit3	12.02	49.16	4.09	1.52E-05	6.74E-04
Hp	3.93	16.04	4.09	4.02E-05	1.54E-03
C3	32.00	129.40	4.04	1.18E-04	3.73E-03
Cd38	3.27	13.18	4.03	4.89E-04	1.16E-02
Lox	6.64	25.80	3.89	7.69E-06	3.79E-04
Gbp7	8.23	31.55	3.83	1.36E-12	3.12E-10
Slc7a2	106.61	389.00	3.65	1.71E-19	1.08E-16
Pde4b	25.20	91.75	3.64	2.62E-20	1.89E-17
Ifit2	43.50	155.84	3.58	1.45E-16	6.67E-14
Flrt3	8.50	29.77	3.50	1.39E-06	8.40E-05
Cdc42ep2	11.37	37.77	3.32	2.49E-09	3.08E-07
Abtb2	2.73	8.98	3.29	1.57E-04	4.68E-03
Mx1	13.35	43.89	3.29	7.26E-05	2.49E-03
Cmklr1	44.41	143.86	3.24	1.25E-21	1.15E-18

Cmpk2	41.82	134.30	3.21	3.06E-07	2.23E-05
Slc7a11	31.46	98.59	3.13	4.91E-11	8.15E-09
Marcksl1	205.19	614.57	3.00	1.31E-19	8.81E-17
Gbgt1	4.96	14.79	2.98	2.22E-05	9.30E-04
Zc3h12c	71.21	209.55	2.94	5.60E-23	6.29E-20
Usp18	17.83	51.26	2.88	3.64E-09	4.19E-07
Ppap2b	13.99	39.66	2.84	2.39E-06	1.34E-04
Jag1	63.79	180.85	2.84	9.03E-12	1.66E-09
Cd274	49.25	138.16	2.81	2.56E-17	1.29E-14
Mx2	6.47	17.87	2.76	2.13E-06	1.22E-04
Pyhin1	6.70	18.50	2.76	6.76E-07	4.47E-05
Aoah	43.79	120.45	2.75	1.55E-08	1.49E-06
Vcan	14.15	38.58	2.73	2.35E-08	2.15E-06
Ralgds	75.27	204.17	2.71	8.88E-05	2.89E-03
Gm12250	9.12	24.70	2.71	1.94E-12	4.28E-10
Src	16.28	44.05	2.71	1.96E-05	8.36E-04
Pilra	35.07	93.61	2.67	7.48E-10	1.01E-07
Klf7	20.11	53.49	2.66	1.24E-08	1.20E-06
Slfn5	273.75	721.80	2.64	2.38E-14	7.10E-12
Icam1	147.60	387.99	2.63	2.95E-06	1.60E-04
Acsl1	144.71	380.05	2.63	3.99E-28	5.76E-25
Cxcl2	38.26	99.81	2.61	6.72E-09	7.24E-07
Traf1	19.59	50.92	2.60	4.49E-07	3.07E-05
Birc3	76.13	197.26	2.59	2.06E-12	4.39E-10
Trim30d	4.17	10.78	2.59	2.01E-04	5.73E-03
Nfkbia	224.06	574.41	2.56	1.21E-20	9.38E-18
Tlr2	181.72	463.31	2.55	8.67E-22	8.77E-19
Zmynd15	7.73	19.68	2.54	8.84E-04	1.92E-02
Fas	12.43	31.40	2.53	2.09E-06	1.20E-04
Parp14	248.42	622.34	2.51	8.19E-18	4.36E-15
Ehd1	225.55	561.97	2.49	2.37E-14	7.10E-12
Tgm2	94.71	235.90	2.49	4.35E-09	4.79E-07
Arl5c	36.76	90.40	2.46	1.05E-09	1.37E-07
Tnfaip3	75.21	184.11	2.45	2.11E-09	2.67E-07
Fgr	112.52	270.66	2.41	5.12E-17	2.47E-14
Ccrl2	23.05	55.30	2.40	4.06E-05	1.54E-03
Nod2	13.10	30.77	2.35	1.47E-06	8.81E-05
Irak3	61.55	144.54	2.35	4.11E-11	6.92E-09
Slc31a2	77.47	181.02	2.34	1.19E-14	3.90E-12
Spata13	58.60	134.56	2.30	9.39E-09	9.59E-07
Isg15	21.90	49.03	2.24	3.96E-10	5.64E-08
Ddx58	110.50	245.47	2.22	3.18E-09	3.78E-07
Slc15a3	185.03	410.49	2.22	2.66E-10	3.90E-08

Dst	405.12	891.42	2.20	2.99E-13	7.57E-11
Slfn8	94.25	207.19	2.20	5.78E-11	9.43E-09
Amotl1	17.07	37.38	2.19	1.75E-04	5.11E-03
Zbp1	17.92	39.21	2.19	3.04E-09	3.70E-07
N4bp1	77.45	168.30	2.17	1.18E-13	3.06E-11
Slc11a2	103.79	225.49	2.17	8.12E-13	1.96E-10
Cflar	271.05	588.44	2.17	1.08E-18	6.41E-16
Tnfrsf1b	388.65	842.53	2.17	9.61E-10	1.26E-07
Ets2	207.98	450.36	2.17	7.99E-09	8.42E-07
Pion	154.16	332.95	2.16	1.16E-13	3.06E-11
Malt1	193.93	417.67	2.15	3.32E-10	4.80E-08
Itgal	85.40	183.67	2.15	7.39E-08	6.12E-06
Slc7a8	255.30	545.22	2.14	3.87E-09	4.35E-07
Irf7	31.14	66.01	2.12	7.92E-05	2.69E-03
Nfkbie	81.14	171.60	2.11	8.82E-06	4.32E-04
Acp2	249.25	526.91	2.11	3.46E-16	1.52E-13
Pstpip2	84.17	177.89	2.11	2.08E-12	4.39E-10
Rtp4	54.69	115.56	2.11	1.23E-11	2.23E-09
Igsf6	201.03	421.47	2.10	4.59E-09	4.99E-07
Tnip1	164.89	344.84	2.09	3.46E-09	4.02E-07
Nr1h3	18.63	38.50	2.07	3.34E-04	8.67E-03
Ptgir	28.79	59.25	2.06	4.81E-05	1.77E-03
Helz2	152.60	312.68	2.05	1.13E-04	3.61E-03
Dcbld2	98.82	201.18	2.04	1.01E-07	8.09E-06
Cers6	152.52	309.93	2.03	2.86E-15	1.03E-12
Rassf4	991.55	2011.78	2.03	3.40E-11	5.83E-09
Ifi47	22.17	44.94	2.03	7.07E-08	5.96E-06
Slc4a7	264.79	535.93	2.02	1.14E-08	1.12E-06
Znfx1	387.94	784.08	2.02	2.67E-11	4.66E-09
Slc16a10	253.35	509.32	2.01	5.48E-14	1.50E-11
Agtrap	85.54	171.32	2.00	7.01E-08	5.96E-06
**Down-regulated**					
Msh5	43.56	14.75	0.34	1.12E-06	6.97E-05
Eps8	42.41	16.85	0.40	2.64E-06	1.45E-04
Rgs1	192.15	76.55	0.40	1.56E-07	1.21E-05
2700094K13Rik	33.46	13.66	0.41	1.78E-04	5.16E-03
Cd28	61.41	25.16	0.41	8.84E-06	4.32E-04
Mrps6	27.97	11.78	0.42	6.25E-04	1.43E-02
Rpp21	22.05	9.39	0.43	2.27E-04	6.38E-03
Rnf150	179.35	78.31	0.44	2.28E-06	1.29E-04
Tmem256	45.93	20.69	0.45	7.05E-05	2.43E-03
Ypel3	40.83	18.47	0.45	9.72E-05	3.15E-03

Snhg5	33.11	15.18	0.46	7.97E-04	1.77E-02
D4Wsu53e	213.77	99.88	0.47	2.35E-10	3.49E-08
Klf2	68.78	32.14	0.47	2.51E-06	1.39E-04
Fabp5	460.38	215.87	0.47	1.85E-06	1.09E-04
Hmga2-ps1	60.55	29.13	0.48	3.02E-05	1.21E-03
Rgs2	352.10	172.85	0.49	1.78E-12	4.00E-10

Supplemental Table 2 – Genes regulated in IFN vs Control

Gene	Control	IFN	FC	P-Value	FDR
**Up-regulated**					
Nos2	0.09	467.53	5086.61	1.36E-42	1.72E-40
Iigp1	0.84	2513.68	3002.20	1.54E-200	3.12E-197
Gbp5	1.57	1856.06	1181.67	0.00E+00	0.00E+00
Gbp2	8.94	5971.11	667.68	6.06E-121	4.08E-118
Cxcl10	6.59	2919.06	442.95	2.12E-83	7.96E-81
Gbp6	1.40	591.92	421.55	1.01E-150	8.53E-148
Gbp3	3.66	774.23	211.27	2.74E-110	1.38E-107
Gbp7	8.23	1351.47	164.21	5.70E-162	5.77E-159
Batf2	2.35	370.89	157.89	2.00E-110	1.06E-107
Cd40	2.10	266.54	127.06	1.34E-63	3.40E-61
Fgl2	9.08	929.54	102.39	1.95E-73	6.17E-71
F830016B08Rik	3.62	278.42	76.81	1.64E-27	1.07E-25
Gm12250	9.12	664.02	72.81	1.48E-219	7.47E-216
Ptgs2	2.56	149.03	58.21	8.97E-45	1.30E-42
Cd69	2.42	140.54	58.12	1.21E-47	1.86E-45
Irg1	72.85	4124.57	56.61	2.43E-162	2.74E-159
Rsad2	31.16	1607.60	51.60	1.81E-92	8.30E-90
Socs3	4.10	205.13	50.07	3.79E-14	9.71E-13
Arid5a	4.21	178.84	42.52	4.46E-20	1.84E-18
Ifi47	22.17	935.11	42.17	1.17E-164	1.48E-161
Cd274	49.25	1964.52	39.89	5.20E-174	8.76E-171
Zbp1	17.92	714.16	39.85	3.51E-148	2.73E-145
Oasl1	4.67	182.72	39.09	9.68E-18	3.36E-16
Mx1	13.35	516.85	38.72	3.08E-24	1.66E-22
Fcgr4	6.49	240.30	37.05	2.28E-14	5.95E-13
Ly6a	3.43	126.69	36.89	6.31E-11	1.14E-09
C3	32.00	1154.78	36.09	9.70E-21	4.25E-19
Irf1	56.05	1847.79	32.97	9.25E-216	3.12E-212
Ifit2	43.50	1385.05	31.84	1.31E-80	4.72E-78
Thbs1	2.22	66.05	29.71	3.26E-06	3.03E-05
Gbp9	27.40	790.84	28.86	1.14E-161	1.05E-158
Cfb	3.45	98.20	28.42	2.63E-16	8.22E-15
Igtp	25.81	718.49	27.83	9.36E-88	3.95E-85
Tnfsf10	8.29	214.95	25.94	1.23E-24	6.78E-23
Irgm1	86.39	1916.14	22.18	4.05E-204	1.02E-200
Trim30d	4.17	89.03	21.36	4.39E-24	2.33E-22
Zyx	131.95	2567.72	19.46	1.61E-31	1.30E-29
Pydc3	1.76	33.14	18.86	4.97E-18	1.75E-16
Irgm2	32.66	615.56	18.85	3.59E-142	2.59E-139
Cd38	3.27	60.35	18.45	1.16E-12	2.51E-11

Ifi44	5.83	106.70	18.29	8.49E-25	4.82E-23
Ifit1	20.42	350.14	17.15	1.61E-78	5.26E-76
Abtb2	2.73	45.37	16.62	9.08E-28	6.13E-26
Vcan	14.15	235.09	16.62	1.10E-70	3.27E-68
Il18rap	2.99	49.39	16.54	2.78E-22	1.31E-20
Cmpk2	41.82	676.87	16.18	2.26E-23	1.15E-21
Gdap10	16.92	270.11	15.96	1.61E-11	3.06E-10
Nod1	13.60	215.06	15.82	2.27E-20	9.63E-19
Dusp10	4.44	65.95	14.87	3.22E-13	7.55E-12
Cd74	12.75	189.44	14.86	7.59E-13	1.70E-11
Ccnd2	18.35	272.60	14.85	6.18E-06	5.46E-05
Stat1	192.40	2745.57	14.27	7.35E-169	1.06E-165
Tap1	75.89	1075.84	14.18	4.81E-112	2.70E-109
Pyhin1	6.70	94.75	14.15	1.82E-42	2.28E-40
Gm12185	7.95	112.44	14.14	3.17E-22	1.48E-20
Mnda	10.47	147.86	14.13	1.82E-57	3.41E-55
Ccl7	6.04	84.86	14.04	1.81E-04	1.14E-03
Lrrc4	2.91	39.64	13.65	6.44E-05	4.54E-04
Cd86	11.63	148.22	12.74	4.36E-35	4.60E-33
Isg15	21.90	275.36	12.57	1.14E-78	3.99E-76
Gm4759	7.48	93.06	12.44	2.97E-26	1.79E-24
Ifit3	12.02	146.93	12.22	4.41E-12	8.90E-11
Samhd1	409.13	4981.36	12.18	5.48E-60	1.18E-57
Ifi204	41.50	504.15	12.15	3.07E-34	3.04E-32
Ppargc1b	8.26	100.21	12.14	1.40E-19	5.53E-18
Tgm2	94.71	1139.81	12.03	2.14E-54	3.74E-52
Icam1	147.60	1742.47	11.81	6.35E-30	4.98E-28
Mx2	6.47	75.64	11.70	1.13E-32	9.64E-31
Il15ra	5.37	62.50	11.63	5.40E-24	2.84E-22
Tnfsf15	1.56	17.47	11.18	7.49E-10	1.20E-08
Rab43	92.12	1026.25	11.14	9.58E-45	1.36E-42
Slc7a2	106.61	1170.98	10.98	2.64E-52	4.38E-50
Irf7	31.14	339.69	10.91	2.39E-26	1.45E-24
Tapbpl	21.98	238.98	10.87	3.71E-64	9.63E-62
Ralgds	75.27	807.38	10.73	1.08E-20	4.69E-19
Nlrc5	79.58	850.38	10.69	3.08E-99	1.48E-96
Prr5l	12.63	131.37	10.40	4.68E-10	7.68E-09
Themis2	131.92	1343.48	10.18	8.00E-44	1.11E-41
Usp18	17.83	179.40	10.06	3.62E-29	2.71E-27
AW112010	2.54	25.53	10.04	1.11E-03	5.61E-03
Tnfrsf14	22.75	225.67	9.92	1.33E-24	7.32E-23
Parp14	248.42	2342.10	9.43	4.86E-69	1.41E-66
Mxd1	19.82	180.15	9.09	5.79E-28	4.01E-26

Il18bp	5.74	51.61	8.99	1.39E-07	1.62E-06
Oas3	144.91	1290.05	8.90	2.51E-33	2.29E-31
Arid5b	42.02	368.43	8.77	2.72E-59	5.74E-57
Ms4a4c	3.21	28.09	8.76	6.24E-13	1.41E-11
Acsl1	144.71	1262.62	8.73	1.54E-112	9.19E-110
I830012O16Rik	1.91	16.56	8.65	2.38E-10	4.02E-09
Fam46c	9.07	77.70	8.57	1.23E-27	8.15E-26
Ifi203	30.75	260.83	8.48	5.09E-18	1.79E-16
Trex1	45.42	384.83	8.47	3.92E-28	2.73E-26
Fcgr1	121.62	1020.97	8.39	4.75E-36	5.11E-34
Irf8	228.84	1919.56	8.39	9.05E-28	6.13E-26
Nampt	293.65	2446.94	8.33	3.53E-114	2.23E-111
Cdc42ep2	11.37	90.85	7.99	1.21E-19	4.81E-18
Tap2	83.72	668.70	7.99	2.98E-17	1.01E-15
Stat2	132.46	1047.77	7.91	2.05E-62	4.94E-60
Mndal	5.10	39.93	7.83	4.50E-13	1.04E-11
Ch25h	70.70	552.83	7.82	1.00E-22	4.95E-21
Il4ra	42.70	327.13	7.66	9.57E-23	4.77E-21
Slfn8	94.25	715.94	7.60	3.55E-38	4.09E-36
Igf2bp2	28.28	212.91	7.53	1.81E-46	2.65E-44
Tagap	16.03	119.19	7.44	1.68E-17	5.75E-16
Gpr132	10.82	79.61	7.36	6.06E-12	1.21E-10
Tmem173	70.42	517.86	7.35	1.41E-71	4.31E-69
Wars	107.95	788.82	7.31	3.83E-28	2.69E-26
Dtx3l	136.02	977.28	7.18	5.96E-89	2.62E-86
Slfn5	273.75	1959.72	7.16	1.32E-37	1.50E-35
Ccl2	51.36	361.54	7.04	1.08E-09	1.67E-08
Herc6	46.39	324.17	6.99	1.65E-57	3.15E-55
Phf11a	5.04	35.13	6.96	2.35E-12	4.96E-11
Phf11d	15.48	107.74	6.96	9.57E-29	7.06E-27
Phf11b	10.52	72.66	6.91	1.48E-08	2.01E-07
Enpp4	6.07	41.83	6.90	2.75E-16	8.57E-15
Serpinb9	32.15	221.27	6.88	7.10E-50	1.14E-47
Ggct	3.37	23.13	6.87	3.75E-04	2.16E-03
Rnf19b	152.85	1048.75	6.86	1.22E-84	4.76E-82
Rmdn3	35.19	240.68	6.84	3.58E-16	1.09E-14
Oasl2	125.62	850.07	6.77	1.33E-78	4.48E-76
Csrnp1	11.89	79.33	6.67	2.56E-15	7.33E-14
Prkce	3.24	21.55	6.66	1.13E-06	1.14E-05
Daxx	62.21	408.85	6.57	1.12E-16	3.64E-15
Kdm6b	16.66	108.55	6.52	2.05E-04	1.28E-03
C1rl	5.81	37.30	6.42	5.10E-12	1.02E-10
Sp110	89.90	562.28	6.25	1.36E-60	3.06E-58

Gm5431	21.29	132.78	6.24	6.58E-33	5.84E-31
Sh3bp4	11.32	70.40	6.22	1.96E-18	7.02E-17
Itpr1	68.92	428.39	6.22	4.06E-48	6.31E-46
Nod2	13.10	81.33	6.21	3.97E-20	1.65E-18
Otud1	21.43	132.92	6.20	5.42E-12	1.08E-10
Snord55	2.88	17.79	6.18	7.37E-04	3.93E-03
Ppp1r26	4.67	28.74	6.15	2.33E-10	3.95E-09
Notch1	103.46	635.26	6.14	2.27E-19	8.80E-18
Ifih1	89.47	548.93	6.14	2.52E-66	6.89E-64
Helz2	152.60	934.65	6.12	3.04E-20	1.28E-18
St6galnac4	44.07	269.76	6.12	2.53E-13	5.96E-12
Ifi202b	210.32	1271.04	6.04	1.47E-43	2.01E-41
Treml2	22.73	137.25	6.04	1.53E-06	1.51E-05
Casp4	34.12	205.51	6.02	2.28E-28	1.61E-26
Snord49a	32.71	195.39	5.97	2.27E-03	1.03E-02
Tmem67	13.83	82.20	5.94	2.11E-15	6.06E-14
Ccrl2	23.05	136.21	5.91	4.02E-16	1.22E-14
Il12rb2	11.30	65.11	5.76	4.77E-15	1.32E-13
Arhgef3	34.77	199.60	5.74	3.16E-36	3.44E-34
Rhoh	11.22	64.22	5.73	1.02E-04	6.79E-04
Tlr9	45.73	261.75	5.72	1.03E-09	1.60E-08
Bcl3	21.64	123.68	5.71	1.66E-08	2.23E-07
Gm8979	10.26	58.55	5.71	5.70E-07	6.02E-06
Pstpip2	84.17	480.16	5.70	1.11E-57	2.16E-55
Parp12	112.43	638.92	5.68	2.82E-60	6.19E-58
Inpp5b	91.17	516.47	5.66	2.56E-58	5.09E-56
Mov10	56.46	315.17	5.58	2.79E-41	3.40E-39
H2-DMa	14.27	79.54	5.57	3.19E-11	5.96E-10
Dhx58	50.15	279.14	5.57	1.24E-33	1.16E-31
Gbgt1	4.96	27.16	5.48	6.59E-10	1.06E-08
H2-T24	108.44	592.62	5.47	2.02E-58	4.08E-56
Pml	100.39	544.97	5.43	1.41E-54	2.50E-52
Znfx1	387.94	2104.25	5.42	4.97E-44	6.98E-42
Mlkl	32.54	176.03	5.41	2.88E-15	8.21E-14
Parp11	28.68	154.74	5.40	2.94E-11	5.52E-10
Pde7b	10.84	58.27	5.37	6.71E-12	1.33E-10
Trafd1	125.90	672.86	5.34	2.71E-40	3.23E-38
Spata13	58.60	312.04	5.32	1.79E-28	1.28E-26
Myo1d	18.19	96.79	5.32	3.04E-21	1.36E-19
Xaf1	24.60	130.48	5.30	2.57E-16	8.09E-15
Flt1	50.25	263.16	5.24	1.51E-07	1.75E-06
Cers6	152.52	787.56	5.16	5.39E-65	1.43E-62
9430020K01Rik	5.15	26.42	5.13	3.11E-07	3.42E-06

Il21r	14.52	74.49	5.13	7.54E-16	2.26E-14
Psmb10	59.53	305.23	5.13	3.79E-11	7.00E-10
Nlrc4	50.94	260.50	5.11	1.12E-38	1.30E-36
Stat3	197.99	1009.77	5.10	1.33E-62	3.29E-60
Il1rn	122.29	621.81	5.08	1.07E-52	1.80E-50
Rgl1	28.81	146.45	5.08	4.65E-11	8.53E-10
Cd300lf	22.91	115.48	5.04	8.08E-27	5.05E-25
Ank2	15.11	76.01	5.03	3.03E-09	4.48E-08
Stx11	22.15	111.39	5.03	3.94E-24	2.11E-22
Dnahc17	7.59	38.04	5.01	5.93E-11	1.08E-09
Phlpp1	51.71	259.02	5.01	7.30E-37	8.11E-35
Parp10	43.17	215.10	4.98	1.08E-32	9.24E-31
1600014C10Rik	29.79	148.13	4.97	9.08E-26	5.37E-24
Adamtsl4	7.68	37.28	4.85	3.61E-12	7.41E-11
Fas	12.43	60.08	4.83	9.32E-15	2.53E-13
Trim30a	157.05	753.41	4.80	5.06E-61	1.16E-58
Maff	6.49	31.08	4.79	1.13E-09	1.74E-08
Itgal	85.40	405.82	4.75	1.34E-26	8.33E-25
Lpar1	13.48	63.97	4.75	3.80E-06	3.48E-05
Tmem229b	102.91	483.56	4.70	4.60E-43	6.13E-41
Aoah	43.79	205.71	4.70	2.29E-14	5.95E-13
Parp9	90.29	421.17	4.66	3.39E-43	4.57E-41
Csf2rb	321.50	1495.32	4.65	7.52E-59	1.55E-56
Phf11c	2.63	12.15	4.61	4.13E-05	3.01E-04
Taf4b	6.39	29.44	4.61	8.90E-05	6.04E-04
Prkx	127.64	587.13	4.60	1.18E-34	1.21E-32
Jdp2	15.78	72.52	4.60	1.00E-15	2.96E-14
Psmb9	31.86	146.40	4.60	4.05E-13	9.43E-12
Parp3	69.33	318.34	4.59	1.87E-23	9.60E-22
Zbtb5	17.87	81.05	4.54	5.54E-06	4.93E-05
Abhd16a	57.57	260.86	4.53	1.03E-32	8.90E-31
Creb5	50.92	230.26	4.52	2.48E-24	1.36E-22
4933412E12Rik	4.35	19.63	4.51	3.53E-04	2.05E-03
Csf2rb2	210.03	945.56	4.50	4.32E-51	7.04E-49
Niacr1	26.64	119.59	4.49	1.04E-15	3.06E-14
Prdm1	9.79	43.83	4.47	1.19E-08	1.63E-07
Uba7	100.48	447.24	4.45	1.90E-14	4.99E-13
Mmp14	39.41	174.59	4.43	1.02E-31	8.36E-30
Ctsc	538.74	2381.21	4.42	3.67E-15	1.03E-13
Slc15a3	185.03	816.02	4.41	6.37E-35	6.64E-33
Trim21	36.23	159.69	4.41	3.54E-27	2.27E-25
Trib3	5.71	25.16	4.41	6.16E-04	3.36E-03
Pnp	129.78	568.90	4.38	8.84E-47	1.32E-44

Slc28a2	9.46	41.31	4.37	8.78E-11	1.56E-09
Il10ra	136.59	593.89	4.35	1.28E-42	1.64E-40
Sp140	44.63	191.74	4.30	8.64E-28	5.91E-26
Crim1	21.01	89.65	4.27	2.62E-16	8.21E-15
1700025N23Rik	7.25	30.93	4.26	2.31E-04	1.41E-03
Esr1	7.06	30.08	4.26	2.99E-07	3.30E-06
Tmco4	22.37	94.79	4.24	1.25E-12	2.71E-11
Mast4	35.96	151.95	4.22	4.95E-07	5.26E-06
Atp10a	12.93	54.50	4.22	6.78E-11	1.21E-09
Dram1	164.41	690.43	4.20	1.23E-41	1.52E-39
Tapbp	442.00	1851.13	4.19	2.09E-09	3.13E-08
Unc5a	4.22	17.61	4.17	6.80E-06	5.94E-05
Tnfaip2	321.47	1339.83	4.17	2.56E-13	6.00E-12
Fam102b	152.67	635.18	4.16	2.81E-27	1.82E-25
Lrrc16a	27.06	112.44	4.16	2.78E-21	1.25E-19
Mrgpre	2.99	12.40	4.15	1.31E-03	6.49E-03
Xkr8	15.60	64.67	4.15	4.13E-12	8.37E-11
Tnfaip3	75.21	311.28	4.14	1.31E-16	4.20E-15
Hivep2	55.94	231.48	4.14	1.03E-27	6.85E-26
6330407A03Rik	5.08	20.97	4.13	2.41E-05	1.87E-04
8430408G22Rik	3.48	14.34	4.13	7.01E-04	3.75E-03
Golga3	126.42	520.74	4.12	5.56E-41	6.70E-39
P2ry14	7.39	30.34	4.11	2.05E-07	2.32E-06
Peli1	58.90	241.35	4.10	1.28E-12	2.76E-11
Rapgef2	86.31	353.60	4.10	5.78E-09	8.23E-08
Itga4	546.95	2236.45	4.09	3.23E-55	5.84E-53
Lacc1	23.17	94.35	4.07	4.35E-16	1.31E-14
Tanc2	62.48	254.40	4.07	1.18E-28	8.66E-27
Zfp281	71.31	290.30	4.07	4.20E-11	7.73E-10
Vmn1r65	7.68	31.24	4.07	1.23E-05	1.00E-04
Katna1	40.32	163.75	4.06	1.32E-21	6.01E-20
Synpo	9.26	37.59	4.06	8.09E-04	4.26E-03
Slc2a6	138.21	560.37	4.05	1.05E-08	1.44E-07
Ccdc88b	133.58	541.12	4.05	5.69E-36	6.06E-34
Slc23a2	443.88	1790.89	4.03	4.34E-57	7.98E-55
Agpat9	5.11	20.59	4.03	2.63E-06	2.48E-05
Gpd2	65.47	261.94	4.00	5.37E-27	3.39E-25
Crem	10.57	42.19	3.99	1.84E-06	1.79E-05
Adap2	79.46	316.88	3.99	8.39E-33	7.32E-31
Sod2	156.84	620.44	3.96	4.89E-47	7.38E-45
Pde4b	25.20	99.52	3.95	1.68E-17	5.75E-16
Atp8a1	131.96	518.00	3.93	1.30E-22	6.32E-21
1-Mar	84.15	330.30	3.93	7.76E-33	6.82E-31

Etv6	93.02	363.32	3.91	1.41E-32	1.17E-30
Pgs1	81.24	317.10	3.90	4.24E-20	1.76E-18
Cp	3.97	15.44	3.89	4.93E-05	3.53E-04
5031414D18Rik	16.23	62.83	3.87	9.16E-14	2.28E-12
Kdr	34.66	133.68	3.86	1.88E-13	4.49E-12
Hivep1	127.32	490.65	3.85	3.78E-34	3.64E-32
St14	15.88	61.19	3.85	1.86E-10	3.18E-09
Degs2	9.59	36.93	3.85	1.76E-09	2.67E-08
Chac1	3.54	13.62	3.84	8.06E-05	5.53E-04
Ogfr	83.91	321.77	3.83	2.19E-29	1.68E-27
Cdh23	2.93	11.22	3.83	2.07E-03	9.56E-03
Pfkp	278.68	1068.17	3.83	1.48E-37	1.66E-35
Naaa	38.10	145.33	3.81	3.31E-07	3.62E-06
Bambi-ps1	7.60	28.91	3.80	1.80E-03	8.49E-03
9930111J21Rik1	90.38	342.37	3.79	8.64E-28	5.91E-26
AI607873	335.64	1271.35	3.79	2.07E-33	1.92E-31
Tcf7l2	41.38	155.69	3.76	8.41E-20	3.39E-18
Il13ra1	22.98	86.43	3.76	2.01E-15	5.79E-14
P2ry13	7.08	26.60	3.76	2.43E-06	2.31E-05
St3gal5	57.19	214.77	3.76	3.87E-18	1.37E-16
Nckap5l	13.14	49.18	3.74	3.04E-03	1.31E-02
Rtp4	54.69	204.60	3.74	7.26E-29	5.40E-27
Vasn	36.81	137.65	3.74	3.94E-12	8.03E-11
Ppa1	26.77	99.83	3.73	7.80E-17	2.58E-15
Mt2	52.33	194.30	3.71	2.73E-15	7.81E-14
Adam19	6.30	23.40	3.71	6.78E-07	7.05E-06
Zufsp	32.17	118.92	3.70	1.14E-17	3.94E-16
Gca	12.29	45.41	3.70	2.83E-12	5.90E-11
Cmklr1	44.41	163.24	3.68	7.05E-27	4.43E-25
Hk3	476.62	1749.36	3.67	2.19E-08	2.88E-07
Birc3	76.13	277.41	3.64	6.35E-16	1.91E-14
C1ra	11.29	41.02	3.63	3.99E-04	2.28E-03
Parp8	45.22	163.75	3.62	1.57E-22	7.54E-21
Lrrc8c	63.54	229.86	3.62	3.01E-24	1.63E-22
Naa25	62.07	223.27	3.60	2.63E-24	1.43E-22
Rab20	8.70	31.08	3.57	2.16E-03	9.92E-03
Cacna1b	8.23	29.25	3.56	2.80E-04	1.67E-03
Rrp12	46.08	163.40	3.55	3.30E-19	1.26E-17
1110038F14Rik	20.59	72.77	3.53	9.55E-11	1.68E-09
Ifi35	34.73	122.50	3.53	8.00E-19	2.97E-17
Tmem2	58.19	204.73	3.52	1.28E-21	5.85E-20
Htt	305.40	1074.20	3.52	5.75E-43	7.55E-41
St7	9.98	35.05	3.51	1.07E-05	8.83E-05

Lpp	133.35	468.08	3.51	2.99E-18	1.06E-16
Furin	131.41	460.83	3.51	1.50E-29	1.16E-27
Eng	47.33	165.66	3.50	1.22E-12	2.65E-11
Bcl9	62.96	219.74	3.49	2.61E-14	6.75E-13
Fcgr3	277.25	957.72	3.45	6.41E-34	6.06E-32
Rhbdf2	113.23	390.79	3.45	2.01E-14	5.24E-13
Nfkbie	81.14	279.85	3.45	2.13E-10	3.63E-09
Klf7	20.11	69.12	3.44	5.01E-09	7.21E-08
Tagln	2.41	8.28	3.43	2.73E-03	1.20E-02
Sbno2	185.18	634.39	3.43	2.88E-29	2.19E-27
Klhl6	53.07	181.38	3.42	7.37E-18	2.57E-16
Apobec3	70.99	241.64	3.40	4.01E-04	2.29E-03
Lrch1	59.70	202.86	3.40	7.77E-22	3.59E-20
H2-T23	99.43	337.43	3.39	4.66E-08	5.85E-07
Larp1	431.74	1463.75	3.39	3.49E-33	3.16E-31
Noc4l	37.65	127.62	3.39	9.66E-14	2.40E-12
Pim1	93.32	315.52	3.38	1.46E-28	1.05E-26
Tnfrsf1a	227.98	770.64	3.38	2.20E-34	2.22E-32
Sema4a	45.27	152.72	3.37	7.80E-09	1.10E-07
Src	16.28	54.84	3.37	5.26E-07	5.58E-06
Pmepa1	32.29	108.41	3.36	3.06E-09	4.50E-08
Psd	4.40	14.76	3.35	1.04E-03	5.30E-03
Ccdc25	40.69	135.52	3.33	3.26E-10	5.44E-09
Lap3	56.29	187.48	3.33	4.53E-19	1.72E-17
Oas2	81.49	270.92	3.32	7.61E-09	1.07E-07
Tbc1d9	108.68	361.22	3.32	1.19E-12	2.59E-11
Gpr141	58.24	192.79	3.31	3.41E-05	2.54E-04
Chd7	66.22	219.02	3.31	3.78E-23	1.90E-21
Ripk2	25.97	85.58	3.30	4.18E-12	8.47E-11
4930431F12Rik	6.77	22.27	3.29	1.05E-05	8.67E-05
Pla2g16	33.00	108.38	3.28	1.09E-14	2.93E-13
Eif2ak2	174.58	572.25	3.28	1.69E-31	1.35E-29
Socs7	55.93	183.28	3.28	7.15E-20	2.90E-18
Adar	162.98	533.03	3.27	3.41E-34	3.32E-32
Nfix	14.71	48.12	3.27	9.27E-04	4.78E-03
Ikzf1	186.65	610.12	3.27	1.90E-31	1.52E-29
Slc31a1	228.23	745.76	3.27	2.43E-33	2.24E-31
Stxbp1	110.83	362.02	3.27	1.17E-24	6.56E-23
Dexi	12.77	41.63	3.26	2.24E-06	2.14E-05
Tifab	112.59	366.75	3.26	4.02E-19	1.53E-17
Nmi	25.44	82.86	3.26	1.10E-12	2.42E-11
Wdtc1	82.02	265.23	3.23	4.42E-13	1.02E-11
Chic1	7.01	22.61	3.22	6.03E-04	3.30E-03

Fam102a	29.94	96.34	3.22	1.97E-05	1.55E-04
Hk2	143.92	462.93	3.22	4.07E-33	3.65E-31
Gpr84	40.92	131.38	3.21	5.69E-13	1.30E-11
2310042D19Rik	11.11	35.50	3.20	9.16E-04	4.73E-03
Coa5	190.84	609.88	3.20	1.26E-32	1.05E-30
Bst1	38.64	123.30	3.19	8.70E-08	1.05E-06
Disc1	4.18	13.34	3.19	1.27E-03	6.32E-03
Ccdc86	33.76	107.64	3.19	3.95E-15	1.11E-13
Dennd1a	152.31	484.17	3.18	2.34E-26	1.43E-24
Cflar	271.05	861.12	3.18	2.02E-34	2.06E-32
Sp100	131.90	418.33	3.17	1.43E-26	8.84E-25
Fgd2	28.15	88.95	3.16	8.70E-07	8.96E-06
Rnf217	18.14	57.30	3.16	5.85E-09	8.31E-08
Slamf7	134.53	424.85	3.16	1.22E-28	8.90E-27
Cdkn1a	298.28	939.84	3.15	7.23E-12	1.42E-10
Nuak2	34.43	108.20	3.14	9.90E-13	2.19E-11
Tmcc3	29.21	91.67	3.14	6.82E-13	1.54E-11
Tor3a	138.80	434.15	3.13	1.45E-28	1.05E-26
Hk1	324.92	1015.18	3.12	5.48E-21	2.43E-19
Ddx60	33.02	103.01	3.12	9.85E-15	2.66E-13
Rnf31	79.45	247.37	3.11	1.35E-18	4.86E-17
Arhgap23	30.09	93.54	3.11	2.10E-06	2.02E-05
Lcp2	167.15	519.50	3.11	3.93E-13	9.16E-12
Slc12a9	41.43	128.56	3.10	3.03E-06	2.83E-05
Vrk2	41.86	129.50	3.09	2.33E-11	4.39E-10
Acvrl1	70.77	217.39	3.07	4.19E-10	6.95E-09
Slfn2	435.65	1334.27	3.06	3.22E-34	3.16E-32
Zfp36	131.21	401.48	3.06	8.32E-24	4.34E-22
Cdyl2	38.57	117.82	3.05	7.46E-14	1.86E-12
Enc1	43.74	133.51	3.05	6.01E-14	1.51E-12
Psmb8	161.05	490.07	3.04	2.85E-20	1.20E-18
L3mbtl3	9.81	29.74	3.03	3.05E-04	1.81E-03
Zfp777	19.08	57.87	3.03	3.05E-07	3.36E-06
Gm7609	17.73	53.57	3.02	4.65E-08	5.84E-07
Clcn7	141.14	424.81	3.01	8.69E-25	4.91E-23
N4bp1	77.45	233.03	3.01	2.18E-19	8.50E-18
Axl	364.55	1089.99	2.99	3.91E-03	1.62E-02
Pnp2	4.32	12.90	2.99	1.48E-03	7.20E-03
Soat2	5.91	17.62	2.98	4.37E-03	1.78E-02
Gnb4	85.66	254.94	2.98	2.53E-22	1.20E-20
Fzd7	55.27	164.41	2.97	7.45E-12	1.46E-10
Kcna3	8.07	24.00	2.97	1.63E-05	1.30E-04
Oas1a	47.31	140.57	2.97	5.86E-14	1.48E-12

Rrbp1	762.62	2249.38	2.95	5.53E-34	5.28E-32
Mafk	60.02	176.67	2.94	1.02E-15	3.01E-14
Tmem132a	22.31	65.51	2.94	2.52E-04	1.53E-03
Slc31a2	77.47	226.15	2.92	1.90E-17	6.47E-16
5-Mar	111.03	323.84	2.92	1.58E-20	6.81E-19
Bend6	6.05	17.63	2.92	3.49E-04	2.03E-03
Hipk2	189.65	551.70	2.91	3.03E-22	1.42E-20
Capn1	130.92	380.32	2.91	4.06E-14	1.04E-12
Ubr4	994.40	2878.44	2.89	1.24E-32	1.05E-30
Gpc1	28.64	82.86	2.89	2.34E-12	4.96E-11
Oas1g	13.35	38.60	2.89	1.21E-04	7.95E-04
Rmi2	18.89	54.51	2.89	1.50E-05	1.20E-04
Ubash3b	145.70	420.08	2.88	3.82E-22	1.77E-20
Sdc3	1089.78	3127.15	2.87	1.48E-08	2.00E-07
Bak1	256.15	734.42	2.87	2.44E-25	1.42E-23
Tpst1	26.80	76.82	2.87	1.76E-10	3.03E-09
Myh10	7.70	22.02	2.86	4.11E-03	1.68E-02
Creb3	79.07	225.94	2.86	8.95E-16	2.65E-14
Extl3	199.40	568.61	2.85	7.59E-26	4.52E-24
Lifr	18.07	51.51	2.85	7.14E-04	3.82E-03
Sdad1	52.10	148.42	2.85	1.25E-16	4.05E-15
Tlr3	35.42	100.87	2.85	2.64E-12	5.54E-11
Jak2	86.96	247.60	2.85	2.30E-21	1.05E-19
Ddx58	110.50	313.42	2.84	8.14E-10	1.29E-08
Pfkfb3	79.64	225.84	2.84	1.75E-14	4.60E-13
Rbck1	92.73	262.10	2.83	1.19E-14	3.20E-13
Tnf	102.12	288.25	2.82	4.34E-19	1.65E-17
Tmed8	103.55	290.68	2.81	9.95E-20	4.00E-18
Asb13	15.89	44.57	2.80	9.44E-07	9.65E-06
D17Wsu92e	289.34	811.25	2.80	1.20E-24	6.68E-23
Arhgap27	53.94	151.19	2.80	6.33E-15	1.74E-13
Setdb2	33.29	93.06	2.80	2.96E-08	3.83E-07
Gpr65	53.40	149.24	2.79	2.37E-06	2.26E-05
Kif5c	9.12	25.47	2.79	1.15E-04	7.60E-04
Procr	8.69	24.23	2.79	8.51E-06	7.21E-05
Apba1	28.02	78.17	2.79	7.04E-10	1.13E-08
Piezo1	547.02	1524.50	2.79	2.08E-22	9.95E-21
1700071M16Rik	7.70	21.39	2.78	4.73E-04	2.65E-03
Itpkb	142.03	394.65	2.78	1.27E-20	5.50E-19
Max	120.06	333.21	2.78	2.11E-19	8.28E-18
Ehd1	225.55	625.95	2.78	1.07E-14	2.89E-13
Pcmtd1	100.94	279.93	2.77	3.73E-07	4.05E-06
Ppm1k	22.83	62.95	2.76	5.36E-08	6.68E-07

Triobp	60.92	167.84	2.76	4.52E-15	1.26E-13
Dbnl	137.75	379.14	2.75	6.02E-19	2.26E-17
Rap2c	100.70	277.02	2.75	5.97E-15	1.64E-13
Entpd1	104.16	285.64	2.74	3.30E-12	6.84E-11
Lmo4	80.33	219.60	2.73	9.47E-13	2.10E-11
Slc25a25	33.71	92.09	2.73	6.40E-12	1.27E-10
Ppp6r1	366.25	998.75	2.73	1.89E-20	8.12E-19
Slc12a7	124.09	337.86	2.72	8.30E-24	4.34E-22
Rtp3	6.75	18.35	2.72	6.72E-04	3.63E-03
Slc2a1	239.86	651.90	2.72	6.05E-25	3.48E-23
Ptges	8.58	23.25	2.71	5.30E-05	3.78E-04
Fbxw17	33.60	90.99	2.71	2.57E-12	5.42E-11
Eml4	102.71	278.03	2.71	7.78E-16	2.32E-14
Evl	120.25	325.20	2.70	4.83E-21	2.15E-19
Papd7	73.87	199.75	2.70	1.09E-16	3.57E-15
Nfkb2	137.73	371.86	2.70	7.32E-10	1.17E-08
Rnf135	18.44	49.73	2.70	8.28E-06	7.03E-05
Fgd6	78.07	210.55	2.70	1.48E-10	2.58E-09
Hck	211.23	569.56	2.70	1.02E-22	5.01E-21
Clec16a	105.81	284.67	2.69	3.13E-17	1.06E-15
Ttc39b	47.29	127.19	2.69	8.99E-15	2.45E-13
Cacna1d	53.30	142.80	2.68	4.23E-10	7.01E-09
Zcchc2	110.82	295.44	2.67	8.66E-19	3.18E-17
Mvp	253.34	673.50	2.66	1.49E-13	3.61E-12
Usp12	96.76	256.64	2.65	5.48E-19	2.07E-17
Rnf114	108.46	287.58	2.65	2.51E-09	3.73E-08
Traf1	19.59	51.87	2.65	1.79E-07	2.05E-06
Hspa2	9.57	25.34	2.65	8.77E-04	4.56E-03
Atp11b	135.54	358.42	2.64	2.36E-21	1.07E-19
Dst	405.12	1069.87	2.64	4.18E-17	1.41E-15
Ttyh3	251.62	662.72	2.63	1.13E-05	9.31E-05
Plau	853.75	2247.89	2.63	1.67E-08	2.23E-07
Bcl9l	25.42	66.66	2.62	8.55E-08	1.04E-06
Fbxl5	95.00	249.00	2.62	1.83E-19	7.19E-18
G530011O06Rik	60.75	159.20	2.62	1.81E-06	1.77E-05
Atp13a1	102.45	267.80	2.61	1.35E-07	1.58E-06
Tshz1	67.59	176.63	2.61	7.40E-12	1.45E-10
Slc25a22	33.99	88.61	2.61	9.91E-04	5.08E-03
Csf1	12.32	32.04	2.60	1.27E-03	6.33E-03
Aim1	202.44	526.20	2.60	2.92E-14	7.51E-13
Smpdl3b	59.59	154.86	2.60	4.46E-05	3.22E-04
Myd88	103.39	268.19	2.59	1.15E-07	1.36E-06
Rassf4	991.55	2562.31	2.58	1.91E-13	4.55E-12

Gna13	396.92	1024.80	2.58	1.51E-27	9.94E-26
Tma16	17.93	46.19	2.58	1.58E-08	2.13E-07
Cd83	25.98	66.92	2.58	6.78E-04	3.65E-03
Fam46a	74.60	192.12	2.58	7.91E-06	6.78E-05
Nrip1	45.84	118.03	2.57	5.04E-10	8.24E-09
Epsti1	66.57	171.16	2.57	1.70E-13	4.11E-12
1110018G07Rik	128.70	329.83	2.56	7.67E-20	3.10E-18
Cd180	620.17	1586.05	2.56	2.98E-25	1.73E-23
Trim56	68.48	174.92	2.55	6.68E-11	1.20E-09
C030046E11Rik	71.64	182.97	2.55	2.15E-08	2.83E-07
Lrp10	181.44	463.30	2.55	6.78E-16	2.03E-14
Ak4	39.80	101.59	2.55	3.43E-12	7.07E-11
Coro7	214.96	548.17	2.55	5.32E-20	2.18E-18
Slc8a1	152.01	387.63	2.55	5.97E-19	2.25E-17
Ppp1r15b	255.65	651.61	2.55	4.55E-23	2.28E-21
Slc30a1	71.56	182.19	2.55	4.80E-13	1.11E-11
Ninj1	164.31	417.88	2.54	3.51E-15	9.91E-14
B4galt3	21.30	54.09	2.54	1.40E-05	1.13E-04
Card6	17.09	43.35	2.54	3.60E-05	2.67E-04
Zfp800	45.06	114.03	2.53	6.80E-10	1.09E-08
Clic4	1000.35	2528.61	2.53	3.55E-27	2.27E-25
Dcp2	135.41	341.33	2.52	1.57E-09	2.39E-08
Mertk	107.88	271.77	2.52	1.39E-16	4.46E-15
Flnb	97.55	245.68	2.52	1.53E-16	4.88E-15
Mtmr14	96.85	243.23	2.51	5.98E-10	9.67E-09
Trim14	29.62	74.39	2.51	2.51E-08	3.28E-07
Slc16a3	398.71	1000.04	2.51	1.10E-08	1.51E-07
Mapkapk2	495.65	1243.00	2.51	2.31E-22	1.10E-20
Pphln1	107.86	270.09	2.50	1.05E-11	2.04E-10
Lipe	27.64	69.12	2.50	8.51E-04	4.45E-03
Nfkbia	224.06	559.94	2.50	9.73E-14	2.41E-12
Msantd3	7.69	19.22	2.50	1.38E-03	6.80E-03
Kars	129.08	322.15	2.50	1.25E-14	3.35E-13
Tlcd2	12.84	32.04	2.50	5.28E-04	2.93E-03
Ece1	89.05	222.05	2.49	4.61E-06	4.15E-05
Pi4ka	332.64	829.02	2.49	2.00E-20	8.55E-19
Basp1	402.32	1002.38	2.49	1.28E-08	1.74E-07
Phc2	122.62	304.50	2.48	7.55E-10	1.20E-08
Rffl	11.50	28.53	2.48	7.38E-04	3.93E-03
Arl4a	15.89	39.36	2.48	9.60E-06	8.01E-05
Samd4b	136.06	336.76	2.48	3.18E-16	9.75E-15
Klf8	16.46	40.68	2.47	4.27E-05	3.10E-04
Rnpepl1	108.25	267.29	2.47	3.77E-12	7.70E-11

Mid1	86.36	212.96	2.47	3.97E-06	3.62E-05
Mob3c	22.32	55.00	2.46	5.38E-05	3.82E-04
Smcr8	255.99	629.97	2.46	1.39E-22	6.75E-21
Serpinb6b	44.37	109.13	2.46	1.75E-03	8.26E-03
Tulp3	23.57	57.94	2.46	1.06E-07	1.26E-06
Rgmb	27.16	66.75	2.46	1.15E-07	1.36E-06
Tbkbp1	55.33	135.81	2.45	2.19E-05	1.71E-04
Esrra	42.99	105.41	2.45	1.40E-07	1.62E-06
Snap29	112.07	274.75	2.45	1.12E-16	3.64E-15
Lpin2	162.73	398.89	2.45	5.86E-20	2.39E-18
Kitl	40.05	98.08	2.45	4.75E-03	1.90E-02
Gna15	34.08	83.43	2.45	1.31E-08	1.78E-07
Fut4	8.73	21.36	2.45	2.59E-03	1.15E-02
Zmynd15	7.73	18.88	2.44	1.42E-04	9.18E-04
Homer1	19.30	47.08	2.44	1.35E-05	1.10E-04
Stx2	182.15	444.28	2.44	1.63E-17	5.61E-16
Ep400	331.81	807.42	2.43	3.67E-20	1.54E-18
Cmtm6	119.53	290.63	2.43	3.07E-16	9.48E-15
Rbbp8	66.94	162.58	2.43	3.81E-12	7.77E-11
Zbtb7b	152.00	368.41	2.42	1.23E-04	8.04E-04
Gnl1	75.12	181.84	2.42	8.90E-12	1.73E-10
Notch2	486.22	1175.56	2.42	1.92E-15	5.57E-14
Flot2	177.78	428.58	2.41	3.76E-16	1.14E-14
Pvrl4	17.56	42.29	2.41	1.54E-04	9.87E-04
Fmnl2	37.31	89.73	2.41	1.03E-08	1.42E-07
Med13	281.18	675.64	2.40	1.89E-09	2.85E-08
Cep85l	5.32	12.69	2.39	2.88E-03	1.25E-02
Irak2	126.57	301.96	2.39	2.01E-15	5.79E-14
Akap9	228.58	545.22	2.39	4.80E-17	1.60E-15
Samd9l	304.81	726.75	2.38	1.28E-14	3.41E-13
4933426M11Rik	302.90	721.63	2.38	7.33E-19	2.73E-17
Clasp1	156.68	372.91	2.38	1.92E-16	6.06E-15
Tgs1	97.82	232.52	2.38	2.27E-13	5.38E-12
Ptpn1	142.87	339.28	2.37	1.87E-16	5.93E-15
Nrp2	1268.97	3002.21	2.37	8.97E-24	4.65E-22
Tmem8	72.60	171.76	2.37	5.53E-10	9.00E-09
Pmvk	28.89	67.91	2.35	1.00E-05	8.34E-05
Snx10	148.60	348.47	2.35	4.57E-18	1.62E-16
Nfxl1	72.61	169.31	2.33	6.09E-13	1.38E-11
2610507B11Rik	1310.03	3044.00	2.32	1.99E-22	9.54E-21
Zmiz2	298.80	690.45	2.31	6.65E-07	6.93E-06
Aida	139.63	321.77	2.30	1.04E-13	2.57E-12
Ccr5	324.79	748.31	2.30	7.64E-16	2.28E-14

Abl2	190.02	437.77	2.30	7.89E-16	2.34E-14
Stoml1	15.94	36.59	2.30	9.92E-05	6.63E-04
Foxp4	24.56	56.37	2.29	8.45E-04	4.43E-03
Galk1	49.96	114.63	2.29	1.71E-08	2.29E-07
Rufy3	55.06	126.06	2.29	2.25E-07	2.53E-06
Lgals9	117.71	269.36	2.29	8.21E-05	5.62E-04
Dgkh	43.96	100.45	2.29	1.01E-05	8.40E-05
Sesn2	14.02	32.02	2.28	1.52E-04	9.72E-04
Frmd4a	128.61	293.68	2.28	1.05E-13	2.57E-12
Ahrr	19.00	43.38	2.28	4.36E-08	5.50E-07
Iqsec2	85.34	194.81	2.28	1.45E-03	7.09E-03
Aldh1b1	15.65	35.72	2.28	1.44E-05	1.16E-04
Vps54	67.54	153.85	2.28	3.64E-12	7.47E-11
Trps1	181.99	414.47	2.28	2.22E-07	2.51E-06
Agfg1	179.68	409.07	2.28	2.22E-16	7.01E-15
Trim12c	79.58	180.96	2.27	6.66E-13	1.50E-11
Pkib	40.21	91.14	2.27	2.61E-08	3.41E-07
Stat5b	102.62	232.38	2.26	3.59E-08	4.58E-07
Tor1aip2	318.11	719.46	2.26	4.80E-17	1.60E-15
Dck	201.29	454.66	2.26	8.79E-17	2.90E-15
Rest	86.16	194.48	2.26	9.37E-12	1.82E-10
Cndp2	500.20	1128.56	2.26	2.31E-20	9.79E-19
Slfn3	27.52	62.05	2.25	5.18E-08	6.46E-07
BC068281	12.77	28.75	2.25	2.80E-04	1.67E-03
Ctif	24.28	54.64	2.25	4.08E-05	2.97E-04
Kif3b	168.93	380.00	2.25	5.51E-07	5.83E-06
Keap1	146.80	330.19	2.25	3.93E-07	4.23E-06
Plod3	183.61	412.19	2.24	4.61E-11	8.47E-10
Relb	46.57	104.50	2.24	6.02E-08	7.44E-07
Slfn9	130.77	293.23	2.24	6.70E-04	3.62E-03
Dync1h1	1800.67	4035.07	2.24	1.59E-17	5.49E-16
Gm5506	200.42	448.73	2.24	1.22E-13	2.99E-12
Pvrl2	18.54	41.47	2.24	4.81E-03	1.92E-02
Atp7a	293.15	655.08	2.23	1.08E-18	3.94E-17
Mllt3	28.49	63.57	2.23	5.30E-06	4.73E-05
2810474O19Rik	177.09	395.05	2.23	6.68E-06	5.85E-05
Pak1	116.79	260.51	2.23	4.21E-14	1.07E-12
Usb1	45.60	101.64	2.23	3.57E-08	4.57E-07
Sipa1l1	60.65	134.99	2.23	3.89E-08	4.94E-07
Nup210	22.34	49.73	2.23	3.73E-06	3.43E-05
Irf5	282.85	627.83	2.22	2.95E-12	6.13E-11
Utrn	147.79	327.89	2.22	2.66E-14	6.86E-13
Usp42	29.02	64.32	2.22	2.71E-05	2.08E-04

Irf2	147.56	326.82	2.21	5.17E-14	1.30E-12
Sowahc	49.58	109.76	2.21	1.19E-08	1.63E-07
Anks1	72.16	159.62	2.21	1.03E-09	1.61E-08
Nrcam	64.96	143.64	2.21	3.63E-05	2.68E-04
Plekhm1	350.34	774.02	2.21	1.38E-11	2.65E-10
Vars	237.04	523.55	2.21	3.06E-09	4.50E-08
B3gnt2	171.45	378.35	2.21	3.13E-16	9.63E-15
Zbtb7a	284.85	626.56	2.20	1.64E-05	1.31E-04
Tlr6	19.75	43.44	2.20	1.64E-05	1.31E-04
Casp7	36.70	80.65	2.20	1.51E-06	1.49E-05
Scarf1	14.83	32.56	2.20	4.02E-05	2.94E-04
Vcpip1	151.24	331.83	2.19	1.46E-09	2.23E-08
Gpr108	102.89	225.46	2.19	2.49E-10	4.20E-09
AB124611	107.23	234.75	2.19	1.12E-07	1.33E-06
Atf6	240.81	526.76	2.19	3.17E-08	4.08E-07
Crkl	144.78	316.19	2.18	1.49E-10	2.58E-09
Wrn	79.08	172.51	2.18	8.12E-10	1.29E-08
Lair1	258.23	559.87	2.17	4.24E-13	9.83E-12
Nol10	41.74	90.37	2.16	1.37E-06	1.37E-05
Gpr126	17.12	37.04	2.16	1.14E-03	5.73E-03
9530082P21Rik	19.99	43.23	2.16	2.83E-05	2.16E-04
Tox4	143.84	310.88	2.16	4.72E-12	9.50E-11
Picalm	426.91	921.55	2.16	5.81E-17	1.93E-15
Gm15800	169.72	366.20	2.16	1.22E-07	1.44E-06
Fbxl18	13.23	28.53	2.16	7.17E-04	3.83E-03
S1pr2	155.01	334.00	2.15	4.58E-10	7.53E-09
Nt5c3	30.41	65.41	2.15	9.85E-05	6.59E-04
Tcp11l1	30.55	65.62	2.15	2.30E-05	1.79E-04
Ncln	133.48	286.08	2.14	3.82E-07	4.14E-06
Cyld	129.06	276.54	2.14	8.30E-10	1.31E-08
Galnt10	85.94	183.95	2.14	1.64E-09	2.50E-08
Tbc1d13	191.13	408.82	2.14	4.90E-12	9.84E-11
Inpp4a	115.89	247.58	2.14	7.16E-11	1.28E-09
Hif1a	312.18	666.86	2.14	1.72E-14	4.55E-13
Rasa4	273.54	584.26	2.14	1.71E-16	5.43E-15
Arid4a	122.55	261.66	2.14	6.10E-11	1.10E-09
Clip2	111.28	237.38	2.13	1.58E-04	1.01E-03
Srgap2	355.56	757.77	2.13	2.90E-16	9.00E-15
Mark4	41.54	88.22	2.12	8.87E-06	7.47E-05
Slc33a1	60.68	128.57	2.12	5.18E-09	7.42E-08
Megf8	155.05	328.35	2.12	8.66E-13	1.92E-11
Sertad3	11.79	24.96	2.12	2.68E-03	1.18E-02
Glipr2	8.50	17.97	2.12	5.00E-03	1.98E-02

Cd2ap	224.62	474.94	2.11	1.92E-13	4.58E-12
Nrp1	983.87	2080.15	2.11	4.19E-17	1.41E-15
Aig1	34.04	71.89	2.11	3.42E-06	3.17E-05
Tyr	8.69	18.35	2.11	1.43E-03	7.00E-03
Fn1	448.98	945.08	2.10	2.79E-04	1.67E-03
Twsg1	123.40	259.67	2.10	1.04E-10	1.82E-09
Ctsh	25.89	54.47	2.10	1.77E-05	1.41E-04
Kif1c	521.48	1096.96	2.10	6.76E-17	2.24E-15
Plec	2708.54	5695.89	2.10	1.66E-10	2.87E-09
Eif4e3	49.47	103.86	2.10	8.26E-07	8.52E-06
Pura	34.18	71.69	2.10	2.59E-05	1.99E-04
Rbms1	254.41	533.29	2.10	1.11E-11	2.15E-10
Klra2	21.03	43.96	2.09	4.30E-03	1.75E-02
Mitd1	18.89	39.49	2.09	4.60E-03	1.85E-02
Sik3	90.55	189.17	2.09	5.63E-09	8.02E-08
Etnk1	175.34	366.09	2.09	1.60E-13	3.87E-12
Map4	518.54	1081.86	2.09	1.66E-14	4.40E-13
Klf6	740.96	1541.11	2.08	4.74E-15	1.31E-13
Plscr3	53.15	110.52	2.08	1.30E-03	6.44E-03
Pik3ap1	520.45	1082.28	2.08	2.27E-12	4.81E-11
Ktn1	81.59	169.59	2.08	1.35E-08	1.83E-07
Gmppb	32.32	67.09	2.08	2.32E-07	2.60E-06
Psen1	183.24	380.25	2.08	2.53E-13	5.96E-12
Foxn3	53.26	110.50	2.07	1.35E-06	1.35E-05
Gatm	229.66	475.54	2.07	3.79E-13	8.85E-12
Smarca4	404.74	837.97	2.07	6.97E-13	1.57E-11
Runx3	17.34	35.89	2.07	3.89E-03	1.61E-02
Top3a	49.50	102.44	2.07	1.20E-05	9.83E-05
Unc45a	31.71	65.55	2.07	5.01E-03	1.99E-02
Sh3pxd2b	433.39	894.62	2.06	1.54E-15	4.50E-14
Eif4g3	425.43	877.68	2.06	1.08E-12	2.38E-11
Tceanc2	44.50	91.72	2.06	7.44E-07	7.69E-06
Mllt6	164.40	338.57	2.06	2.21E-06	2.12E-05
Gtf3c5	48.55	99.83	2.06	6.46E-07	6.74E-06
Tbxas1	143.36	294.73	2.06	5.33E-13	1.22E-11
Adam15	582.90	1198.13	2.06	7.85E-14	1.96E-12
Alkbh5	300.87	618.34	2.06	2.70E-12	5.65E-11
Klf3	45.85	94.15	2.05	4.05E-06	3.69E-05
Tbc1d22b	44.17	90.61	2.05	1.83E-06	1.79E-05
Plekhg2	27.90	57.22	2.05	1.55E-05	1.24E-04
Ap1b1	219.45	449.91	2.05	1.98E-12	4.21E-11
Plcl2	81.52	167.08	2.05	2.34E-08	3.07E-07
E330023G01Rik	8.99	18.42	2.05	3.35E-03	1.43E-02

Nbas	53.65	109.91	2.05	3.46E-08	4.43E-07
Gpr146	68.17	139.64	2.05	6.96E-10	1.12E-08
Plekhn1	35.45	72.45	2.04	3.11E-05	2.34E-04
Tnrc18	239.57	489.48	2.04	3.75E-06	3.45E-05
Papss2	24.11	49.20	2.04	3.11E-04	1.84E-03
Arf3	404.29	824.36	2.04	3.08E-14	7.91E-13
Fbrs	130.11	265.09	2.04	1.52E-09	2.31E-08
Zfhx3	261.87	533.45	2.04	3.43E-04	2.00E-03
Fam222b	53.61	109.06	2.03	8.90E-06	7.49E-05
Rps6ka4	92.22	187.58	2.03	6.49E-06	5.70E-05
Fam207a	38.55	78.38	2.03	2.80E-05	2.14E-04
Fryl	241.96	491.40	2.03	1.14E-12	2.48E-11
Lrp5	95.71	194.14	2.03	1.99E-03	9.28E-03
Etv3	327.95	664.82	2.03	7.89E-13	1.77E-11
Ankrd11	340.69	690.46	2.03	7.52E-15	2.06E-13
Hmbox1	56.59	114.60	2.03	1.77E-07	2.02E-06
Lfng	108.36	218.78	2.02	3.39E-06	3.15E-05
Wdfy2	91.12	183.86	2.02	4.46E-08	5.62E-07
Nlrp3	193.57	390.59	2.02	1.01E-07	1.21E-06
Pfkl	254.90	514.20	2.02	8.35E-09	1.17E-07
Yif1b	40.98	82.56	2.01	3.10E-05	2.34E-04
Slc7a8	255.30	513.44	2.01	2.13E-08	2.81E-07
Ltbr	106.10	212.77	2.01	1.73E-10	2.98E-09
Tiam1	71.26	142.73	2.00	1.27E-07	1.49E-06
Aff1	201.66	403.72	2.00	2.97E-11	5.57E-10
Mvb12a	55.73	111.48	2.00	6.81E-05	4.77E-04
Snx20	50.99	101.98	2.00	7.52E-06	6.48E-05
**Down-regulated**					
Cd28	61.41	2.22	0.04	1.59E-26	9.75E-25
Ypel2	37.72	1.96	0.05	1.12E-18	4.05E-17
S1pr1	162.17	9.20	0.06	8.94E-62	2.10E-59
Ypel3	40.83	2.62	0.06	4.47E-15	1.25E-13
Cxcr4	317.38	21.58	0.07	2.42E-86	9.79E-84
F630028O10Rik	66.54	6.04	0.09	9.79E-22	4.50E-20
Slco4a1	126.14	11.70	0.09	1.14E-36	1.26E-34
Pdcd4	38.65	3.64	0.09	5.79E-13	1.32E-11
Cxcl1	17.40	1.69	0.10	1.41E-14	3.75E-13
Cxcl2	38.26	4.36	0.11	4.74E-20	1.95E-18
Cpeb1	18.78	2.18	0.12	1.66E-06	1.63E-05
Ube2t	19.47	2.36	0.12	4.93E-05	3.53E-04
Acsl3	78.91	9.84	0.12	1.95E-23	9.97E-22
Zfand4	14.17	1.78	0.13	4.49E-04	2.53E-03

Lhfpl2	472.46	61.83	0.13	1.65E-67	4.63E-65
Chst10	122.29	16.54	0.14	8.16E-30	6.35E-28
Cgnl1	42.06	5.71	0.14	2.19E-19	8.51E-18
Idh1	483.48	68.80	0.14	7.23E-50	1.14E-47
Sla	60.59	8.64	0.14	7.11E-21	3.14E-19
Slc6a12	34.77	5.10	0.15	9.74E-11	1.71E-09
Dio2	13.98	2.09	0.15	6.07E-06	5.37E-05
Dusp1	74.38	11.19	0.15	3.60E-23	1.82E-21
Pcyt1b	18.54	3.04	0.16	1.40E-05	1.13E-04
Mdm1	52.82	8.98	0.17	8.00E-13	1.79E-11
Prokr1	17.01	2.89	0.17	1.55E-06	1.53E-05
Cx3cr1	594.79	101.28	0.17	4.31E-27	2.74E-25
Utp14b	47.28	8.09	0.17	1.22E-13	2.98E-12
Ankrd37	18.72	3.28	0.18	1.90E-04	1.19E-03
Snhg5	33.11	5.86	0.18	2.62E-07	2.92E-06
St6gal1	133.32	24.05	0.18	8.87E-25	4.98E-23
Cdca7	27.63	5.13	0.19	7.16E-08	8.79E-07
Slc26a11	23.43	4.47	0.19	6.65E-06	5.83E-05
Rnf150	179.35	34.21	0.19	6.19E-26	3.70E-24
Gpr183	66.97	12.92	0.19	3.11E-15	8.81E-14
Aph1c	52.51	10.19	0.19	1.71E-13	4.12E-12
Nuak1	16.70	3.25	0.19	7.48E-06	6.45E-05
Prickle2	14.41	2.81	0.19	4.41E-05	3.19E-04
Dusp4	207.41	40.81	0.20	6.07E-31	4.80E-29
Oit3	17.26	3.46	0.20	1.83E-04	1.15E-03
Depdc7	13.70	2.76	0.20	3.98E-04	2.28E-03
Ccl3	67.50	13.60	0.20	7.15E-12	1.41E-10
Ptpn22	61.29	12.44	0.20	1.41E-13	3.43E-12
Sgk1	134.82	27.47	0.20	1.00E-27	6.72E-26
Ccsap	37.33	7.61	0.20	3.04E-09	4.49E-08
Dhrs3	619.55	126.76	0.20	5.69E-53	9.75E-51
Arhgap33	18.96	3.92	0.21	8.09E-04	4.26E-03
Rhob	107.64	22.70	0.21	1.64E-23	8.46E-22
Rgs18	75.09	16.16	0.22	9.77E-14	2.41E-12
Mctp1	56.40	12.18	0.22	1.80E-11	3.43E-10
P2ry1	34.98	7.61	0.22	3.48E-07	3.79E-06
Lyl1	96.11	21.09	0.22	9.95E-13	2.19E-11
Zfp692	14.77	3.24	0.22	3.46E-04	2.01E-03
Dennd2c	48.28	10.95	0.23	9.85E-11	1.73E-09
Apbb3	17.08	3.88	0.23	2.64E-04	1.59E-03
Fam105a	161.74	36.74	0.23	9.81E-23	4.87E-21
Tcp11l2	31.40	7.17	0.23	6.64E-05	4.67E-04
Tarsl2	18.91	4.40	0.23	4.71E-04	2.64E-03

Depdc1a	33.11	7.78	0.24	2.43E-05	1.88E-04
C5ar1	473.43	112.89	0.24	7.90E-43	1.02E-40
Deptor	17.27	4.13	0.24	3.28E-04	1.92E-03
Cenpk	13.31	3.20	0.24	3.10E-03	1.33E-02
Arhgap22	58.36	14.25	0.24	1.11E-11	2.14E-10
Ica1	41.54	10.19	0.25	3.60E-08	4.59E-07
Adrb2	44.62	10.96	0.25	3.00E-09	4.44E-08
1810011H11Rik	52.40	13.03	0.25	3.74E-12	7.66E-11
Stap1	188.30	46.87	0.25	3.02E-29	2.28E-27
Blnk	113.59	28.38	0.25	8.38E-10	1.32E-08
Oard1	12.98	3.27	0.25	1.68E-03	8.00E-03
Plscr4	66.87	16.92	0.25	1.91E-12	4.08E-11
Bcl7a	19.65	4.98	0.25	3.58E-03	1.51E-02
Smad3	21.81	5.58	0.26	1.38E-07	1.61E-06
Cd24a	44.79	11.49	0.26	1.16E-09	1.78E-08
Fgfr1	8.62	2.26	0.26	5.15E-03	2.03E-02
Trp53inp1	44.70	11.93	0.27	2.41E-06	2.29E-05
Tbc1d4	141.70	38.07	0.27	4.17E-14	1.06E-12
Rab4a	16.79	4.52	0.27	2.34E-04	1.43E-03
Tacc2	20.71	5.65	0.27	5.81E-06	5.16E-05
Kctd12b	93.34	25.55	0.27	1.79E-15	5.20E-14
Cd300lb	369.96	101.35	0.27	2.68E-34	2.69E-32
Mamdc2	31.96	8.77	0.27	1.67E-05	1.33E-04
Engase	20.04	5.51	0.27	2.81E-03	1.23E-02
Rasgrp3	222.06	61.47	0.28	4.04E-25	2.34E-23
Osgin1	26.43	7.39	0.28	5.05E-05	3.61E-04
Rnf113a2	24.88	7.00	0.28	1.00E-03	5.14E-03
Add3	145.10	41.17	0.28	3.11E-16	9.58E-15
Dab2	1585.79	450.73	0.28	2.19E-39	2.58E-37
Sirpb1a	21.32	6.07	0.28	8.81E-04	4.57E-03
Irs2	21.45	6.13	0.29	3.36E-03	1.43E-02
Bhlhe40	111.59	32.06	0.29	1.53E-14	4.06E-13
Rragd	16.03	4.61	0.29	1.28E-03	6.37E-03
Neil3	68.11	19.80	0.29	1.02E-09	1.58E-08
Recql4	22.23	6.47	0.29	3.22E-03	1.38E-02
Ldlrad3	50.94	14.83	0.29	9.07E-11	1.60E-09
Rapgef5	110.64	32.37	0.29	1.31E-18	4.75E-17
Zfp788	19.31	5.65	0.29	4.54E-03	1.83E-02
St18	51.05	15.00	0.29	1.55E-11	2.97E-10
Eif4a2	282.02	82.86	0.29	7.72E-21	3.39E-19
Rhov	25.81	7.59	0.29	1.28E-04	8.39E-04
Kif18a	28.15	8.28	0.29	9.36E-05	6.29E-04
Rpain	17.22	5.10	0.30	1.30E-04	8.50E-04

Dusp9	11.87	3.53	0.30	4.30E-03	1.75E-02
Arhgap18	177.61	53.10	0.30	2.31E-18	8.24E-17
Zfp472	15.81	4.73	0.30	1.46E-03	7.14E-03
Cox20	21.93	6.58	0.30	2.18E-03	9.99E-03
Hal	525.45	157.70	0.30	6.33E-25	3.62E-23
Reps2	90.14	27.08	0.30	3.01E-11	5.64E-10
Dfna5	21.50	6.46	0.30	1.32E-04	8.61E-04
Tmem64	65.17	19.62	0.30	3.20E-08	4.12E-07
Pgm2l1	247.52	74.63	0.30	1.18E-19	4.71E-18
Cbr3	26.96	8.14	0.30	9.70E-05	6.51E-04
Cd244	8.69	2.62	0.30	2.20E-03	1.01E-02
Ankle1	31.09	9.39	0.30	2.36E-05	1.83E-04
Tmem154	84.27	25.49	0.30	8.91E-10	1.39E-08
Per3	34.06	10.32	0.30	6.38E-06	5.61E-05
Daglb	231.70	70.60	0.30	1.24E-22	6.07E-21
Rgl2	175.43	53.53	0.31	1.77E-12	3.78E-11
Ccl9	741.55	226.55	0.31	9.83E-17	3.22E-15
Ndst1	101.81	31.17	0.31	9.45E-12	1.83E-10
Acat2	40.99	12.59	0.31	8.40E-10	1.32E-08
Sesn1	132.75	40.99	0.31	7.61E-15	2.08E-13
Cd300a	187.75	58.01	0.31	1.40E-16	4.49E-15
Il1rl1	19.18	5.95	0.31	3.73E-03	1.56E-02
Mtbp	29.49	9.14	0.31	3.29E-03	1.40E-02
Bhlhe41	429.09	133.39	0.31	1.01E-25	5.95E-24
Gins3	25.85	8.04	0.31	3.72E-03	1.56E-02
Adss	373.08	116.64	0.31	6.33E-14	1.58E-12
Ier2	52.27	16.41	0.31	1.06E-05	8.77E-05
Gcnt1	314.40	98.93	0.31	2.44E-19	9.44E-18
1700066M21Rik	14.51	4.58	0.32	3.33E-04	1.95E-03
Ccdc142	20.02	6.32	0.32	1.73E-03	8.19E-03
1110038B12Rik	23.75	7.52	0.32	7.57E-04	4.02E-03
Wrb	40.28	12.75	0.32	5.76E-04	3.16E-03
Zfp101	17.21	5.48	0.32	3.68E-03	1.54E-02
Susd3	56.16	17.90	0.32	2.85E-09	4.23E-08
Stk17b	173.16	55.24	0.32	1.18E-15	3.46E-14
Meaf6	21.69	6.94	0.32	2.63E-03	1.16E-02
Runx2	56.36	18.04	0.32	8.13E-12	1.59E-10
Ckap2	66.69	21.44	0.32	2.67E-08	3.48E-07
Scd1	273.54	88.17	0.32	1.92E-11	3.63E-10
Peli2	69.10	22.29	0.32	7.49E-10	1.20E-08
Lpar6	64.98	21.05	0.32	9.48E-04	4.87E-03
2410006H16Rik	13.95	4.52	0.32	2.19E-03	1.01E-02
Orc6	29.15	9.47	0.32	1.48E-03	7.17E-03

Zfp157	26.36	8.57	0.33	2.45E-03	1.10E-02
Cd14	757.97	246.83	0.33	1.99E-32	1.64E-30
Cdc7	33.03	10.77	0.33	7.39E-04	3.93E-03
Esco2	55.12	17.98	0.33	1.25E-08	1.71E-07
Cenpl	34.08	11.12	0.33	6.54E-04	3.54E-03
Zfp503	30.25	9.87	0.33	1.35E-04	8.79E-04
Apoc2	23.18	7.57	0.33	2.94E-03	1.28E-02
Atg9b	13.05	4.28	0.33	2.77E-03	1.22E-02
Tipin	53.17	17.49	0.33	1.19E-06	1.20E-05
Tmem26	32.32	10.65	0.33	4.59E-03	1.85E-02
Polr2k	32.52	10.74	0.33	3.74E-05	2.76E-04
Cd163	34.60	11.45	0.33	1.13E-05	9.31E-05
Sc4mol	172.46	57.06	0.33	8.47E-19	3.13E-17
Cdca7l	67.90	22.56	0.33	6.07E-09	8.61E-08
Slirp	26.29	8.75	0.33	1.34E-03	6.61E-03
Eno2	28.88	9.67	0.33	3.57E-04	2.07E-03
Idi1	137.25	46.16	0.34	9.57E-11	1.68E-09
Lpar5	25.11	8.46	0.34	7.67E-04	4.07E-03
Lyz1	2868.18	966.84	0.34	3.97E-24	2.12E-22
Ccl6	118.58	40.23	0.34	2.17E-11	4.10E-10
Tox2	20.67	7.02	0.34	2.45E-05	1.90E-04
Lyzl4	14.77	5.02	0.34	4.63E-03	1.86E-02
Wdr67	105.90	36.40	0.34	3.65E-10	6.07E-09
Fam107b	104.28	35.88	0.34	6.13E-11	1.11E-09
Ndnl2	63.61	22.21	0.35	9.19E-08	1.11E-06
Plekhh2	39.27	13.72	0.35	3.05E-06	2.85E-05
Sqle	135.73	47.46	0.35	1.36E-19	5.38E-18
Cdkn2aip	20.39	7.13	0.35	1.08E-04	7.15E-04
Nanp	22.50	7.87	0.35	4.42E-03	1.79E-02
Cfl2	26.80	9.38	0.35	4.46E-03	1.81E-02
Marveld1	101.12	35.46	0.35	4.21E-11	7.74E-10
Cnnm2	24.32	8.53	0.35	1.40E-05	1.13E-04
Lonrf3	306.22	107.42	0.35	4.16E-15	1.16E-13
2610001J05Rik	111.62	39.36	0.35	1.10E-09	1.70E-08
Il16	36.18	12.78	0.35	1.64E-04	1.05E-03
Ddx26b	104.86	37.12	0.35	8.36E-11	1.49E-09
Sncaip	31.88	11.31	0.35	3.36E-05	2.51E-04
Prmt3	47.91	17.01	0.35	3.71E-08	4.73E-07
Haus3	43.99	15.63	0.36	1.36E-04	8.86E-04
Uqcrb	111.43	39.79	0.36	2.55E-09	3.80E-08
Ypel5	125.00	44.78	0.36	1.27E-09	1.94E-08
Abcd2	68.14	24.46	0.36	2.39E-13	5.66E-12
Rad51ap1	28.22	10.18	0.36	4.80E-03	1.92E-02

Ing4	66.97	24.19	0.36	7.89E-09	1.11E-07
Mfap3l	38.80	14.02	0.36	3.73E-03	1.56E-02
Sc5d	90.16	32.79	0.36	7.20E-12	1.42E-10
Pank1	18.57	6.77	0.36	4.07E-03	1.67E-02
Appl2	150.70	54.96	0.36	1.44E-12	3.09E-11
Elovl6	73.42	26.82	0.37	4.74E-09	6.84E-08
Fgd4	259.99	95.12	0.37	7.69E-10	1.22E-08
Igf1	1845.83	676.62	0.37	2.60E-19	1.00E-17
Nostrin	63.02	23.10	0.37	6.41E-08	7.90E-07
Mrps6	27.97	10.30	0.37	4.14E-07	4.45E-06
Cep70	51.58	19.00	0.37	7.00E-06	6.09E-05
Slc5a3	77.33	28.54	0.37	9.07E-09	1.26E-07
Cyp51	127.40	47.05	0.37	1.93E-08	2.57E-07
Zcchc11	102.62	37.97	0.37	9.00E-11	1.59E-09
Tob1	16.32	6.05	0.37	1.38E-03	6.79E-03
Clec4a2	58.88	21.92	0.37	2.90E-04	1.73E-03
Pcp4l1	127.01	47.32	0.37	1.90E-14	4.99E-13
Ccdc111	24.54	9.16	0.37	3.21E-03	1.38E-02
Dennd4a	130.81	48.93	0.37	3.79E-11	7.00E-10
Irf2bp2	471.06	176.36	0.37	5.81E-18	2.03E-16
Rcsd1	71.08	26.66	0.38	8.59E-11	1.53E-09
2900026A02Rik	111.73	41.91	0.38	2.03E-10	3.46E-09
Aldh6a1	29.84	11.20	0.38	1.28E-03	6.38E-03
Cd97	142.15	53.37	0.38	1.88E-13	4.49E-12
Prkag2	53.26	20.01	0.38	1.54E-03	7.41E-03
2610044O15Rik8	12.82	4.82	0.38	4.21E-03	1.72E-02
Snx7	71.90	27.11	0.38	4.56E-09	6.60E-08
Cdk17	66.96	25.26	0.38	8.08E-06	6.92E-05
Prkra	38.71	14.64	0.38	3.70E-05	2.73E-04
L2hgdh	11.92	4.51	0.38	2.70E-03	1.19E-02
Mpzl1	13.86	5.24	0.38	2.61E-03	1.16E-02
Csgalnact2	144.42	54.71	0.38	1.59E-10	2.76E-09
Kctd7	25.58	9.69	0.38	9.25E-04	4.77E-03
Fgfr1op	40.90	15.52	0.38	2.00E-03	9.31E-03
Sorbs3	24.56	9.34	0.38	1.86E-04	1.16E-03
Rgs10	165.96	63.26	0.38	1.38E-10	2.41E-09
D4Wsu53e	213.77	81.55	0.38	1.73E-12	3.72E-11
Pot1a	32.30	12.34	0.38	6.48E-04	3.51E-03
Clec4d	518.13	197.95	0.38	2.50E-14	6.48E-13
ENSMUSG00000064350	37.60	14.38	0.38	1.03E-03	5.25E-03
Mxd4	30.31	11.59	0.38	4.05E-04	2.31E-03
Rhno1	34.12	13.05	0.38	4.10E-04	2.34E-03
Zfp715	57.71	22.14	0.38	8.60E-08	1.04E-06

Ankra2	20.63	7.91	0.38	2.63E-03	1.16E-02
Lrrc57	23.45	9.00	0.38	2.99E-03	1.29E-02
Psenen	79.68	30.60	0.38	9.11E-06	7.64E-05
Sh3bgrl2	111.66	42.90	0.38	8.35E-13	1.86E-11
Arap3	258.41	99.42	0.38	9.26E-17	3.04E-15
Pitpnc1	64.98	25.02	0.39	5.54E-08	6.86E-07
4632434I11Rik	25.87	10.00	0.39	4.06E-03	1.67E-02
Dusp6	255.42	98.83	0.39	9.18E-19	3.35E-17
Ppcdc	23.88	9.25	0.39	2.11E-03	9.75E-03
Rmi1	69.07	26.76	0.39	1.35E-03	6.66E-03
Slbp	129.93	50.43	0.39	9.09E-11	1.61E-09
Mfsd7b	102.34	39.79	0.39	3.33E-11	6.19E-10
Mzt1	63.73	24.80	0.39	2.61E-07	2.90E-06
Snx24	59.87	23.33	0.39	1.03E-04	6.84E-04
Tmem65	83.86	32.84	0.39	1.65E-07	1.90E-06
Dtx3	36.35	14.30	0.39	8.61E-05	5.87E-04
Nasp	119.17	46.92	0.39	1.59E-08	2.13E-07
Ifngr1	318.16	125.28	0.39	8.55E-19	3.14E-17
Gpr137b	127.32	50.14	0.39	1.94E-09	2.92E-08
Mis18bp1	125.49	49.49	0.39	1.39E-06	1.38E-05
Tlr8	242.53	95.68	0.39	7.76E-08	9.50E-07
Tbc1d15	133.81	52.81	0.39	2.56E-07	2.86E-06
Wwp1	496.88	196.52	0.40	3.23E-16	9.87E-15
Orc5	31.21	12.37	0.40	1.58E-03	7.62E-03
Tmem199	28.16	11.19	0.40	2.88E-04	1.72E-03
Pole2	23.44	9.32	0.40	4.95E-04	2.76E-03
Ndufb3	42.72	17.03	0.40	1.76E-03	8.31E-03
Slc2a3	29.05	11.58	0.40	3.63E-04	2.10E-03
Sephs2	167.56	67.01	0.40	3.79E-11	7.00E-10
Cpe	20.70	8.29	0.40	4.40E-03	1.79E-02
Msh5	43.56	17.47	0.40	1.80E-04	1.14E-03
Ttc33	45.38	18.25	0.40	2.99E-03	1.29E-02
Foxred2	35.54	14.29	0.40	2.81E-03	1.23E-02
Zfand6	115.17	46.43	0.40	7.88E-08	9.64E-07
Arhgef39	31.47	12.69	0.40	1.90E-05	1.50E-04
Neat1	930.50	375.39	0.40	2.44E-04	1.48E-03
Creg2	39.34	15.91	0.40	4.45E-04	2.51E-03
Cytip	23.48	9.51	0.40	7.08E-05	4.94E-04
Prpf39	91.05	36.94	0.41	1.70E-08	2.27E-07
Bub1	151.18	61.45	0.41	4.68E-10	7.68E-09
Hells	76.74	31.20	0.41	6.04E-07	6.33E-06
Timm8a1	31.48	12.81	0.41	4.22E-05	3.06E-04
Dtl	73.91	30.10	0.41	7.38E-05	5.11E-04

Atp5sl	30.65	12.50	0.41	3.36E-03	1.43E-02
Cd33	48.99	19.97	0.41	2.35E-03	1.06E-02
Rnasel	43.45	17.75	0.41	5.59E-05	3.97E-04
Gngt2	48.83	19.96	0.41	3.89E-07	4.19E-06
Arl11	150.96	61.75	0.41	5.89E-11	1.07E-09
Cdc6	49.58	20.30	0.41	1.94E-03	9.03E-03
Pcyt2	70.09	28.70	0.41	6.89E-08	8.47E-07
Prim1	45.09	18.47	0.41	8.92E-04	4.62E-03
Cirbp	28.12	11.52	0.41	8.97E-04	4.64E-03
Polr3g	19.05	7.81	0.41	2.96E-03	1.28E-02
Gpcpd1	83.67	34.36	0.41	4.85E-08	6.06E-07
Lyrm5	16.52	6.80	0.41	4.05E-04	2.31E-03
Gm17296	21.62	8.92	0.41	4.42E-04	2.49E-03
Cdk2	105.43	43.55	0.41	8.79E-09	1.23E-07
Alox5	105.73	43.70	0.41	3.45E-06	3.19E-05
Ska2	39.23	16.22	0.41	8.70E-04	4.53E-03
Fam20c	291.88	121.01	0.41	8.19E-08	9.97E-07
Crot	73.41	30.48	0.42	5.07E-06	4.54E-05
Dbi	105.89	44.07	0.42	1.33E-07	1.56E-06
Prkar2b	54.44	22.68	0.42	5.53E-04	3.05E-03
Magoh	37.24	15.53	0.42	1.29E-03	6.41E-03
2700094K13Rik	33.46	14.02	0.42	5.65E-04	3.11E-03
Arl4c	404.00	169.41	0.42	6.90E-19	2.58E-17
Hnrnph1	449.25	188.65	0.42	1.77E-10	3.04E-09
Aph1b	29.69	12.51	0.42	3.77E-05	2.78E-04
Scai	33.44	14.09	0.42	5.19E-05	3.71E-04
Mmd	68.84	29.02	0.42	5.19E-06	4.63E-05
Nav2	281.66	118.76	0.42	8.67E-12	1.69E-10
Mrpl33	55.72	23.52	0.42	3.83E-05	2.81E-04
Snx30	232.20	98.02	0.42	1.94E-10	3.31E-09
Pilra	35.07	14.82	0.42	1.62E-05	1.30E-04
Mad2l1	106.83	45.21	0.42	1.75E-07	2.00E-06
Tlr7	381.93	162.08	0.42	1.99E-06	1.92E-05
Tmem71	31.94	13.61	0.43	4.33E-04	2.45E-03
Zfp825	25.32	10.81	0.43	4.90E-04	2.74E-03
Tsc22d3	38.26	16.35	0.43	3.72E-05	2.75E-04
Mmp12	1299.34	555.42	0.43	1.97E-12	4.19E-11
Msh6	125.32	53.60	0.43	5.47E-09	7.81E-08
Gpr162	14.13	6.06	0.43	1.21E-03	6.07E-03
Gdpd1	83.98	36.03	0.43	5.47E-08	6.80E-07
Tpm1	109.98	47.19	0.43	4.95E-09	7.13E-08
Tom1l1	17.45	7.50	0.43	4.68E-03	1.88E-02
Atp2b4	55.66	23.99	0.43	8.54E-05	5.82E-04

B230120H23Rik	201.99	87.11	0.43	2.75E-12	5.76E-11
Fmn1	302.96	131.06	0.43	7.40E-06	6.38E-05
Pbk	65.78	28.46	0.43	3.34E-07	3.65E-06
Ska1	15.54	6.72	0.43	4.54E-03	1.83E-02
Akap8l	47.47	20.55	0.43	7.03E-04	3.76E-03
Trmt10c	29.91	12.97	0.43	1.73E-03	8.21E-03
1810022K09Rik	49.76	21.62	0.43	6.99E-06	6.08E-05
Anxa1	1796.83	780.82	0.43	1.10E-20	4.79E-19
Fignl1	83.34	36.27	0.44	1.02E-08	1.41E-07
Id2	189.35	82.45	0.44	8.40E-10	1.32E-08
Tmem184c	94.15	41.13	0.44	2.14E-07	2.42E-06
1810058I24Rik	93.80	41.13	0.44	4.36E-06	3.94E-05
Smim11	19.80	8.70	0.44	1.39E-03	6.81E-03
Pigx	26.64	11.73	0.44	4.01E-03	1.65E-02
Trappc1	65.40	28.92	0.44	7.15E-05	4.97E-04
Srsf5	398.42	176.31	0.44	6.97E-07	7.22E-06
Dhcr7	95.54	42.30	0.44	2.11E-07	2.39E-06
Pttg1	54.02	23.95	0.44	1.08E-04	7.17E-04
Tdp2	37.85	16.79	0.44	2.01E-03	9.32E-03
Cd300ld	167.31	74.27	0.44	9.83E-10	1.53E-08
Plk3	28.23	12.54	0.44	1.16E-03	5.84E-03
Zdhhc14	53.77	23.89	0.44	7.75E-05	5.34E-04
Sntb2	113.28	50.41	0.45	3.39E-12	7.01E-11
Chek1	27.32	12.18	0.45	1.21E-03	6.06E-03
Gclc	91.10	40.74	0.45	1.84E-07	2.09E-06
Thoc1	69.08	30.91	0.45	2.53E-04	1.53E-03
Dhx40	75.24	33.70	0.45	1.55E-08	2.08E-07
Dvl2	41.32	18.53	0.45	2.54E-04	1.54E-03
Pald1	59.22	26.60	0.45	2.10E-04	1.30E-03
Fam199x	51.32	23.13	0.45	6.35E-04	3.45E-03
Rrm2	278.79	125.81	0.45	5.37E-13	1.23E-11
Tceb1	84.49	38.17	0.45	8.22E-06	6.99E-05
Srsf6	289.19	130.89	0.45	2.62E-10	4.41E-09
Dnajc9	84.78	38.41	0.45	3.41E-06	3.16E-05
Arhgap24	65.50	29.69	0.45	1.41E-06	1.40E-05
Med21	26.36	11.98	0.45	2.25E-03	1.03E-02
Glt8d1	47.91	21.78	0.45	2.70E-03	1.19E-02
Srgap3	53.64	24.40	0.45	1.37E-04	8.92E-04
Etv1	101.79	46.40	0.46	8.96E-07	9.20E-06
Hagh	26.88	12.26	0.46	1.59E-03	7.63E-03
Cdkn2aipnl	119.51	54.49	0.46	3.89E-06	3.56E-05
Sgsh	70.81	32.35	0.46	1.23E-06	1.23E-05
Dimt1	20.36	9.31	0.46	3.35E-03	1.42E-02

Naa20	119.21	54.52	0.46	1.85E-05	1.46E-04
Xylt2	290.98	133.09	0.46	1.19E-10	2.07E-09
Cep72	14.82	6.78	0.46	3.65E-03	1.53E-02
Imp3	43.44	19.88	0.46	1.85E-04	1.16E-03
Brca2	80.35	36.85	0.46	6.83E-04	3.68E-03
Prpsap2	39.24	18.00	0.46	1.34E-04	8.72E-04
Fdps	193.39	88.77	0.46	5.76E-11	1.05E-09
Ndufc2	41.62	19.12	0.46	6.28E-04	3.42E-03
Cbx3	214.27	98.46	0.46	2.22E-09	3.32E-08
Umps	62.89	28.99	0.46	3.98E-05	2.92E-04
Ccp110	39.04	18.01	0.46	1.88E-04	1.17E-03
Insig1	241.53	111.45	0.46	2.50E-13	5.91E-12
Hexim1	88.23	40.75	0.46	1.42E-08	1.93E-07
Pcnp	179.30	82.83	0.46	1.15E-06	1.17E-05
Nop10	51.41	23.83	0.46	2.41E-06	2.29E-05
Sh3pxd2a	91.96	42.74	0.46	1.83E-07	2.08E-06
Kcnj2	272.46	126.73	0.47	5.58E-10	9.06E-09
Depdc1b	36.04	16.77	0.47	8.67E-04	4.52E-03
Hsd17b7	54.18	25.25	0.47	8.92E-04	4.62E-03
Tsc22d1	61.14	28.50	0.47	1.21E-03	6.06E-03
Myc	80.37	37.47	0.47	4.80E-07	5.12E-06
Rfc4	24.89	11.61	0.47	8.78E-04	4.56E-03
Abca5	44.03	20.56	0.47	1.86E-04	1.16E-03
Slc7a6	48.26	22.58	0.47	2.20E-03	1.01E-02
4930422G04Rik	68.11	31.89	0.47	1.41E-05	1.14E-04
Cd84	993.23	465.08	0.47	4.92E-15	1.36E-13
ENSMUSG00000064356	523.80	245.30	0.47	4.40E-04	2.49E-03
Nfkbid	38.72	18.14	0.47	4.67E-03	1.87E-02
Parp2	57.41	26.94	0.47	2.24E-03	1.02E-02
Mical1	157.00	73.75	0.47	5.80E-07	6.08E-06
Gmfg	129.13	60.68	0.47	6.97E-07	7.22E-06
Flcn	101.05	47.52	0.47	8.67E-06	7.34E-05
Mcts1	44.35	20.87	0.47	2.41E-04	1.47E-03
Akip1	33.95	15.98	0.47	6.90E-04	3.71E-03
Tef	127.09	59.84	0.47	4.44E-07	4.75E-06
Ank	213.35	100.57	0.47	1.81E-07	2.07E-06
1190002N15Rik	76.10	35.98	0.47	6.92E-06	6.04E-05
Apbb1ip	479.14	226.57	0.47	5.32E-13	1.22E-11
Gmnn	78.83	37.29	0.47	1.40E-04	9.07E-04
Dock5	153.29	72.56	0.47	9.00E-09	1.25E-07
Kif21b	164.63	77.97	0.47	7.94E-08	9.70E-07
Azin1	414.18	196.34	0.47	5.53E-08	6.86E-07
Rabggtb	73.78	35.02	0.47	1.77E-04	1.12E-03

Mettl7a1	50.97	24.22	0.48	7.24E-04	3.87E-03
Trim59	107.66	51.36	0.48	2.39E-07	2.67E-06
4930579G24Rik	30.63	14.63	0.48	3.42E-05	2.55E-04
Chek2	43.80	20.92	0.48	3.66E-04	2.12E-03
Klf2	68.78	32.88	0.48	3.39E-06	3.15E-05
Cenpo	37.68	18.02	0.48	5.03E-03	1.99E-02
Ivns1abp	270.01	129.30	0.48	4.24E-09	6.16E-08
Supt20	62.97	30.16	0.48	9.30E-04	4.79E-03
Ttyh2	170.41	81.62	0.48	3.51E-11	6.51E-10
Fam96a	112.84	54.07	0.48	1.47E-05	1.19E-04
Mid1ip1	225.71	108.19	0.48	6.80E-07	7.06E-06
Bora	25.43	12.21	0.48	4.54E-03	1.83E-02
Slc9a5	23.14	11.11	0.48	1.55E-03	7.46E-03
Ubl5	83.48	40.09	0.48	3.94E-07	4.24E-06
Ssbp1	36.55	17.57	0.48	1.60E-03	7.66E-03
Apobec1	655.31	315.28	0.48	1.11E-12	2.42E-11
Naa50	405.62	195.32	0.48	8.93E-05	6.05E-04
Zfp704	91.96	44.37	0.48	3.91E-08	4.96E-07
Abhd10	21.82	10.54	0.48	1.86E-03	8.71E-03
Fsd1l	64.00	30.92	0.48	2.28E-03	1.03E-02
Eif2c4	56.42	27.27	0.48	3.91E-03	1.62E-02
Zbtb8os	41.16	19.89	0.48	2.12E-03	9.76E-03
Cenpa	71.37	34.51	0.48	6.27E-05	4.43E-04
Ncapg	113.52	54.89	0.48	2.58E-05	1.98E-04
Glipr1	382.41	185.10	0.48	1.53E-12	3.29E-11
Rps27l	87.12	42.21	0.48	9.73E-06	8.12E-05
Mrps21	49.44	23.95	0.48	1.07E-03	5.42E-03
Hmgcs1	238.86	115.84	0.48	2.63E-07	2.93E-06
Ndufa4	126.67	61.47	0.49	4.19E-08	5.30E-07
Hsf2	49.57	24.06	0.49	1.31E-03	6.50E-03
Alg10b	157.39	76.41	0.49	8.64E-08	1.05E-06
Mafb	493.13	239.45	0.49	4.49E-10	7.39E-09
Sgms2	51.36	24.94	0.49	3.57E-03	1.50E-02
Ube2b	107.18	52.07	0.49	7.13E-06	6.18E-05
Sat1	303.14	147.27	0.49	8.91E-07	9.16E-06
Mtmr10	91.71	44.56	0.49	4.70E-09	6.80E-08
H1f0	134.42	65.33	0.49	1.52E-08	2.05E-07
Taf13	40.99	19.94	0.49	3.71E-03	1.55E-02
Abcb4	81.33	39.65	0.49	6.06E-06	5.37E-05
Nsdhl	47.74	23.30	0.49	4.73E-04	2.65E-03
Mrpl41	23.76	11.66	0.49	1.27E-03	6.33E-03
Fam64a	65.66	32.25	0.49	4.21E-06	3.82E-05
Gins1	26.09	12.82	0.49	1.20E-03	6.03E-03

Klhl24	148.92	73.19	0.49	1.08E-07	1.28E-06
Cd48	215.55	106.00	0.49	5.03E-11	9.19E-10
Med4	47.11	23.18	0.49	1.81E-03	8.52E-03
Gtf2e2	48.55	23.89	0.49	4.98E-03	1.98E-02
Gng5	127.79	62.91	0.49	1.55E-06	1.53E-05
Hirip3	77.46	38.17	0.49	6.99E-05	4.89E-04
Lpxn	287.92	141.89	0.49	1.01E-12	2.23E-11
Msh2	52.56	25.94	0.49	1.20E-05	9.78E-05
Clk1	106.84	52.80	0.49	5.00E-05	3.58E-04
AF251705	153.80	76.03	0.49	5.33E-05	3.79E-04
Stra13	53.03	26.22	0.49	1.56E-04	9.96E-04
Rpl39	524.33	259.28	0.49	3.05E-09	4.50E-08
Pde3b	43.86	21.70	0.49	3.79E-04	2.19E-03
Rap2b	362.39	179.35	0.49	5.77E-12	1.15E-10
Fgd3	171.55	84.93	0.50	8.84E-09	1.23E-07
Pvrl3	14.16	7.02	0.50	1.70E-03	8.08E-03
Il10rb	325.04	161.22	0.50	4.91E-13	1.13E-11
Smox	56.99	28.33	0.50	2.12E-04	1.31E-03
Bcl2l11	106.39	52.91	0.50	1.52E-07	1.76E-06
Nav1	531.58	264.44	0.50	3.55E-12	7.31E-11
Nck1	45.73	22.76	0.50	1.11E-04	7.38E-04
Cst3	1065.32	530.23	0.50	1.98E-07	2.24E-06
Rpl22l1	36.65	18.27	0.50	2.83E-03	1.23E-02

Supplemental Table 3 – GO terms regulated by GH

GO Terms Biological Process	Stat Mean	P-Value	Set size
**Up-regulated**			
GO:0051707 response to other organism	6.74	2.62E-11	283
GO:0009607 response to biotic stimulus	6.68	3.52E-11	297
GO:0006952 defense response	6.42	1.32E-10	408
GO:0051704 multi-organism process	6.08	9.78E-10	433
GO:0045087 innate immune response	5.84	7.14E-09	199
GO:0006955 immune response	5.69	9.65E-09	400
GO:0009617 response to bacterium	5.17	2.44E-07	173
GO:0002252 immune effector process	4.85	8.68E-07	249
GO:0034097 response to cytokine stimulus	4.57	3.50E-06	214
GO:0009615 response to virus	4.53	0.00001	112
GO:0002221 pattern recognition receptor signaling pathway	4.66	0.00001	53
GO:0002237 response to molecule of bacterial origin	4.50	0.00001	122
GO:0051607 defense response to virus	4.52	0.00001	92
GO:0002218 activation of innate immune response	4.56	0.00001	62
GO:0002758 innate immune response-activating signal transduction	4.59	0.00001	54
GO:0045088 regulation of innate immune response	4.45	0.00001	98
GO:0022610 biological adhesion	4.33	0.00001	331
GO:0032496 response to lipopolysaccharide	4.43	0.00001	107
GO:0033993 response to lipid	4.32	0.00001	215
GO:0001816 cytokine production	4.28	0.00001	236
GO:0001817 regulation of cytokine production	4.25	0.00001	210
GO:0045089 positive regulation of innate immune response	4.33	0.00001	78
GO:0002224 toll-like receptor signaling pathway	4.39	0.00002	41
GO:0071345 cellular response to cytokine stimulus	3.99	0.00004	167

GO:0002682 regulation of immune system process	3.94	0.00004	417
GO:0031347 regulation of defense response	3.88	0.00006	185
GO:0007155 cell adhesion	3.81	0.00008	326
GO:0071219 cellular response to molecule of bacterial origin	3.92	0.00008	71
GO:0002253 activation of immune response	3.82	0.00009	125
GO:0035458 cellular response to interferon-beta	4.49	0.00009	18
GO:0009611 response to wounding	3.76	0.00010	312
GO:0080134 regulation of response to stress	3.69	0.00012	387
GO:0002757 immune response-activating signal transduction	3.71	0.00014	106
GO:0071216 cellular response to biotic stimulus	3.75	0.00014	82
GO:0002684 positive regulation of immune system process	3.64	0.00015	269
GO:0002764 immune response-regulating signaling pathway	3.67	0.00016	111
GO:0051092 positive regulation of NF-kappaB transcription factor activity	3.64	0.00022	57
GO:0031349 positive regulation of defense response	3.58	0.00022	107
GO:0035456 response to interferon-beta	3.99	0.00023	22
GO:0001819 positive regulation of cytokine production	3.56	0.00023	113
GO:0071396 cellular response to lipid	3.50	0.00031	111
GO:0071222 cellular response to lipopolysaccharide	3.56	0.00031	64
GO:0006954 inflammatory response	3.41	0.00037	205
GO:0001562 response to protozoan	3.93	0.00040	17
GO:1901700 response to oxygen-containing compound	3.36	0.00042	338
GO:0009306 protein secretion	3.40	0.00045	89
GO:0016477 cell migration	3.33	0.00045	380
GO:0034121 regulation of toll-like receptor signaling pathway	3.63	0.00054	20
GO:0042832 defense response to protozoan	3.87	0.00057	15
GO:0016337 cell-cell adhesion	3.30	0.00057	117
GO:0051090 regulation of sequence-specific DNA binding transcription factor activity	3.26	0.00061	183
GO:0051091 positive regulation of sequence-specific DNA binding transcription factor activity	3.25	0.00069	97

GO:0042742 defense response to bacterium	3.28	0.00078	62
GO:0030334 regulation of cell migration	3.18	0.00080	217
GO:0050776 regulation of immune response	3.16	0.00084	234
GO:0040011 locomotion	3.13	0.00091	465
GO:1901701 cellular response to oxygen-containing compound	3.09	0.00109	189
GO:0072358 cardiovascular system development	3.07	0.00113	356
GO:0072359 circulatory system development	3.07	0.00113	356
GO:0050778 positive regulation of immune response	3.04	0.00127	167
GO:0050663 cytokine secretion	3.11	0.00130	51
GO:0051223 regulation of protein transport	3.03	0.00131	177
GO:0048870 cell motility	3.01	0.00136	409
GO:0051674 localization of cell	3.01	0.00136	409
GO:0070201 regulation of establishment of protein localization	3.00	0.00145	197
GO:0009605 response to external stimulus	2.98	0.00150	416
GO:0032880 regulation of protein localization	2.97	0.00155	235
GO:0050708 regulation of protein secretion	3.03	0.00157	65
GO:0001568 blood vessel development	2.94	0.00175	225
GO:2000145 regulation of cell motility	2.93	0.00181	232
GO:0001944 vasculature development	2.90	0.00197	236
GO:0034341 response to interferon-gamma	3.07	0.00203	32
GO:0002697 regulation of immune effector process	2.88	0.00220	113
GO:0051240 positive regulation of multicellular organismal process	2.84	0.00237	236
GO:0060341 regulation of cellular localization	2.83	0.00243	345
GO:0051270 regulation of cellular component movement	2.83	0.00245	253
GO:0007159 leukocyte cell-cell adhesion	2.98	0.00255	23
GO:0050830 defense response to Gram-positive bacterium	2.98	0.00258	27
GO:0040012 regulation of locomotion	2.77	0.00292	240
GO:0046903 secretion	2.76	0.00298	309

GO:0050715 positive regulation of cytokine secretion	2.85	0.00313	36
GO:0032606 type I interferon production	2.81	0.00323	34
GO:0032481 positive regulation of type I interferon production	2.86	0.00332	22
GO:0030335 positive regulation of cell migration	2.73	0.00347	132
GO:0002753 cytoplasmic pattern recognition receptor signaling pathway	2.99	0.00348	13
GO:0032940 secretion by cell	2.70	0.00362	275
GO:0034142 toll-like receptor 4 signaling pathway	2.95	0.00391	12
GO:0007243 intracellular protein kinase cascade	2.66	0.00397	492
GO:0040017 positive regulation of locomotion	2.67	0.00411	136
GO:2000147 positive regulation of cell motility	2.67	0.00412	135
GO:0032270 positive regulation of cellular protein metabolic process	2.63	0.00429	401
GO:0050714 positive regulation of protein secretion	2.69	0.00442	45
GO:0071346 cellular response to interferon-gamma	2.84	0.00454	22
GO:0051272 positive regulation of cellular component movement	2.63	0.00462	141
GO:0043069 negative regulation of programmed cell death	2.61	0.00463	357
GO:0050707 regulation of cytokine secretion	2.68	0.00467	43
GO:0071902 positive regulation of protein serine/threonine kinase activity	2.62	0.00468	121
GO:0032479 regulation of type I interferon production	2.66	0.00506	29
GO:0007249 I-kappaB kinase/NF-kappaB cascade	2.56	0.00558	138
GO:0048771 tissue remodeling	2.59	0.00566	66
GO:0051247 positive regulation of protein metabolic process	2.54	0.00570	451
GO:0048585 negative regulation of response to stimulus	2.52	0.00600	413
GO:0031663 lipopolysaccharide-mediated signaling pathway	2.58	0.00628	32
GO:0035023 regulation of Rho protein signal transduction	2.50	0.00660	88
GO:0048514 blood vessel morphogenesis	2.49	0.00666	195
GO:0043066 negative regulation of apoptotic process	2.48	0.00666	352
GO:0043330 response to exogenous dsRNA	2.62	0.00684	20
GO:0032615 interleukin-12 production	2.57	0.00705	28

GO:0060548 negative regulation of cell death	2.46	0.00710	376
GO:0007507 heart development	2.46	0.00715	185
GO:0010647 positive regulation of cell communication	2.44	0.00739	450
GO:0043900 regulation of multi-organism process	2.46	0.00756	98
GO:0043901 negative regulation of multi-organism process	2.51	0.00775	35
GO:0031401 positive regulation of protein modification process	2.42	0.00795	342
GO:0032728 positive regulation of interferon-beta production	2.52	0.00826	19
GO:0032655 regulation of interleukin-12 production	2.51	0.00835	26
GO:0023056 positive regulation of signaling	2.39	0.00856	447
GO:0001775 cell activation	2.39	0.00864	352
GO:0051222 positive regulation of protein transport	2.39	0.00893	103
GO:0032648 regulation of interferon-beta production	2.45	0.00894	27
GO:0006935 chemotaxis	2.38	0.00894	144
GO:0045071 negative regulation of viral genome replication	2.53	0.00894	20
GO:0002718 regulation of cytokine production involved in immune response	2.48	0.00923	22
GO:0051046 regulation of secretion	2.36	0.00944	190
GO:0010876 lipid localization	2.36	0.00956	125
GO:0042116 macrophage activation	2.42	0.00973	29
GO:0048525 negative regulation of viral reproduction	2.48	0.00977	22
GO:0042330 taxis	2.35	0.00991	145
GO:0071900 regulation of protein serine/threonine kinase activity	2.33	0.01028	199
GO:0009967 positive regulation of signal transduction	2.32	0.01029	430
GO:0044403 symbiosis, encompassing mutualism through parasitism	2.33	0.01057	91
GO:0044419 interspecies interaction between organisms	2.33	0.01057	91
GO:0051336 regulation of hydrolase activity	2.31	0.01060	379
GO:0006897 endocytosis	2.30	0.01102	239
GO:0002699 positive regulation of immune effector process	2.31	0.01143	66
GO:0042060 wound healing	2.29	0.01143	109

GO:0046822 regulation of nucleocytoplasmic transport	2.29	0.01144	102
GO:0042991 transcription factor import into nucleus	2.31	0.01149	46
GO:0030155 regulation of cell adhesion	2.29	0.01154	138
GO:0032608 interferon-beta production	2.34	0.01156	29
GO:0001525 angiogenesis	2.28	0.01162	166
GO:0001934 positive regulation of protein phosphorylation	2.27	0.01171	258
GO:0043122 regulation of I-kappaB kinase/NF-kappaB cascade	2.28	0.01179	121
GO:0043551 regulation of phosphatidylinositol 3-kinase activity	2.39	0.01190	16
GO:0030163 protein catabolic process	2.26	0.01194	386
GO:0010562 positive regulation of phosphorus metabolic process	2.25	0.01227	298
GO:0045937 positive regulation of phosphate metabolic process	2.25	0.01227	298
GO:0002443 leukocyte mediated immunity	2.25	0.01267	114
GO:0045428 regulation of nitric oxide biosynthetic process	2.31	0.01293	26
GO:0051056 regulation of small GTPase mediated signal transduction	2.24	0.01295	201
GO:0042107 cytokine metabolic process	2.26	0.01301	52
GO:0032495 response to muramyl dipeptide	2.47	0.01322	10
GO:0043085 positive regulation of catalytic activity	2.22	0.01324	426
GO:0001932 regulation of protein phosphorylation	2.22	0.01327	418
GO:0009719 response to endogenous stimulus	2.22	0.01336	378
GO:0042990 regulation of transcription factor import into nucleus	2.25	0.01347	44
GO:0002683 negative regulation of immune system process	2.22	0.01376	101
GO:0045429 positive regulation of nitric oxide biosynthetic process	2.32	0.01396	19
GO:0045766 positive regulation of angiogenesis	2.25	0.01400	39
GO:0042327 positive regulation of phosphorylation	2.20	0.01420	269
GO:0006809 nitric oxide biosynthetic process	2.28	0.01425	31
GO:0032088 negative regulation of NF-kappaB transcription factor activity	2.22	0.01448	48
GO:0050865 regulation of cell activation	2.19	0.01456	187
GO:0046209 nitric oxide metabolic process	2.26	0.01460	34

GO:0002456 T cell mediated immunity	2.24	0.01470	33
GO:0002698 negative regulation of immune effector process	2.22	0.01496	40
GO:0070482 response to oxygen levels	2.20	0.01502	76
GO:0043331 response to dsRNA	2.23	0.01506	31
GO:0002367 cytokine production involved in immune response	2.24	0.01539	27
GO:0002274 myeloid leukocyte activation	2.17	0.01580	80
GO:0032101 regulation of response to external stimulus	2.16	0.01595	188
GO:0045765 regulation of angiogenesis	2.17	0.01606	70
GO:0030198 extracellular matrix organization	2.17	0.01628	51
GO:0043062 extracellular structure organization	2.17	0.01628	51
GO:0016045 detection of bacterium	2.28	0.01659	12
GO:0006865 amino acid transport	2.15	0.01667	59
GO:0002460 adaptive immune response based on somatic recombination of immune receptors built from immunoglobulin superfamily domains	2.14	0.01676	103
GO:0060840 artery development	2.20	0.01697	20
GO:0007169 transmembrane receptor protein tyrosine kinase signaling pathway	2.13	0.01699	170
GO:0002831 regulation of response to biotic stimulus	2.17	0.01699	40
GO:0002700 regulation of production of molecular mediator of immune response	2.18	0.01704	35
GO:0002263 cell activation involved in immune response	2.13	0.01739	98
GO:0002366 leukocyte activation involved in immune response	2.13	0.01739	98
GO:0022603 regulation of anatomical structure morphogenesis	2.12	0.01741	290
GO:0010648 negative regulation of cell communication	2.11	0.01757	347
GO:0002250 adaptive immune response	2.12	0.01771	111
GO:0031348 negative regulation of defense response	2.13	0.01788	57
GO:0023057 negative regulation of signaling	2.10	0.01812	344
GO:0000041 transition metal ion transport	2.12	0.01832	46
GO:0051050 positive regulation of transport	2.09	0.01856	268
GO:0010627 regulation of intracellular protein kinase cascade	2.09	0.01857	389

GO:0043405 regulation of MAP kinase activity	2.09	0.01863	129
GO:0019221 cytokine-mediated signaling pathway	2.10	0.01864	107
GO:0018108 peptidyl-tyrosine phosphorylation	2.09	0.01888	102
GO:0002703 regulation of leukocyte mediated immunity	2.10	0.01898	66
GO:0022008 neurogenesis	2.07	0.01927	456
GO:0030182 neuron differentiation	2.07	0.01946	371
GO:0018212 peptidyl-tyrosine modification	2.07	0.01986	104
GO:0051047 positive regulation of secretion	2.07	0.01990	105
GO:0009791 post-embryonic development	2.07	0.02006	62
GO:0002449 lymphocyte mediated immunity	2.07	0.02017	89
GO:0002281 macrophage activation involved in immune response	2.18	0.02025	13
GO:0051169 nuclear transport	2.05	0.02039	222
GO:0006913 nucleocytoplasmic transport	2.04	0.02118	219
GO:0045321 leukocyte activation	2.03	0.02126	320
GO:0042325 regulation of phosphorylation	2.03	0.02130	461
GO:0009894 regulation of catabolic process	2.03	0.02135	341
GO:0048661 positive regulation of smooth muscle cell proliferation	2.07	0.02143	33
GO:0002694 regulation of leukocyte activation	2.03	0.02155	175
GO:0007219 Notch signaling pathway	2.05	0.02167	53
GO:1900180 regulation of protein localization to nucleus	2.03	0.02181	82
GO:0007259 JAK-STAT cascade	2.05	0.02232	40
GO:0045860 positive regulation of protein kinase activity	2.00	0.02326	174
GO:0090066 regulation of anatomical structure size	1.99	0.02357	166
GO:0034340 response to type I interferon	2.12	0.02371	14
GO:0032319 regulation of Rho GTPase activity	2.00	0.02417	46
GO:0038061 NIK/NF-kappaB cascade	2.15	0.02421	11
GO:0042089 cytokine biosynthetic process	2.00	0.02424	51
GO:0032635 interleukin-6 production	2.00	0.02437	47

GO:0032675 regulation of interleukin-6 production	2.00	0.02437	47
GO:0051345 positive regulation of hydrolase activity	1.97	0.02464	195
GO:0048878 chemical homeostasis	1.95	0.02582	375
GO:0060042 retina morphogenesis in camera-type eye	2.07	0.02603	11
GO:0060326 cell chemotaxis	1.96	0.02606	75
GO:0046578 regulation of Ras protein signal transduction	1.95	0.02617	186
GO:0034122 negative regulation of toll-like receptor signaling pathway	2.08	0.02626	11
GO:0007167 enzyme linked receptor protein signaling pathway	1.94	0.02633	268
GO:0042345 regulation of NF-kappaB import into nucleus	2.01	0.02642	20
GO:0042348 NF-kappaB import into nucleus	2.01	0.02642	20
GO:0030595 leukocyte chemotaxis	1.96	0.02655	61
GO:0072593 reactive oxygen species metabolic process	1.96	0.02664	75
GO:0052547 regulation of peptidase activity	1.94	0.02677	149
GO:0007186 G-protein coupled receptor signaling pathway	1.94	0.02699	181
GO:0022415 viral reproductive process	1.94	0.02723	102
GO:0007044 cell-substrate junction assembly	1.97	0.02741	30
GO:0055006 cardiac cell development	1.97	0.02793	20
GO:0060485 mesenchyme development	1.93	0.02795	51
GO:1901342 regulation of vasculature development	1.93	0.02822	73
GO:0032611 interleukin-1 beta production	1.97	0.02824	21
GO:0050730 regulation of peptidyl-tyrosine phosphorylation	1.92	0.02854	74
GO:0042306 regulation of protein import into nucleus	1.92	0.02858	80
GO:0048659 smooth muscle cell proliferation	1.92	0.02896	53
GO:0045069 regulation of viral genome replication	1.94	0.02950	32
GO:0048660 regulation of smooth muscle cell proliferation	1.91	0.02959	52
GO:0016032 viral reproduction	1.90	0.02968	118
GO:0034333 adherens junction assembly	1.94	0.02997	25
GO:0048844 artery morphogenesis	1.95	0.02998	17

GO:0010810 regulation of cell-substrate adhesion	1.90	0.03044	54
GO:0006909 phagocytosis	1.89	0.03050	70
GO:0001952 regulation of cell-matrix adhesion	1.91	0.03107	34
GO:0034332 adherens junction organization	1.91	0.03118	28
GO:0045859 regulation of protein kinase activity	1.86	0.03134	299
GO:0032386 regulation of intracellular transport	1.87	0.03136	158
GO:0044703 multi-organism reproductive process	1.87	0.03137	151
GO:0042491 auditory receptor cell differentiation	1.97	0.03161	12
GO:0044764 multi-organism cellular process	1.86	0.03184	119
GO:0033002 muscle cell proliferation	1.87	0.03195	67
GO:0033674 positive regulation of kinase activity	1.86	0.03197	187
GO:0061138 morphogenesis of a branching epithelium	1.87	0.03200	74
GO:0051702 interaction with symbiont	1.92	0.03207	26
GO:0002286 T cell activation involved in immune response	1.89	0.03226	28
GO:0018193 peptidyl-amino acid modification	1.85	0.03243	398
GO:0042347 negative regulation of NF-kappaB import into nucleus	1.97	0.03245	10
GO:0043552 positive regulation of phosphatidylinositol 3-kinase activity	1.95	0.03285	12
GO:2000377 regulation of reactive oxygen species metabolic process	1.87	0.03296	39
GO:0051051 negative regulation of transport	1.84	0.03307	145
GO:0015711 organic anion transport	1.84	0.03373	109
GO:0009790 embryo development	1.83	0.03385	458
GO:0032321 positive regulation of Rho GTPase activity	1.86	0.03388	35
GO:0042993 positive regulation of transcription factor import into nucleus	1.90	0.03411	17
GO:0001666 response to hypoxia	1.84	0.03415	74
GO:0036293 response to decreased oxygen levels	1.84	0.03415	74
GO:0002720 positive regulation of cytokine production involved in immune response	1.95	0.03421	13
GO:0003007 heart morphogenesis	1.83	0.03491	66
GO:0043433 negative regulation of sequence-specific DNA binding transcription factor activity	1.83	0.03493	82

GO:0015807 L-amino acid transport	1.86	0.03510	25
GO:0050900 leukocyte migration	1.83	0.03511	83
GO:0043550 regulation of lipid kinase activity	1.86	0.03516	21
GO:0046847 filopodium assembly	1.84	0.03532	31
GO:0009968 negative regulation of signal transduction	1.81	0.03550	333
GO:0015849 organic acid transport	1.81	0.03567	97
GO:0046942 carboxylic acid transport	1.81	0.03567	97
GO:0007160 cell-matrix adhesion	1.82	0.03568	61
GO:0050867 positive regulation of cell activation	1.81	0.03569	120
GO:0043406 positive regulation of MAP kinase activity	1.81	0.03583	90
GO:0033619 membrane protein proteolysis	1.84	0.03583	28
GO:0009593 detection of chemical stimulus	1.88	0.03621	16
GO:0048699 generation of neurons	1.80	0.03645	418
GO:0007162 negative regulation of cell adhesion	1.82	0.03662	47
GO:0002695 negative regulation of leukocyte activation	1.81	0.03697	56
GO:0051341 regulation of oxidoreductase activity	1.82	0.03725	29
GO:0042307 positive regulation of protein import into nucleus	1.80	0.03861	32
GO:0050792 regulation of viral reproduction	1.79	0.03865	54
GO:0051250 negative regulation of lymphocyte activation	1.78	0.03943	46
GO:0055080 cation homeostasis	1.76	0.03968	184
GO:0001953 negative regulation of cell-matrix adhesion	1.89	0.03991	12
GO:0048666 neuron development	1.75	0.04010	292
GO:0006606 protein import into nucleus	1.75	0.04026	137
GO:0044744 protein targeting to nucleus	1.75	0.04026	137
GO:0034612 response to tumor necrosis factor	1.78	0.04068	41
GO:0002709 regulation of T cell mediated immunity	1.79	0.04095	25
GO:0048041 focal adhesion assembly	1.80	0.04143	21
GO:0034330 cell junction organization	1.74	0.04186	67

GO:0031329 regulation of cellular catabolic process	1.73	0.04190	295
GO:1901698 response to nitrogen compound	1.73	0.04250	222
GO:0006869 lipid transport	1.73	0.04250	104
GO:0016567 protein ubiquitination	1.72	0.04262	311
GO:0007250 activation of NF-kappaB-inducing kinase activity	1.86	0.04272	10
GO:1901222 regulation of NIK/NF-kappaB cascade	1.86	0.04272	10
GO:0007435 salivary gland morphogenesis	1.80	0.04293	13
GO:0035137 hindlimb morphogenesis	1.81	0.04297	15
GO:0051170 nuclear import	1.72	0.04347	138
GO:0042992 negative regulation of transcription factor import into nucleus	1.75	0.04365	25
GO:0034329 cell junction assembly	1.73	0.04375	56
GO:0035272 exocrine system development	1.76	0.04421	17
GO:0010812 negative regulation of cell-substrate adhesion	1.78	0.04421	17
GO:0051701 interaction with host	1.72	0.04426	62
GO:0006801 superoxide metabolic process	1.77	0.04435	24
GO:0008360 regulation of cell shape	1.72	0.04440	60
GO:0045995 regulation of embryonic development	1.73	0.04473	29
GO:0019941 modification-dependent protein catabolic process	1.70	0.04475	283
GO:0001892 embryonic placenta development	1.72	0.04479	46
GO:0034394 protein localization to cell surface	1.77	0.04483	13
GO:0051347 positive regulation of transferase activity	1.70	0.04491	194
GO:0001974 blood vessel remodeling	1.78	0.04513	19
GO:0043123 positive regulation of I-kappaB kinase/NF-kappaB cascade	1.70	0.04516	84
GO:0046777 protein autophosphorylation	1.70	0.04526	127
GO:0001774 microglial cell activation	1.79	0.04528	11
GO:0003018 vascular process in circulatory system	1.72	0.04540	52
GO:0022407 regulation of cell-cell adhesion	1.71	0.04547	39
GO:0035710 CD4-positive, alpha-beta T cell activation	1.73	0.04556	25

GO:0043367 CD4-positive, alpha-beta T cell differentiation	1.73	0.04556	25
GO:0050878 regulation of body fluid levels	1.69	0.04597	95
GO:0009725 response to hormone stimulus	1.69	0.04618	207
GO:0008285 negative regulation of cell proliferation	1.68	0.04672	238
GO:0010740 positive regulation of intracellular protein kinase cascade	1.68	0.04683	246
GO:0008593 regulation of Notch signaling pathway	1.73	0.04691	19
GO:0051893 regulation of focal adhesion assembly	1.78	0.04691	13
GO:0090109 regulation of cell-substrate junction assembly	1.78	0.04691	13
GO:0002706 regulation of lymphocyte mediated immunity	1.69	0.04744	49
GO:0043632 modification-dependent macromolecule catabolic process	1.67	0.04763	286
GO:0019079 viral genome replication	1.70	0.04805	35
GO:0030522 intracellular receptor mediated signaling pathway	1.67	0.04825	97
GO:0050702 interleukin-1 beta secretion	1.73	0.04827	13
GO:0003333 amino acid transmembrane transport	1.71	0.04831	21
GO:0006986 response to unfolded protein	1.68	0.04832	53
GO:0030029 actin filament-based process	1.66	0.04839	241
GO:2000117 negative regulation of cysteine-type endopeptidase activity	1.67	0.04870	50
GO:0046632 alpha-beta T cell differentiation	1.68	0.04874	38
GO:0070613 regulation of protein processing	1.72	0.04898	17
GO:0009057 macromolecule catabolic process	1.66	0.04900	495
GO:0042310 vasoconstriction	1.69	0.04922	25
GO:0051224 negative regulation of protein transport	1.67	0.04922	55
GO:0045824 negative regulation of innate immune response	1.74	0.04931	11
GO:0002275 myeloid cell activation involved in immune response	1.67	0.04951	40
GO:0006509 membrane protein ectodomain proteolysis	1.68	0.04954	24
GO:0019229 regulation of vasoconstriction	1.71	0.04982	18
**Down-regulated**			

GO:0000279 M phase	-4.58	2.76E-06	349
GO:0022403 cell cycle phase	-4.57	2.84E-06	470
GO:0051301 cell division	-4.11	0.00002	359
GO:0006260 DNA replication	-4.08	0.00003	193
GO:0006281 DNA repair	-4.06	0.00003	305
GO:0000280 nuclear division	-3.77	0.00009	260
GO:0007067 mitosis	-3.77	0.00009	260
GO:0000087 M phase of mitotic cell cycle	-3.74	0.00010	266
GO:0048285 organelle fission	-3.67	0.00014	276
GO:0000278 mitotic cell cycle	-3.56	0.00019	438
GO:0006261 DNA-dependent DNA replication	-3.53	0.00029	65
GO:0007126 meiosis	-3.40	0.00043	82
GO:0051327 M phase of meiotic cell cycle	-3.40	0.00043	82
GO:0051321 meiotic cell cycle	-3.27	0.00065	87
GO:0007127 meiosis I	-3.16	0.00111	43
GO:0007059 chromosome segregation	-2.97	0.00162	123
GO:0010564 regulation of cell cycle process	-2.87	0.00213	254
GO:0006284 base-excision repair	-3.02	0.00221	23
GO:0006974 response to DNA damage stimulus	-2.80	0.00258	434
GO:0006396 RNA processing	-2.80	0.00259	467
GO:0007131 reciprocal meiotic recombination	-2.96	0.00263	20
GO:0035825 reciprocal DNA recombination	-2.96	0.00263	20
GO:0006302 double-strand break repair	-2.69	0.00390	94
GO:0008380 RNA splicing	-2.65	0.00418	220
GO:0071156 regulation of cell cycle arrest	-2.64	0.00442	134
GO:0070925 organelle assembly	-2.60	0.00506	90
GO:0015992 proton transport	-2.62	0.00518	48
GO:0006298 mismatch repair	-2.75	0.00586	12

GO:0007346 regulation of mitotic cell cycle	-2.50	0.00652	177
GO:0000075 cell cycle checkpoint	-2.46	0.00731	118
GO:0031570 DNA integrity checkpoint	-2.44	0.00793	66
GO:0045005 maintenance of fidelity involved in DNA-dependent DNA replication	-2.64	0.00864	12
GO:0006310 DNA recombination	-2.36	0.00939	140
GO:0051276 chromosome organization	-2.35	0.00946	500
GO:0000725 recombinational repair	-2.40	0.00946	44
GO:0007051 spindle organization	-2.30	0.01161	64
GO:0006818 hydrogen transport	-2.30	0.01188	49
GO:0071103 DNA conformation change	-2.26	0.01251	99
GO:0042451 purine nucleoside biosynthetic process	-2.24	0.01351	60
GO:0046129 purine ribonucleoside biosynthetic process	-2.24	0.01351	60
GO:0000724 double-strand break repair via homologous recombination	-2.25	0.01368	43
GO:0006397 mRNA processing	-2.19	0.01436	275
GO:0006270 DNA replication initiation	-2.31	0.01525	16
GO:0000077 DNA damage checkpoint	-2.17	0.01617	62
GO:0022900 electron transport chain	-2.13	0.01730	86
GO:0051297 centrosome organization	-2.13	0.01755	55
GO:0000086 G2/M transition of mitotic cell cycle	-2.10	0.01949	41
GO:0006412 translation	-2.07	0.01953	343
GO:0042274 ribosomal small subunit biogenesis	-2.15	0.01993	20
GO:0033108 mitochondrial respiratory chain complex assembly	-2.13	0.02056	19
GO:0009163 nucleoside biosynthetic process	-2.06	0.02063	68
GO:0042455 ribonucleoside biosynthetic process	-2.06	0.02063	68
GO:1901659 glycosyl compound biosynthetic process	-2.06	0.02063	68
GO:0051225 spindle assembly	-2.08	0.02091	35
GO:0016071 mRNA metabolic process	-2.03	0.02167	318
GO:0006323 DNA packaging	-2.02	0.02252	69

GO:0010948 negative regulation of cell cycle process	-2.00	0.02381	62
GO:0015985 energy coupled proton transport, down electrochemical gradient	-2.09	0.02422	14
GO:0015986 ATP synthesis coupled proton transport	-2.09	0.02422	14
GO:0031023 microtubule organizing center organization	-1.95	0.02656	59
GO:0034440 lipid oxidation	-1.94	0.02789	51
GO:0051726 regulation of cell cycle	-1.92	0.02790	434
GO:0035590 purinergic nucleotide receptor signaling pathway	-2.03	0.02799	11
GO:0019395 fatty acid oxidation	-1.91	0.02955	50
GO:0008406 gonad development	-1.90	0.02984	62
GO:0045132 meiotic chromosome segregation	-1.95	0.03100	15
GO:0070192 chromosome organization involved in meiosis	-1.94	0.03204	14
GO:0007129 synapsis	-1.96	0.03271	11
GO:0000288 nuclear-transcribed mRNA catabolic process, deadenylation-dependent decay	-1.91	0.03340	16
GO:0006091 generation of precursor metabolites and energy	-1.82	0.03446	202
GO:0042773 ATP synthesis coupled electron transport	-1.86	0.03667	15
GO:0009142 nucleoside triphosphate biosynthetic process	-1.81	0.03699	39
GO:0009145 purine nucleoside triphosphate biosynthetic process	-1.81	0.03737	35
GO:0006119 oxidative phosphorylation	-1.81	0.03848	23
GO:0007050 cell cycle arrest	-1.77	0.03911	171
GO:0007224 smoothened signaling pathway	-1.78	0.03982	40
GO:0051325 interphase	-1.76	0.03987	161
GO:0006364 rRNA processing	-1.69	0.04618	98
GO:0031576 G2/M transition checkpoint	-1.70	0.04719	35
GO:2000602 regulation of interphase of mitotic cell cycle	-1.68	0.04751	80
GO:0007143 female meiosis	-1.74	0.04943	12
GO:0045786 negative regulation of cell cycle	-1.65	0.04974	217

Supplemental Table 4 – GO terms regulated by IFN

Go terms Biological process	Stat Mean	P-value	Set size
**Up-regulated**			
GO:0006952 defense response	7.57	8.08E-14	408
GO:0045087 innate immune response	7.39	1.05E-12	199
GO:0006955 immune response	7.09	2.02E-12	400
GO:0051707 response to other organism	7.01	5.52E-12	283
GO:0009607 response to biotic stimulus	6.92	9.21E-12	297
GO:0051704 multi-organism process	6.56	5.77E-11	433
GO:0002252 immune effector process	6.48	1.42E-10	249
GO:0002682 regulation of immune system process	5.64	1.26E-08	417
GO:0045088 regulation of innate immune response	5.53	6.41E-08	98
GO:0034097 response to cytokine stimulus	5.40	7.16E-08	214
GO:0051607 defense response to virus	5.54	8.30E-08	92
GO:0002684 positive regulation of immune system process	5.15	2.04E-07	269
GO:0009615 response to virus	5.27	2.19E-07	112
GO:0009617 response to bacterium	5.11	3.36E-07	173
GO:0031347 regulation of defense response	5.07	3.57E-07	185
GO:0050776 regulation of immune response	4.99	4.63E-07	234
GO:0071345 cellular response to cytokine stimulus	5.04	4.97E-07	167
GO:0019882 antigen processing and presentation	5.19	9.70E-07	42
GO:0050778 positive regulation of immune response	4.66	2.49E-06	167
GO:0080134 regulation of response to stress	4.54	3.34E-06	387
GO:0022610 biological adhesion	4.51	3.87E-06	331
GO:0002218 activation of innate immune response	4.68	4.39E-06	62
GO:0001816 cytokine production	4.48	4.86E-06	236
GO:0002460 adaptive immune response based on somatic recombination of immune receptors built from immunoglobulin superfamily domains	4.54	5.60E-06	103

GO:0002250 adaptive immune response	4.52	5.89E-06	111
GO:0035458 cellular response to interferon-beta	5.67	8.09E-06	18
GO:0002253 activation of immune response	4.37	9.82E-06	125
GO:0001817 regulation of cytokine production	4.32	1.03E-05	210
GO:0045089 positive regulation of innate immune response	4.36	1.29E-05	78
GO:0035456 response to interferon-beta	5.03	1.92E-05	22
GO:0032496 response to lipopolysaccharide	4.21	2.29E-05	107
GO:0002221 pattern recognition receptor signaling pathway	4.25	2.62E-05	53
GO:0002237 response to molecule of bacterial origin	4.13	2.94E-05	122
GO:0002697 regulation of immune effector process	4.11	3.03E-05	113
GO:0001775 cell activation	3.98	3.88E-05	352
GO:0002758 innate immune response-activating signal transduction	4.12	4.22E-05	54
GO:0009611 response to wounding	3.93	4.82E-05	312
GO:0007155 cell adhesion	3.92	4.92E-05	326
GO:0031349 positive regulation of defense response	3.91	0.0001	107
GO:0002764 immune response-regulating signaling pathway	3.86	0.0001	111
GO:0001819 positive regulation of cytokine production	3.86	0.0001	113
GO:0042832 defense response to protozoan	4.91	0.0001	15
GO:0023056 positive regulation of signaling	3.77	0.0001	447
GO:0002224 toll-like receptor signaling pathway	3.98	0.0001	41
GO:0010647 positive regulation of cell communication	3.76	0.0001	450
GO:0033993 response to lipid	3.77	0.0001	215
GO:0002449 lymphocyte mediated immunity	3.79	0.0001	89
GO:0001562 response to protozoan	4.60	0.0001	17
GO:1901700 response to oxygen-containing compound	3.64	0.0001	338
GO:0042742 defense response to bacterium	3.79	0.0002	62
GO:0034341 response to interferon-gamma	3.94	0.0002	32
GO:0045321 leukocyte activation	3.56	0.0002	320

GO:0009967 positive regulation of signal transduction	3.55	0.0002	430
GO:0051240 positive regulation of multicellular organismal process	3.52	0.0002	236
GO:0051090 regulation of sequence-specific DNA binding transcription factor activity	3.52	0.0002	183
GO:0006954 inflammatory response	3.51	0.0003	205
GO:0048002 antigen processing and presentation of peptide antigen	3.70	0.0003	29
GO:0051091 positive regulation of sequence-specific DNA binding transcription factor activity	3.53	0.0003	97
GO:0071346 cellular response to interferon-gamma	3.97	0.0003	22
GO:0002683 negative regulation of immune system process	3.45	0.0004	101
GO:0002757 immune response-activating signal transduction	3.41	0.0004	106
GO:0007243 intracellular protein kinase cascade	3.35	0.0004	492
GO:0002443 leukocyte mediated immunity	3.38	0.0004	114
GO:0002819 regulation of adaptive immune response	3.41	0.0005	67
GO:0071219 cellular response to molecule of bacterial origin	3.41	0.0005	71
GO:0002821 positive regulation of adaptive immune response	3.47	0.0005	39
GO:0071216 cellular response to biotic stimulus	3.32	0.0006	82
GO:0050867 positive regulation of cell activation	3.23	0.0007	120
GO:0046649 lymphocyte activation	3.19	0.0008	265
GO:0051092 positive regulation of NF-kappaB transcription factor activity	3.26	0.0008	57
GO:0043900 regulation of multi-organism process	3.20	0.0009	98
GO:0002699 positive regulation of immune effector process	3.19	0.0010	66
GO:0002824 positive regulation of adaptive immune response based on somatic recombination of immune receptors built from immunoglobulin superfamily domains	3.28	0.0010	34
GO:0019221 cytokine-mediated signaling pathway	3.14	0.0010	107
GO:0048534 hematopoietic or lymphoid organ development	3.09	0.0010	342
GO:0006909 phagocytosis	3.12	0.0011	70
GO:0071222 cellular response to lipopolysaccharide	3.16	0.0011	64
GO:0019884 antigen processing and presentation of exogenous antigen	3.28	0.0012	20
GO:0002696 positive regulation of leukocyte activation	3.06	0.0013	115

GO:0007249 I-kappaB kinase/NF-kappaB cascade	3.05	0.0013	138
GO:0050865 regulation of cell activation	3.03	0.0013	187
GO:0002520 immune system development	3.01	0.0013	367
GO:0001909 leukocyte mediated cytotoxicity	3.11	0.0014	34
GO:0051251 positive regulation of lymphocyte activation	2.97	0.0017	107
GO:0002822 regulation of adaptive immune response based on somatic recombination of immune receptors built from immunoglobulin superfamily domains	3.02	0.0017	58
GO:0034121 regulation of toll-like receptor signaling pathway	3.20	0.0017	20
GO:0002831 regulation of response to biotic stimulus	3.01	0.0018	40
GO:0046632 alpha-beta T cell differentiation	3.01	0.0019	38
GO:0035710 CD4-positive, alpha-beta T cell activation	3.09	0.0019	25
GO:0043367 CD4-positive, alpha-beta T cell differentiation	3.09	0.0019	25
GO:0045824 negative regulation of innate immune response	3.40	0.0019	11
GO:0030097 hemopoiesis	2.90	0.0019	328
GO:0051249 regulation of lymphocyte activation	2.92	0.0019	148
GO:0016337 cell-cell adhesion	2.90	0.0020	117
GO:0002286 T cell activation involved in immune response	3.02	0.0021	28
GO:0044403 symbiosis, encompassing mutualism through parasitism	2.93	0.0021	91
GO:0044419 interspecies interaction between organisms	2.93	0.0021	91
GO:0042110 T cell activation	2.88	0.0021	174
GO:0002705 positive regulation of leukocyte mediated immunity	3.02	0.0021	30
GO:0002708 positive regulation of lymphocyte mediated immunity	3.02	0.0021	30
GO:0048585 negative regulation of response to stimulus	2.86	0.0022	413
GO:0046903 secretion	2.86	0.0022	309
GO:0009605 response to external stimulus	2.86	0.0022	416
GO:0032940 secretion by cell	2.84	0.0024	275
GO:0002694 regulation of leukocyte activation	2.84	0.0025	175
GO:0010740 positive regulation of intracellular protein kinase cascade	2.81	0.0026	246

GO:0002456 T cell mediated immunity	2.93	0.0026	33
GO:0071396 cellular response to lipid	2.84	0.0026	111
GO:0045621 positive regulation of lymphocyte differentiation	2.87	0.0028	39
GO:0032655 regulation of interleukin-12 production	2.96	0.0028	26
GO:1901701 cellular response to oxygen-containing compound	2.78	0.0029	189
GO:0002478 antigen processing and presentation of exogenous peptide antigen	3.01	0.0029	16
GO:0050688 regulation of defense response to virus	2.90	0.0029	24
GO:0040011 locomotion	2.76	0.0030	465
GO:0070482 response to oxygen levels	2.79	0.0031	76
GO:0032615 interleukin-12 production	2.88	0.0032	28
GO:0031348 negative regulation of defense response	2.77	0.0034	57
GO:0043122 regulation of I-kappaB kinase/NF-kappaB cascade	2.72	0.0035	121
GO:0009306 protein secretion	2.73	0.0036	89
GO:0034340 response to type I interferon	3.02	0.0036	14
GO:0002753 cytoplasmic pattern recognition receptor signaling pathway	2.93	0.0037	13
GO:0044703 multi-organism reproductive process	2.69	0.0038	151
GO:0071902 positive regulation of protein serine/threonine kinase activity	2.70	0.0038	121
GO:0010627 regulation of intracellular protein kinase cascade	2.66	0.0040	389
GO:0051094 positive regulation of developmental process	2.65	0.0041	327
GO:0031401 positive regulation of protein modification process	2.64	0.0043	342
GO:0019724 B cell mediated immunity	2.70	0.0043	50
GO:0032606 type I interferon production	2.72	0.0044	34
GO:0002455 humoral immune response mediated by circulating immunoglobulin	2.90	0.0044	14
GO:0016477 cell migration	2.62	0.0044	380
GO:0051770 positive regulation of nitric-oxide synthase biosynthetic process	2.93	0.0045	11
GO:0045580 regulation of T cell differentiation	2.68	0.0045	44
GO:0001906 cell killing	2.72	0.0046	37
GO:0050691 regulation of defense response to virus by host	2.82	0.0046	15

GO:0032640	tumor necrosis factor production	2.66	0.0046	52
GO:0032680	regulation of tumor necrosis factor production	2.66	0.0046	52
GO:0022603	regulation of anatomical structure morphogenesis	2.61	0.0047	290
GO:0043901	negative regulation of multi-organism process	2.71	0.0047	35
GO:0002521	leukocyte differentiation	2.61	0.0047	211
GO:0048812	neuron projection morphogenesis	2.58	0.0051	169
GO:0002287	alpha-beta T cell activation involved in immune response	2.73	0.0052	19
GO:0007259	JAK-STAT cascade	2.65	0.0053	40
GO:0031663	lipopolysaccharide-mediated signaling pathway	2.64	0.0053	32
GO:0048667	cell morphogenesis involved in neuron differentiation	2.57	0.0053	163
GO:0050830	defense response to Gram-positive bacterium	2.72	0.0054	27
GO:0000904	cell morphogenesis involved in differentiation	2.56	0.0055	217
GO:0045582	positive regulation of T cell differentiation	2.64	0.0055	31
GO:0042327	positive regulation of phosphorylation	2.54	0.0057	269
GO:0001666	response to hypoxia	2.57	0.0058	74
GO:0036293	response to decreased oxygen levels	2.57	0.0058	74
GO:0030182	neuron differentiation	2.53	0.0058	371
GO:0002292	T cell differentiation involved in immune response	2.69	0.0059	18
GO:0002293	alpha-beta T cell differentiation involved in immune response	2.69	0.0059	18
GO:0002294	CD4-positive, alpha-beta T cell differentiation involved in immune response	2.69	0.0059	18
GO:0042093	T-helper cell differentiation	2.69	0.0059	18
GO:0051272	positive regulation of cellular component movement	2.53	0.0060	141
GO:0043330	response to exogenous dsRNA	2.66	0.0061	20
GO:0070304	positive regulation of stress-activated protein kinase signaling cascade	2.58	0.0061	34
GO:0071706	tumor necrosis factor superfamily cytokine production	2.56	0.0061	53
GO:0010648	negative regulation of cell communication	2.51	0.0061	347
GO:2000147	positive regulation of cell motility	2.53	0.0061	135
GO:0046637	regulation of alpha-beta T cell differentiation	2.61	0.0063	25

GO:0002698 negative regulation of immune effector process	2.57	0.0063	40
GO:0051270 regulation of cellular component movement	2.50	0.0063	253
GO:0016064 immunoglobulin mediated immune response	2.56	0.0063	49
GO:0023057 negative regulation of signaling	2.50	0.0064	344
GO:0030335 positive regulation of cell migration	2.51	0.0064	132
GO:0048870 cell motility	2.49	0.0065	409
GO:0051674 localization of cell	2.49	0.0065	409
GO:0002706 regulation of lymphocyte mediated immunity	2.55	0.0065	49
GO:0032481 positive regulation of type I interferon production	2.61	0.0068	22
GO:0051241 negative regulation of multicellular organismal process	2.48	0.0068	158
GO:0030334 regulation of cell migration	2.48	0.0068	217
GO:0045619 regulation of lymphocyte differentiation	2.50	0.0071	59
GO:0032101 regulation of response to external stimulus	2.47	0.0071	188
GO:0042092 type 2 immune response	2.68	0.0072	16
GO:0042060 wound healing	2.46	0.0074	109
GO:0022008 neurogenesis	2.44	0.0075	456
GO:0043123 positive regulation of I-kappaB kinase/NF-kappaB cascade	2.46	0.0076	84
GO:0046631 alpha-beta T cell activation	2.48	0.0077	51
GO:0040017 positive regulation of locomotion	2.44	0.0078	136
GO:0045597 positive regulation of cell differentiation	2.42	0.0080	249
GO:0032874 positive regulation of stress-activated MAPK cascade	2.47	0.0081	33
GO:0070302 regulation of stress-activated protein kinase signaling cascade	2.41	0.0084	94
GO:0001934 positive regulation of protein phosphorylation	2.39	0.0085	258
GO:0060759 regulation of response to cytokine stimulus	2.45	0.0085	37
GO:0006959 humoral immune response	2.47	0.0086	31
GO:0048525 negative regulation of viral reproduction	2.53	0.0086	22
GO:0032608 interferon-beta production	2.46	0.0088	29
GO:0046638 positive regulation of alpha-beta T cell differentiation	2.50	0.0088	20

GO:0072358 cardiovascular system development	2.38	0.0088	356
GO:0072359 circulatory system development	2.38	0.0088	356
GO:0001913 T cell mediated cytotoxicity	2.51	0.0088	18
GO:0030217 T cell differentiation	2.40	0.0089	98
GO:0050878 regulation of body fluid levels	2.39	0.0089	95
GO:0060429 epithelium development	2.37	0.0090	238
GO:0002703 regulation of leukocyte mediated immunity	2.40	0.0091	66
GO:0006897 endocytosis	2.37	0.0092	239
GO:0042088 T-helper 1 type immune response	2.51	0.0093	18
GO:0060341 regulation of cellular localization	2.36	0.0094	345
GO:2001236 regulation of extrinsic apoptotic signaling pathway	2.43	0.0095	25
GO:0050764 regulation of phagocytosis	2.41	0.0095	35
GO:0002504 antigen processing and presentation of peptide or polysaccharide antigen via MHC class II	2.56	0.0097	13
GO:2000145 regulation of cell motility	2.34	0.0098	232
GO:0007409 axonogenesis	2.34	0.0100	131
GO:0032872 regulation of stress-activated MAPK cascade	2.35	0.0100	93
GO:0070555 response to interleukin-1	2.40	0.0100	29
GO:0042107 cytokine metabolic process	2.37	0.0101	52
GO:0046328 regulation of JNK cascade	2.35	0.0101	79
GO:0002474 antigen processing and presentation of peptide antigen via MHC class I	2.46	0.0103	17
GO:0051336 regulation of hydrolase activity	2.32	0.0104	379
GO:0097191 extrinsic apoptotic signaling pathway	2.35	0.0108	36
GO:0034142 toll-like receptor 4 signaling pathway	2.47	0.0108	12
GO:0018212 peptidyl-tyrosine modification	2.31	0.0109	104
GO:0051767 nitric-oxide synthase biosynthetic process	2.46	0.0110	14
GO:0051769 regulation of nitric-oxide synthase biosynthetic process	2.46	0.0110	14
GO:0006152 purine nucleoside catabolic process	2.29	0.0111	334
GO:0046130 purine ribonucleoside catabolic process	2.29	0.0111	334

GO:0070661 leukocyte proliferation	2.30	0.0113	114
GO:0001818 negative regulation of cytokine production	2.31	0.0113	57
GO:0001959 regulation of cytokine-mediated signaling pathway	2.35	0.0114	31
GO:0045944 positive regulation of transcription from RNA polymerase II promoter	2.28	0.0115	399
GO:0070663 regulation of leukocyte proliferation	2.29	0.0117	86
GO:0045429 positive regulation of nitric oxide biosynthetic process	2.39	0.0119	19
GO:0045071 negative regulation of viral genome replication	2.41	0.0119	20
GO:0048699 generation of neurons	2.26	0.0119	418
GO:0010942 positive regulation of cell death	2.27	0.0120	291
GO:0046039 GTP metabolic process	2.26	0.0120	252
GO:0010810 regulation of cell-substrate adhesion	2.29	0.0122	54
GO:0022415 viral reproductive process	2.27	0.0122	102
GO:0009207 purine ribonucleoside triphosphate catabolic process	2.25	0.0123	324
GO:0048666 neuron development	2.25	0.0125	292
GO:1901069 guanosine-containing compound catabolic process	2.25	0.0125	247
GO:0032943 mononuclear cell proliferation	2.26	0.0126	112
GO:0009203 ribonucleoside triphosphate catabolic process	2.25	0.0126	325
GO:0045860 positive regulation of protein kinase activity	2.25	0.0126	174
GO:0032728 positive regulation of interferon-beta production	2.36	0.0127	19
GO:1901068 guanosine-containing compound metabolic process	2.24	0.0130	259
GO:0031589 cell-substrate adhesion	2.24	0.0130	95
GO:0050870 positive regulation of T cell activation	2.26	0.0130	68
GO:0001912 positive regulation of leukocyte mediated cytotoxicity	2.35	0.0130	16
GO:0032944 regulation of mononuclear cell proliferation	2.25	0.0131	84
GO:0009146 purine nucleoside triphosphate catabolic process	2.23	0.0132	326
GO:0002467 germinal center formation	2.37	0.0133	13
GO:0051702 interaction with symbiont	2.34	0.0134	26
GO:0002688 regulation of leukocyte chemotaxis	2.30	0.0135	31

GO:0006809 nitric oxide biosynthetic process	2.30	0.0135	31
GO:0042454 ribonucleoside catabolic process	2.22	0.0135	336
GO:0046330 positive regulation of JNK cascade	2.27	0.0136	28
GO:0043068 positive regulation of programmed cell death	2.22	0.0136	284
GO:0030155 regulation of cell adhesion	2.22	0.0136	138
GO:0061138 morphogenesis of a branching epithelium	2.23	0.0136	74
GO:0051247 positive regulation of protein metabolic process	2.21	0.0137	451
GO:0001763 morphogenesis of a branching structure	2.23	0.0138	79
GO:0009143 nucleoside triphosphate catabolic process	2.21	0.0138	329
GO:0048871 multicellular organismal homeostasis	2.22	0.0138	91
GO:0006184 GTP catabolic process	2.21	0.0139	243
GO:0046209 nitric oxide metabolic process	2.28	0.0140	34
GO:0009205 purine ribonucleoside triphosphate metabolic process	2.20	0.0141	354
GO:0009164 nucleoside catabolic process	2.20	0.0141	337
GO:0016032 viral reproduction	2.21	0.0141	118
GO:0018108 peptidyl-tyrosine phosphorylation	2.21	0.0141	102
GO:0001961 positive regulation of cytokine-mediated signaling pathway	2.40	0.0143	13
GO:0022414 reproductive process	2.19	0.0143	428
GO:0045428 regulation of nitric oxide biosynthetic process	2.26	0.0145	26
GO:0051403 stress-activated MAPK cascade	2.20	0.0145	104
GO:0007254 JNK cascade	2.20	0.0146	87
GO:0030522 intracellular receptor mediated signaling pathway	2.19	0.0147	97
GO:0046651 lymphocyte proliferation	2.19	0.0148	111
GO:0031098 stress-activated protein kinase signaling cascade	2.19	0.0149	107
GO:0040012 regulation of locomotion	2.18	0.0149	240
GO:0044764 multi-organism cellular process	2.19	0.0149	119
GO:0052547 regulation of peptidase activity	2.18	0.0150	149
GO:0032270 positive regulation of cellular protein metabolic process	2.17	0.0151	401

GO:0002040 sprouting angiogenesis	2.28	0.0151	20
GO:0032479 regulation of type I interferon production	2.23	0.0152	29
GO:0009199 ribonucleoside triphosphate metabolic process	2.17	0.0153	357
GO:0001568 blood vessel development	2.17	0.0155	225
GO:0051223 regulation of protein transport	2.16	0.0156	177
GO:0030098 lymphocyte differentiation	2.17	0.0157	142
GO:0043410 positive regulation of MAPK cascade	2.16	0.0157	149
GO:0050670 regulation of lymphocyte proliferation	2.18	0.0157	83
GO:1901658 glycosyl compound catabolic process	2.16	0.0157	341
GO:0072523 purine-containing compound catabolic process	2.15	0.0159	354
GO:0050817 coagulation	2.17	0.0159	67
GO:0032729 positive regulation of interferon-gamma production	2.27	0.0159	15
GO:0050766 positive regulation of phagocytosis	2.22	0.0161	26
GO:2000116 regulation of cysteine-type endopeptidase activity	2.16	0.0161	112
GO:0043406 positive regulation of MAP kinase activity	2.16	0.0162	90
GO:0009154 purine ribonucleotide catabolic process	2.14	0.0163	340
GO:0009261 ribonucleotide catabolic process	2.14	0.0163	340
GO:0045765 regulation of angiogenesis	2.16	0.0164	70
GO:0002685 regulation of leukocyte migration	2.18	0.0165	45
GO:0009968 negative regulation of signal transduction	2.14	0.0165	333
GO:0042089 cytokine biosynthetic process	2.17	0.0166	51
GO:1901342 regulation of vasculature development	2.16	0.0166	73
GO:0032653 regulation of interleukin-10 production	2.32	0.0167	12
GO:0060324 face development	2.22	0.0168	18
GO:0060996 dendritic spine development	2.21	0.0168	19
GO:0002495 antigen processing and presentation of peptide antigen via MHC class II	2.27	0.0171	12
GO:0043065 positive regulation of apoptotic process	2.12	0.0172	281
GO:0009144 purine nucleoside triphosphate metabolic process	2.12	0.0174	359

GO:0007167 enzyme linked receptor protein signaling pathway	2.10	0.0179	268
GO:0060760 positive regulation of response to cytokine stimulus	2.27	0.0180	14
GO:0071347 cellular response to interleukin-1	2.19	0.0180	20
GO:0002828 regulation of type 2 immune response	2.27	0.0181	14
GO:0050863 regulation of T cell activation	2.11	0.0181	98
GO:0045069 regulation of viral genome replication	2.16	0.0182	32
GO:0030198 extracellular matrix organization	2.12	0.0183	51
GO:0043062 extracellular structure organization	2.12	0.0183	51
GO:0000902 cell morphogenesis	2.09	0.0183	385
GO:0045063 T-helper 1 cell differentiation	2.30	0.0186	10
GO:0001916 positive regulation of T cell mediated cytotoxicity	2.24	0.0187	12
GO:0043551 regulation of phosphatidylinositol 3-kinase activity	2.18	0.0187	16
GO:0033674 positive regulation of kinase activity	2.09	0.0188	187
GO:1901698 response to nitrogen compound	2.08	0.0189	222
GO:0035239 tube morphogenesis	2.08	0.0191	133
GO:0001944 vasculature development	2.08	0.0193	236
GO:0043085 positive regulation of catalytic activity	2.07	0.0193	426
GO:0043331 response to dsRNA	2.12	0.0194	31
GO:0009593 detection of chemical stimulus	2.16	0.0194	16
GO:0060998 regulation of dendritic spine development	2.16	0.0194	16
GO:0032495 response to muramyl dipeptide	2.24	0.0194	10
GO:0071357 cellular response to type I interferon	2.29	0.0194	11
GO:0045785 positive regulation of cell adhesion	2.09	0.0194	67
GO:0009166 nucleotide catabolic process	2.07	0.0196	358
GO:0070201 regulation of establishment of protein localization	2.06	0.0198	197
GO:0007599 hemostasis	2.07	0.0201	67
GO:0001952 regulation of cell-matrix adhesion	2.09	0.0202	34
GO:0006195 purine nucleotide catabolic process	2.05	0.0205	349

GO:0032103 positive regulation of response to external stimulus	2.07	0.0205	87
GO:0035924 cellular response to vascular endothelial growth factor stimulus	2.18	0.0205	11
GO:0051050 positive regulation of transport	2.05	0.0206	268
GO:0002709 regulation of T cell mediated immunity	2.12	0.0206	25
GO:0032613 interleukin-10 production	2.18	0.0206	14
GO:0008360 regulation of cell shape	2.06	0.0207	60
GO:1901292 nucleoside phosphate catabolic process	2.04	0.0207	364
GO:0048514 blood vessel morphogenesis	2.05	0.0208	195
GO:0052548 regulation of endopeptidase activity	2.04	0.0210	135
GO:0051046 regulation of secretion	2.04	0.0214	190
GO:0042990 regulation of transcription factor import into nucleus	2.06	0.0214	44
GO:0032648 regulation of interferon-beta production	2.08	0.0215	27
GO:0018193 peptidyl-amino acid modification	2.02	0.0219	398
GO:0007596 blood coagulation	2.04	0.0219	65
GO:0002274 myeloid leukocyte activation	2.03	0.0220	80
GO:1901136 carbohydrate derivative catabolic process	2.02	0.0221	382
GO:0030099 myeloid cell differentiation	2.02	0.0223	174
GO:0043277 apoptotic cell clearance	2.11	0.0227	16
GO:0009141 nucleoside triphosphate metabolic process	2.00	0.0227	367
GO:0042991 transcription factor import into nucleus	2.03	0.0227	46
GO:0002009 morphogenesis of an epithelium	2.01	0.0228	162
GO:0035455 response to interferon-alpha	2.17	0.0229	12
GO:0050673 epithelial cell proliferation	2.01	0.0230	110
GO:0071900 regulation of protein serine/threonine kinase activity	2.00	0.0230	199
GO:0032386 regulation of intracellular transport	2.00	0.0232	158
GO:0051093 negative regulation of developmental process	1.99	0.0235	264
GO:0046822 regulation of nucleocytoplasmic transport	2.00	0.0235	102
GO:0019079 viral genome replication	2.04	0.0235	35

GO:0051250 negative regulation of lymphocyte activation	2.02	0.0235	46
GO:0050777 negative regulation of immune response	2.03	0.0236	35
GO:0043542 endothelial cell migration	2.01	0.0239	47
GO:0002263 cell activation involved in immune response	1.99	0.0242	98
GO:0002366 leukocyte activation involved in immune response	1.99	0.0242	98
GO:0002690 positive regulation of leukocyte chemotaxis	2.04	0.0245	28
GO:0042325 regulation of phosphorylation	1.97	0.0245	461
GO:0010952 positive regulation of peptidase activity	1.98	0.0248	75
GO:0001910 regulation of leukocyte mediated cytotoxicity	2.03	0.0249	21
GO:0046434 organophosphate catabolic process	1.96	0.0252	395
GO:0048754 branching morphogenesis of an epithelial tube	1.97	0.0256	62
GO:0031343 positive regulation of cell killing	2.06	0.0257	19
GO:0050792 regulation of viral reproduction	1.97	0.0260	54
GO:0050727 regulation of inflammatory response	1.96	0.0262	93
GO:0031175 neuron projection development	1.94	0.0265	259
GO:0042116 macrophage activation	1.98	0.0268	29
GO:0043552 positive regulation of phosphatidylinositol 3-kinase activity	2.04	0.0269	12
GO:0043433 negative regulation of sequence-specific DNA binding transcription factor activity	1.94	0.0272	82
GO:0043370 regulation of CD4-positive, alpha-beta T cell differentiation	2.02	0.0273	16
GO:2000514 regulation of CD4-positive, alpha-beta T cell activation	2.02	0.0273	16
GO:0034122 negative regulation of toll-like receptor signaling pathway	2.05	0.0274	11
GO:0008285 negative regulation of cell proliferation	1.92	0.0276	238
GO:0060603 mammary gland duct morphogenesis	1.99	0.0276	16
GO:0048729 tissue morphogenesis	1.92	0.0277	199
GO:0032635 interleukin-6 production	1.94	0.0278	47
GO:0032675 regulation of interleukin-6 production	1.94	0.0278	47
GO:0051701 interaction with host	1.94	0.0278	62
GO:0016045 detection of bacterium	2.03	0.0281	12

GO:0002704 negative regulation of leukocyte mediated immunity	2.04	0.0283	12
GO:0002707 negative regulation of lymphocyte mediated immunity	2.04	0.0283	12
GO:0050708 regulation of protein secretion	1.93	0.0283	65
GO:0010562 positive regulation of phosphorus metabolic process	1.91	0.0283	298
GO:0045937 positive regulation of phosphate metabolic process	1.91	0.0283	298
GO:0043405 regulation of MAP kinase activity	1.91	0.0284	129
GO:0000165 MAPK cascade	1.91	0.0285	270
GO:0006953 acute-phase response	2.04	0.0285	13
GO:0002687 positive regulation of leukocyte migration	1.95	0.0285	36
GO:0007169 transmembrane receptor protein tyrosine kinase signaling pathway	1.90	0.0289	170
GO:0051347 positive regulation of transferase activity	1.90	0.0289	194
GO:0002718 regulation of cytokine production involved in immune response	1.96	0.0290	22
GO:0090218 positive regulation of lipid kinase activity	1.99	0.0291	13
GO:0001890 placenta development	1.91	0.0292	66
GO:0001935 endothelial cell proliferation	1.92	0.0292	39
GO:0002285 lymphocyte activation involved in immune response	1.91	0.0293	60
GO:0002711 positive regulation of T cell mediated immunity	1.97	0.0294	20
GO:0002228 natural killer cell mediated immunity	1.96	0.0295	18
GO:0042267 natural killer cell mediated cytotoxicity	1.96	0.0295	18
GO:0001525 angiogenesis	1.90	0.0296	166
GO:0051341 regulation of oxidoreductase activity	1.92	0.0299	29
GO:0008544 epidermis development	1.90	0.0299	82
GO:0042345 regulation of NF-kappaB import into nucleus	1.95	0.0301	20
GO:0042348 NF-kappaB import into nucleus	1.95	0.0301	20
GO:0032602 chemokine production	1.92	0.0302	29
GO:0070665 positive regulation of leukocyte proliferation	1.90	0.0303	60
GO:0031341 regulation of cell killing	1.95	0.0304	24
GO:0032735 positive regulation of interleukin-12 production	2.05	0.0306	11

GO:0043408 regulation of MAPK cascade	1.88	0.0306	242
GO:0008284 positive regulation of cell proliferation	1.87	0.0308	299
GO:0002715 regulation of natural killer cell mediated immunity	1.98	0.0308	12
GO:0042269 regulation of natural killer cell mediated cytotoxicity	1.98	0.0308	12
GO:2000107 negative regulation of leukocyte apoptotic process	1.94	0.0313	17
GO:0048167 regulation of synaptic plasticity	1.89	0.0316	43
GO:0032989 cellular component morphogenesis	1.86	0.0316	421
GO:0044057 regulation of system process	1.86	0.0317	180
GO:0022604 regulation of cell morphogenesis	1.86	0.0321	166
GO:0043271 negative regulation of ion transport	1.91	0.0322	25
GO:0015807 L-amino acid transport	1.89	0.0323	25
GO:0046835 carbohydrate phosphorylation	1.94	0.0325	12
GO:0032088 negative regulation of NF-kappaB transcription factor activity	1.87	0.0326	48
GO:0060325 face morphogenesis	1.92	0.0327	15
GO:0001932 regulation of protein phosphorylation	1.85	0.0327	418
GO:0060444 branching involved in mammary gland duct morphogenesis	1.93	0.0327	13
GO:0051969 regulation of transmission of nerve impulse	1.85	0.0330	87
GO:0071453 cellular response to oxygen levels	1.88	0.0331	24
GO:0009595 detection of biotic stimulus	1.91	0.0333	17
GO:1901652 response to peptide	1.84	0.0334	117
GO:0043281 regulation of cysteine-type endopeptidase activity involved in apoptotic process	1.82	0.0348	108
GO:0045446 endothelial cell differentiation	1.86	0.0349	22
GO:0044706 multi-multicellular organism process	1.83	0.0350	49
GO:0019058 viral infectious cycle	1.83	0.0350	57
GO:0002695 negative regulation of leukocyte activation	1.83	0.0350	56
GO:0051606 detection of stimulus	1.83	0.0350	50
GO:0051051 negative regulation of transport	1.82	0.0351	145
GO:0048878 chemical homeostasis	1.81	0.0353	375

GO:0034612 response to tumor necrosis factor	1.84	0.0354	41
GO:1901565 organonitrogen compound catabolic process	1.81	0.0355	440
GO:0048608 reproductive structure development	1.81	0.0357	93
GO:0007160 cell-matrix adhesion	1.82	0.0360	61
GO:0006606 protein import into nucleus	1.80	0.0363	137
GO:0044744 protein targeting to nucleus	1.80	0.0363	137
GO:0030100 regulation of endocytosis	1.80	0.0369	88
GO:0030513 positive regulation of BMP signaling pathway	1.89	0.0371	11
GO:0051170 nuclear import	1.79	0.0371	138
GO:2000242 negative regulation of reproductive process	1.82	0.0371	41
GO:0043550 regulation of lipid kinase activity	1.83	0.0373	21
GO:0006935 chemotaxis	1.79	0.0373	144
GO:0060562 epithelial tube morphogenesis	1.79	0.0374	128
GO:0010594 regulation of endothelial cell migration	1.82	0.0375	29
GO:1901699 cellular response to nitrogen compound	1.79	0.0376	134
GO:0050804 regulation of synaptic transmission	1.79	0.0378	77
GO:0048732 gland development	1.79	0.0378	101
GO:0006835 dicarboxylic acid transport	1.82	0.0380	23
GO:0009791 post-embryonic development	1.79	0.0380	62
GO:0042330 taxis	1.78	0.0381	145
GO:0061458 reproductive system development	1.78	0.0383	94
GO:0002832 negative regulation of response to biotic stimulus	1.87	0.0383	12
GO:0006956 complement activation	1.90	0.0384	12
GO:0072376 protein activation cascade	1.90	0.0384	12
GO:0007565 female pregnancy	1.79	0.0387	39
GO:0010812 negative regulation of cell-substrate adhesion	1.84	0.0388	17
GO:0010811 positive regulation of cell-substrate adhesion	1.80	0.0388	32
GO:0050866 negative regulation of cell activation	1.78	0.0390	59

GO:0007507 heart development	1.76	0.0397	185
GO:0043372 positive regulation of CD4-positive, alpha-beta T cell differentiation	1.87	0.0400	11
GO:2000516 positive regulation of CD4-positive, alpha-beta T cell activation	1.87	0.0400	11
GO:0032319 regulation of Rho GTPase activity	1.77	0.0401	46
GO:0002920 regulation of humoral immune response	1.86	0.0402	12
GO:0048730 epidermis morphogenesis	1.81	0.0402	15
GO:0001914 regulation of T cell mediated cytotoxicity	1.83	0.0403	14
GO:0042035 regulation of cytokine biosynthetic process	1.77	0.0405	46
GO:0032642 regulation of chemokine production	1.78	0.0406	27
GO:0002675 positive regulation of acute inflammatory response	1.88	0.0407	12
GO:0046128 purine ribonucleoside metabolic process	1.74	0.0408	395
GO:0032946 positive regulation of mononuclear cell proliferation	1.76	0.0412	58
GO:0060135 maternal process involved in female pregnancy	1.80	0.0415	17
GO:0051130 positive regulation of cellular component organization	1.74	0.0415	309
GO:0009150 purine ribonucleotide metabolic process	1.73	0.0416	418
GO:0060337 type I interferon-mediated signaling pathway	1.88	0.0417	10
GO:0010595 positive regulation of endothelial cell migration	1.81	0.0417	17
GO:0040007 growth	1.73	0.0420	411
GO:0035023 regulation of Rho protein signal transduction	1.74	0.0420	88
GO:2000241 regulation of reproductive process	1.74	0.0422	86
GO:0042993 positive regulation of transcription factor import into nucleus	1.80	0.0423	17
GO:0045766 positive regulation of angiogenesis	1.76	0.0423	39
GO:0042108 positive regulation of cytokine biosynthetic process	1.75	0.0429	33
GO:0051345 positive regulation of hydrolase activity	1.72	0.0430	195
GO:0016358 dendrite development	1.73	0.0431	65
GO:0006163 purine nucleotide metabolic process	1.72	0.0431	436
GO:2001238 positive regulation of extrinsic apoptotic signaling pathway	1.78	0.0432	16
GO:0032609 interferon-gamma production	1.74	0.0436	31

GO:0001894 tissue homeostasis	1.72	0.0438	67
GO:0010950 positive regulation of endopeptidase activity	1.72	0.0440	69
GO:0034655 nucleobase-containing compound catabolic process	1.71	0.0442	462
GO:0071356 cellular response to tumor necrosis factor	1.74	0.0442	29
GO:0043536 positive regulation of blood vessel endothelial cell migration	1.84	0.0443	11
GO:0022612 gland morphogenesis	1.72	0.0444	45
GO:0007044 cell-substrate junction assembly	1.73	0.0446	30
GO:0055082 cellular chemical homeostasis	1.70	0.0451	284
GO:0048771 tissue remodeling	1.71	0.0453	66
GO:0050730 regulation of peptidyl-tyrosine phosphorylation	1.70	0.0458	74
GO:0044092 negative regulation of molecular function	1.69	0.0458	391
GO:0043534 blood vessel endothelial cell migration	1.72	0.0459	30
GO:0032649 regulation of interferon-gamma production	1.72	0.0460	29
GO:0010573 vascular endothelial growth factor production	1.82	0.0461	11
GO:0010574 regulation of vascular endothelial growth factor production	1.82	0.0461	11
GO:0042113 B cell activation	1.69	0.0462	117
GO:0050663 cytokine secretion	1.70	0.0467	51
GO:0048806 genitalia development	1.75	0.0468	13
GO:0002367 cytokine production involved in immune response	1.71	0.0469	27
GO:0090066 regulation of anatomical structure size	1.68	0.0469	166
GO:0002700 regulation of production of molecular mediator of immune response	1.71	0.0469	35
GO:0046636 negative regulation of alpha-beta T cell activation	1.79	0.0470	11
GO:0046660 female sex differentiation	1.69	0.0472	47
GO:0035295 tube development	1.67	0.0480	185
GO:0036294 cellular response to decreased oxygen levels	1.70	0.0481	22
GO:0071456 cellular response to hypoxia	1.70	0.0481	22
GO:0010939 regulation of necrotic cell death	1.74	0.0482	12
GO:0001678 cellular glucose homeostasis	1.69	0.0486	30

GO:0042278 purine nucleoside metabolic process	1.66	0.0486	398
GO:0002712 regulation of B cell mediated immunity	1.72	0.0491	20
GO:0002889 regulation of immunoglobulin mediated immune response	1.72	0.0491	20
GO:0001936 regulation of endothelial cell proliferation	1.68	0.0492	34
GO:0050871 positive regulation of B cell activation	1.68	0.0493	44
GO:0050671 positive regulation of lymphocyte proliferation	1.67	0.0493	57
GO:0050921 positive regulation of chemotaxis	1.68	0.0493	40
GO:0042306 regulation of protein import into nucleus	1.66	0.0494	80
GO:0048010 vascular endothelial growth factor receptor signaling pathway	1.70	0.0495	17
GO:0007610 behavior	1.65	0.0495	217
GO:0003158 endothelium development	1.68	0.0498	24
GO:0007162 negative regulation of cell adhesion	1.66	0.0498	47
**Down-regulated**			
GO:0000279 M phase	-5.26	9.73E-08	349
GO:0022403 cell cycle phase	-5.06	2.58E-07	470
GO:0000280 nuclear division	-4.86	8.02E-07	260
GO:0007067 mitosis	-4.86	8.02E-07	260
GO:0000087 M phase of mitotic cell cycle	-4.84	8.51E-07	266
GO:0048285 organelle fission	-4.60	2.63E-06	276
GO:0006281 DNA repair	-4.57	3.07E-06	305
GO:0007059 chromosome segregation	-4.36	1.03E-05	123
GO:0000278 mitotic cell cycle	-4.24	1.23E-05	438
GO:0006260 DNA replication	-4.27	1.25E-05	193
GO:0051301 cell division	-4.05	2.82E-05	359
GO:0006396 RNA processing	-3.92	4.75E-05	467
GO:0006261 DNA-dependent DNA replication	-3.26	0.0007	65
GO:0000226 microtubule cytoskeleton organization	-3.20	0.0007	219

GO:0006974 response to DNA damage stimulus	-3.15	0.0008	434
GO:0034470 ncRNA processing	-3.10	0.0011	180
GO:0007017 microtubule-based process	-3.00	0.0014	301
GO:0007131 reciprocal meiotic recombination	-3.27	0.0014	20
GO:0035825 reciprocal DNA recombination	-3.27	0.0014	20
GO:0051297 centrosome organization	-3.02	0.0016	55
GO:0006364 rRNA processing	-2.95	0.0019	98
GO:0042254 ribosome biogenesis	-2.88	0.0022	144
GO:0010564 regulation of cell cycle process	-2.78	0.0028	254
GO:0016072 rRNA metabolic process	-2.80	0.0029	101
GO:0007126 meiosis	-2.79	0.0029	82
GO:0051327 M phase of meiotic cell cycle	-2.79	0.0029	82
GO:0051321 meiotic cell cycle	-2.73	0.0035	87
GO:0008380 RNA splicing	-2.71	0.0036	220
GO:0007127 meiosis I	-2.75	0.0037	43
GO:0050000 chromosome localization	-2.91	0.0039	17
GO:0051303 establishment of chromosome localization	-2.91	0.0039	17
GO:0031023 microtubule organizing center organization	-2.68	0.0042	59
GO:0000070 mitotic sister chromatid segregation	-2.71	0.0045	38
GO:0022613 ribonucleoprotein complex biogenesis	-2.63	0.0045	191
GO:0007346 regulation of mitotic cell cycle	-2.61	0.0047	177
GO:0006397 mRNA processing	-2.58	0.0051	275
GO:0051310 metaphase plate congression	-2.80	0.0054	15
GO:0000819 sister chromatid segregation	-2.62	0.0056	41
GO:0006310 DNA recombination	-2.54	0.0058	140
GO:0051983 regulation of chromosome segregation	-2.66	0.0062	20
GO:0045132 meiotic chromosome segregation	-2.71	0.0065	15
GO:0034660 ncRNA metabolic process	-2.48	0.0067	228

GO:0016071 mRNA metabolic process	-2.41	0.0081	318
GO:0051320 S phase	-2.44	0.0086	40
GO:0006302 double-strand break repair	-2.40	0.0089	94
GO:0015936 coenzyme A metabolic process	-2.66	0.0091	11
GO:0007051 spindle organization	-2.36	0.0099	64
GO:0033865 nucleoside bisphosphate metabolic process	-2.51	0.0109	13
GO:0033875 ribonucleoside bisphosphate metabolic process	-2.51	0.0109	13
GO:0034032 purine nucleoside bisphosphate metabolic process	-2.51	0.0109	13
GO:0032886 regulation of microtubule-based process	-2.30	0.0115	82
GO:0034502 protein localization to chromosome	-2.28	0.0154	20
GO:0033261 regulation of S phase	-2.14	0.0184	29
GO:0000084 S phase of mitotic cell cycle	-2.13	0.0194	32
GO:0006270 DNA replication initiation	-2.21	0.0196	16
GO:0007080 mitotic metaphase plate congression	-2.22	0.0207	11
GO:0006695 cholesterol biosynthetic process	-2.06	0.0223	29
GO:0000725 recombinational repair	-2.03	0.0230	44
GO:0019395 fatty acid oxidation	-1.99	0.0251	50
GO:0034440 lipid oxidation	-1.98	0.0252	51
GO:0070925 organelle assembly	-1.97	0.0252	90
GO:0008608 attachment of spindle microtubules to kinetochore	-2.06	0.0253	17
GO:0007098 centrosome cycle	-1.99	0.0254	33
GO:0045005 maintenance of fidelity involved in DNA-dependent DNA replication	-2.04	0.0268	12
GO:0048704 embryonic skeletal system morphogenesis	-1.98	0.0270	25
GO:0009124 nucleoside monophosphate biosynthetic process	-1.95	0.0296	24
GO:0051225 spindle assembly	-1.92	0.0296	35
GO:0045454 cell redox homeostasis	-1.91	0.0301	44
GO:0010165 response to X-ray	-1.96	0.0309	16
GO:0000724 double-strand break repair via homologous recombination	-1.87	0.0333	43

GO:0006275 regulation of DNA replication	-1.85	0.0336	67
GO:0070507 regulation of microtubule cytoskeleton organization	-1.84	0.0341	70
GO:0030810 positive regulation of nucleotide biosynthetic process	-1.88	0.0343	19
GO:1900373 positive regulation of purine nucleotide biosynthetic process	-1.88	0.0343	19
GO:1900544 positive regulation of purine nucleotide metabolic process	-1.88	0.0343	19
GO:0010257 NADH dehydrogenase complex assembly	-1.87	0.0392	14
GO:0032981 mitochondrial respiratory chain complex I assembly	-1.87	0.0392	14
GO:0097031 mitochondrial respiratory chain complex I biogenesis	-1.87	0.0392	14
GO:0007076 mitotic chromosome condensation	-1.89	0.0396	11
GO:0071103 DNA conformation change	-1.75	0.0405	99
GO:0034453 microtubule anchoring	-1.78	0.0406	32
GO:0009163 nucleoside biosynthetic process	-1.75	0.0412	68
GO:0042455 ribonucleoside biosynthetic process	-1.75	0.0412	68
GO:1901659 glycosyl compound biosynthetic process	-1.75	0.0412	68
GO:0006298 mismatch repair	-1.82	0.0414	12
GO:0006301 postreplication repair	-1.80	0.0423	15
GO:0009156 ribonucleoside monophosphate biosynthetic process	-1.78	0.0423	22
GO:0045981 positive regulation of nucleotide metabolic process	-1.77	0.0426	20
GO:0007099 centriole replication	-1.82	0.0427	13
GO:0007090 regulation of S phase of mitotic cell cycle	-1.78	0.0427	21
GO:0090329 regulation of DNA-dependent DNA replication	-1.77	0.0432	19
GO:0071156 regulation of cell cycle arrest	-1.72	0.0433	134
GO:0031109 microtubule polymerization or depolymerization	-1.73	0.0440	44
GO:0051276 chromosome organization	-1.71	0.0440	500
GO:0007088 regulation of mitosis	-1.72	0.0441	46
GO:0051783 regulation of nuclear division	-1.72	0.0441	46
GO:0048701 embryonic cranial skeleton morphogenesis	-1.76	0.0451	14
GO:0033108 mitochondrial respiratory chain complex assembly	-1.74	0.0462	19

GO:0030261 chromosome condensation	-1.73	0.0468	22
GO:0070192 chromosome organization involved in meiosis	-1.73	0.0477	14
GO:0065002 intracellular protein transmembrane transport	-1.71	0.0478	25
GO:0071806 protein transmembrane transport	-1.71	0.0478	25
GO:0000075 cell cycle checkpoint	-1.66	0.0489	118
GO:0044743 intracellular protein transmembrane import	-1.70	0.0491	24
